# A strong bimetal-support interaction in ethanol steam reforming

**DOI:** 10.1038/s41467-023-38883-x

**Published:** 2023-06-02

**Authors:** Hao Meng, Yusen Yang, Tianyao Shen, Wei Liu, Lei Wang, Pan Yin, Zhen Ren, Yiming Niu, Bingsen Zhang, Lirong Zheng, Hong Yan, Jian Zhang, Feng-Shou Xiao, Min Wei, Xue Duan

**Affiliations:** 1 State Key Laboratory of Chemical Resource Engineering, Beijing Advanced Innovation Center for Soft Matter Science and Engineering, Beijing University of Chemical Technology, Beijing, 100029 P. R. China; 2 Shenyang National Laboratory for Materials Science, Institute of Metal Research, Chinese Academy of Sciences, Shenyang, 110016 P. R. China; 3 Institute of High Energy Physics, Chinese Academy of Sciences, Beijing, 100049 P. R. China; 4 Key Lab of Biomass Chemical Engineering of Ministry of Education, College of Chemical and Biological Engineering, Zhejiang University, Hangzhou, 310027 P. R. China

**Keywords:** Heterogeneous catalysis, Catalytic mechanisms, Chemical engineering

## Abstract

The metal-support interaction (MSI) in heterogeneous catalysts plays a crucial role in reforming reaction to produce renewable hydrogen, but conventional objects are limited to single metal and support. Herein, we report a type of RhNi/TiO_2_ catalysts with tunable RhNi-TiO_2_ strong bimetal-support interaction (SBMSI) derived from structure topological transformation of RhNiTi-layered double hydroxides (RhNiTi-LDHs) precursors. The resulting 0.5RhNi/TiO_2_ catalyst (with 0.5 *wt*.% Rh) exhibits extraordinary catalytic performance toward ethanol steam reforming (ESR) reaction with a H_2_ yield of 61.7%, a H_2_ production rate of 12.2 L h^−1^ g_cat_^−1^ and a high operational stability (300 h), which is preponderant to the state-of-the-art catalysts. By virtue of synergistic catalysis of multifunctional interface structure (Rh-Ni^*δ*−^-O_*v*_-Ti^3+^; O_*v*_ denotes oxygen vacancy), the generation of formate intermediate (the rate-determining step in ESR reaction) from steam reforming of CO and CH_*x*_ is significantly promoted on 0.5RhNi/TiO_2_ catalyst, accounting for its ultra-high H_2_ production.

## Abstract (web summary)

Ethanol steam reforming (ESR) reaction is an environmentally benign and sustainable route for H_2_ production. Herein, the authors demonstrate a conceptional strategy for rational design and preparation of highly-efficient catalyst toward ESR *via* an RhNi-TiO_2_ strong bimetal-support interaction.

## Introduction

Supported metal catalysts with metal-support interaction (MSI) have been widely demonstrated in heterogeneous catalysis for many industrially important reactions (e.g., hydrogenation, oxidation, and catalytic reforming)^1–3^. Especially, the strong metal-support interaction (SMSI), a term to describe the phenomenon that the geometric and electronic structure of metal species is modified through interaction with supports has attracted considerable research interest for decades. More recently, Bao, Zhang, de Jong, and Christopher et al. reported a number of new strategies to regulate SMSI including redox-regulated (Pt/TiO_2_, Co/ Nb_2_O_5_)^4,5^, particle size-controlled (Ir/CeO_2_, Ru/TiO_2_)^6,7^, adsorbate-mediated (Ni/TiO_2_, Ru@MoO_3−*x*_, and Rh/Nb_2_O_5_)^8,9^, soft/wet chemistry-assisted (Au@TiO_2_)^10,11^ and crystal phase/facet-guided catalysts (Ru/TiO_2_ and Pd/TiO_2_)^12,13^. In these cases, effective supports with engineered characteristics have been successfully employed to stabilize metal species, modify its geometric/electronic structure, and regulate mass transfer efficiency. Importantly, the proximity of active metal species to support defects also makes a great contribution to promote the activation adsorption of reactants, optimize the transition state of adsorbates and facilitate the transformation of reaction intermediates^14–16^. In general, the SMSI involved in heterogeneous metal catalysts plays a critical role in boosting catalytic performance toward structure-sensitive reactions.

Hydrogen (H_2_) with a high energy density and zero pollution is being contemplated as the most promising alternative energy, which has been globally explored based on steam reforming of hydrocarbons and oxygenates (e.g., CH_4_, CO, CH_3_OH, and CH_3_CH_2_OH) in the preceding decades^3,17–29^. Ethanol, with low toxicity and high hydrogen/carbon ratio, which can be facilely produced from renewable biomass, has been employed as a hydrogen feedstock through ethanol steam reforming (ESR) reaction^18,24–29^. In respect to such a structure-sensitive reaction, the metal active sites are geometrically and electronically modified *via* SMSI, and the supports in most cases participate in the catalytic reaction^2,15^. Nevertheless, the conventionally reported SMSI is normally focused on individual metal and oxide support, whose single-channel interaction shows restriction when applied in certain complex processes^4,16^, such as ESR reaction involving multiple sequential steps. Therefore, how to develop sophisticated catalysts based on multichannel SMSI with largely enhanced catalytic performance for ESR still remains a huge challenge. Meanwhile, an in-depth insight into interfacial active sites at the atomic level is crucial for understanding reaction mechanism and pathway.

In this work, we prepared a series of *x*RhNi/TiO_2_ catalysts with tunable strong bimetal-support interactions (SBMSI) based on a topotactic transformation from RhNiTi-layered double hydroxides (RhNiTi-LDHs) precursors. The CO-DRIFT and STEM confirm the existence of a significantly reversible TiO_2_ coating over RhNi bimetallic nanoparticle. The coordination and electronic structure between RhNi bimetal and TiO_2_ support are facilely regulated *via* changing the doping content of Rh, which realizes a multi-transfer pathway of electrons and further optimizes the interfacial active sites (Rh-Ni^*δ*−^-O_*v*_-Ti^3+^). The resulting 0.5RhNi/TiO_2_ (with 0.5 *wt*.% Rh) sample exhibits a prominent catalytic performance toward ESR reaction, with an ethanol conversion of 99.7% and a H_2_ yield of 61.7% at 400 °C. The H_2_ production rate reaches 12.2 L h^−1^ g_cat_^−1^ with an outstanding catalysis stability within 300 h, which is preponderant to the state-of-the-art catalysts. The steam reforming of CO and CH_*x*_, which is the crucial step affecting H_2_ production in ESR, is largely boosted at the interfacial sites (Rh-Ni^*δ*−^-O_*v*_-Ti^3+^). Kinetic studies combined with in situ characterizations (XAFS and FT-IR) and DFT theoretical calculations demonstrate that the SBMSI in 0.5RhNi/TiO_2_ catalyst not only promotes the formation of formate intermediate (the rate-determining step), but also alleviates the strong binding of formate and CO_2_ on catalyst surface, both of which are beneficial to the transformation of reactants and the regeneration of active sites.

## Results and discussion

### Synthesis and characterization of *x*RhNi/TiO_2_ catalysts

The XRD patterns show a series of typical characteristic diffraction peaks of hydrotalcite-like structure (003, 006, 012, and 110) in as-synthesized *x*RhNiTi-LDHs precursors (Supplementary Fig. 1a). As shown in the SEM images (Supplementary Fig. S2), the introduction of trace Rh does not significantly affect its surface topography, and all these samples display a frizzy flowerlike morphology. After a treatment in H_2_ atmosphere at 400 °C, the crystallite surface becomes rough with enhanced specific surface area and enriched pore structure (Supplementary Fig. 3 and Supplementary Table 1). The crystal structure of as-obtained *x*RhNi/TiO_2_ samples is indexed to a superimposition of face-centered cubic (fcc) Ni (JCPDS 7440-02-0) and anatase TiO_2_ phase (JCPDS 21-1272) (Supplementary Fig. 1b). Transmission electron microscopy (TEM) and high-resolution transmission electron microscope (HR-TEM) images of *x*RhNi/TiO_2_ samples show that Ni nanoparticles (particle size: 12−16 nm) are uniformly dispersed within the TiO_2–*x*_ matrix (Supplementary Figs. 4 and 5). Especially, a well-defined metal-support (Ni-TiO_2_) interface structure is clearly observed in these *x*RhNi/TiO_2_ samples (Supplementary Fig. 5 and Supplementary Note 1).

### Catalytic performance and kinetic analysis toward ESR

**Fig. 1:**
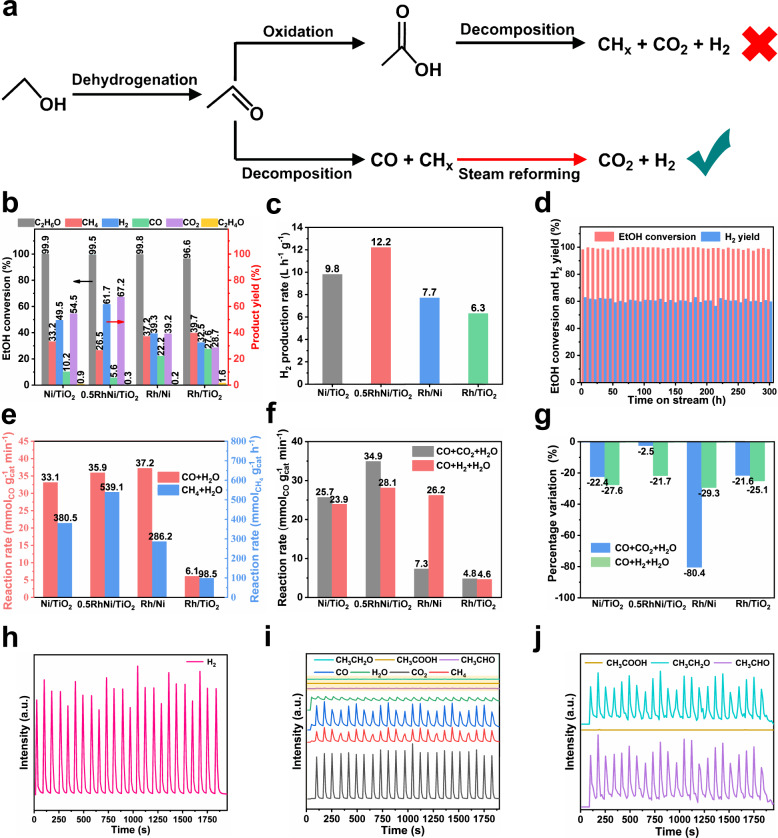
Catalytic performance toward ESR reaction and kinetic studies on CO and CH_4_ reforming. **a** Schematic representation for the reaction paths of ESR reaction (Red arrow indicates the key step). **b** Ethanol conversion, products yield and **c** H_2_ production rate over various catalysts (reaction conditions: catalyst (0.15 g) + SiO_2_ (1.50 g); liquid feed of S/C = 3 at 0.060 mL min^−1^; N_2_ carrier at 50.0 mL min^−1^; reaction temperature: 400 °C; time on stream: 1.5 h). **d** Time on stream (TOS) test of 0.5RhNi/TiO_2_ catalyst at 400 °C. **e**, **f** Reaction rate for CO/CH_4_ steam reforming reaction within kinetic range (CO or CH_4_ conversion <10%). Reaction conditions: catalyst (5 mg) + SiO_2_ (50 mg), liquid feed of H_2_O at 0.032 mL min^−1^, CO at 50.0 mL min^−1^, CH_4_ at 10 mL min^−1^, CO_2_/H_2_ at 10.0 mL min^−1^, N_2_ carrier at 50 mL min^−1^, reaction temperature: 400 °C, time on stream: 0.5 h. **g** Percentage variation in reaction rate of CO steam reforming with CO_2_/H_2_ addition over Ni/TiO_2_, 0.5RhNi/TiO_2_, Rh/Ni, and Rh/TiO_2_ catalysts, respectively. **h**, **i** MS signals from the *operando* pulse experiment of ethanol and water (S/C = 3) over 0.5RhNi/TiO_2_ catalyst at 400 °C. **j** Enlarged view for MS signals of the three species in **i**.

The ESR reaction was performed in a fixed-bed reactor with steam/carbon (S/C) ratio of 3. Compared with Ni/TiO_2_ and Rh/TiO_2_, the RhNi/TiO_2_ and Rh/Ni catalysts remarkably promote the conversion of ethanol and acetaldehyde at 350 °C (Supplementary Figs. 6a, b), indicating the advantages of RhNi bimetal system. As the reaction temperature reaches 400 °C, an almost complete conversion of ethanol and acetaldehyde is obtained for all these catalysts, but the distribution of gas product (CO, CO_2_, CH_4_, and H_2_) is significantly different (Fig. 1b and Supplementary Fig. 6c). This indicates that the cleavage of C−C, O−H and C−H bonds in ethanol and acetaldehyde is sensitive to reaction temperature, and 400 °C is required for the breakage of the chemical bonds above. For the *x*RhNi/TiO_2_ catalysts, the H_2_ yield shows a volcanic trend as Rh content increases from 0 to 1%, and the maximum value is present in 0.5RhNi/TiO_2_ sample (H_2_ yield: 61.7%; production rate: 12.2 L h^−1^ g^−1^) (Fig. 1c and Supplementary Fig. 7), which is preponderant to the state-of-the-art catalysts for ESR (Supplementary Table 2). Furthermore, we also tested the ESR reaction at a higher WHSV (21 h^−1^) and GHSV (16700 h^−1^) at 400 °C (ethanol conversion less than 70%), and the 0.5RhNi/TiO_2_ catalyst still displayed the optimal hydrogen yield and relatively low CH_4_ and CO yields (Supplementary Fig. 8). This is in sharp contrast to the Rh/NiO and Rh/TiO_2_ samples, which give a much lower H_2_ yield but a higher proportion of CO and CH_4_ species (Fig. 1b), showing the superiority of bimetal-support (RhNi-TiO_2_) system. In addition, the time on stream (TOS) tests for Ni/TiO_2_, Rh/Ni, Rh/TiO_2_, and 0.5RhNi/TiO_2_ were carried out at 400 °C, respectively. After the reaction for 40 h, the ethanol conversion over Ni/TiO_2_, Rh/Ni, and Rh/TiO_2_ decreases from 100% to 91.33%, 58.54%, and 74.16%, respectively (Supplementary Fig. 9). In contrast, both the ethanol conversion and hydrogen yield in the presence of 0.5RhNi/TiO_2_ catalyst remain stable within 300 h (Fig. 1d). The crystal structure and particle size of the used catalyst do not show obvious change except for the formation of certain carbon-accumulating species (Supplementary Fig. 10).

The *operando* infrared spectra (Supplementary Figs. 11–13 and Supplementary Note 2) were performed to identify the ESR reaction path. The results from the infrared spectra verify that the ESR reaction over 0.5RhNi/TiO_2_, Ni/TiO_2_, and Rh/Ni catalysts undergoes ethanol dehydrogenation to acetaldehyde followed by acetaldehyde decomposition to CO and CH_*x*_ (CO/CH_*x*_-mediated reforming process), rather than the acetate paths (Fig. 1a)^24,28,29^. The resulting CO and CH_*x*_ as key intermediates continue to react with H_2_O to produce CO_2_ and H_2_. This is consistent with the results from the *operando* pulse experiment of ethanol and water (Fig. 1h–j) with mass spectrometer (MS), where the characteristic bands assigned to various reaction intermediates are observed whilst acetate species are not detected during the whole reaction process. Thus, the H_2_ yield depends heavily on the transformation of these CO and CH_*x*_ intermediates, in addition to ethanol conversion.

Furthermore, we performed kinetic tests on ethanol dehydrogenation, acetaldehyde decomposition, steam reforming of CO (or CH_4_) to study the C−H bond cleavage, C−C bond cleavage, CO or CH_*x*_ transformation during ESR reaction. As shown in Supplementary Fig. 14a, the apparent activation energy of these reaction processes gives the following sequence: ethanol dehydrogenation (44.54 kJ mol^−1^) < acetaldehyde decomposition (50.45 kJ mol^−1^) < CO steam reforming (89.46 kJ mol^−1^) < CH_4_ steam reforming (101.26 kJ mol^−1^), which indicates that the cleavage of C−H and C−C bonds in ethanol is facile whilst the transformation of intermediates (CO and CH_*x*_) is rather difficult. This result is further demonstrated through a more significantly concentration-dependent reaction order for CO and CH_4_ in comparison with ethanol and acetaldehyde: ethanol (0.46) < acetaldehyde (0.62) < CO (1.18) < CH_4_ (1.23) (Supplementary Fig. 14b). In addition, we contrasted the conversion rates of CO and CH_4_ over these samples (Fig. 1e). The CO conversion declines in the following sequence: Rh/Ni > 0.5RhNiTi > NiTi >> Rh/TiO_2_; whilst CH_4_ conversion decreases in order: 0.5RhNiTi > NiTi > Rh/Ni > Rh/TiO_2_. The higher CO conversion rate on Rh/Ni is not consistent with its high CO yield. Considering that the reaction mixture would reach equilibrium at prolonged reaction time, and the products (H_2_ and CO_2_) may affect the progress of main reaction, we measured the reaction rate of water-gas shift (WGS) reaction by adding a small amount of H_2_ or CO_2_ into the reaction atmosphere (Fig. 1f). A similar decrease extent is observed for these catalysts after H_2_ introduction (Fig. 1g), indicating that the inhibitory effect mainly originates from the equilibrium limit (Le Chatelier’s principle). In contrast, these catalysts show a significantly difference in CO reaction rate after CO_2_ introduction: a remarkable decline ratio of 80.4% (from 37.2 to 7.3 mol CO g_cat_^−1^ min^−1^) is obtained over Rh/Ni catalyst, relative to 0.5RhNi/TiO_2_ with a slight decrease of 2.5%, indicating the existence of other important factors besides equilibrium limit. Moreover, the CO_2_-TPD (Supplementary Fig. 15 and Supplementary Note 3) and reaction order measurements (Supplementary Fig. 16 and Supplementary Note 4) confirm that Rh/Ni catalyst shows a stronger CO_2_ adsorption ability and a more negative CO_2_ reaction order (−0.64) compared with 0.5RhNi/TiO_2_ (−0.18), demonstrating a stronger inhibitory action by CO_2_ in the former case. In addition, the conversion of CO and CH_4_ are also determined when H_2_O, CO, CH_4_, H_2_, and CO_2_ are present in the reactants simultaneously (Supplementary Fig. 17 and Supplementary Note 5), and 0.5RhNi/TiO_2_ catalyst still exhibits the highest conversion of CH_4_ and CO. Thus, the kinetic studies above substantiate that 0.5RhNi/TiO_2_ catalyst possesses a powerful catalytic transformation ability toward CO and CH_*x*_ as well as a strong resistance against CO_2_, resulting in its extraordinary catalytic performance toward ESR.

### Microstructure investigations and fine-structure characterizations

**Fig. 2:**
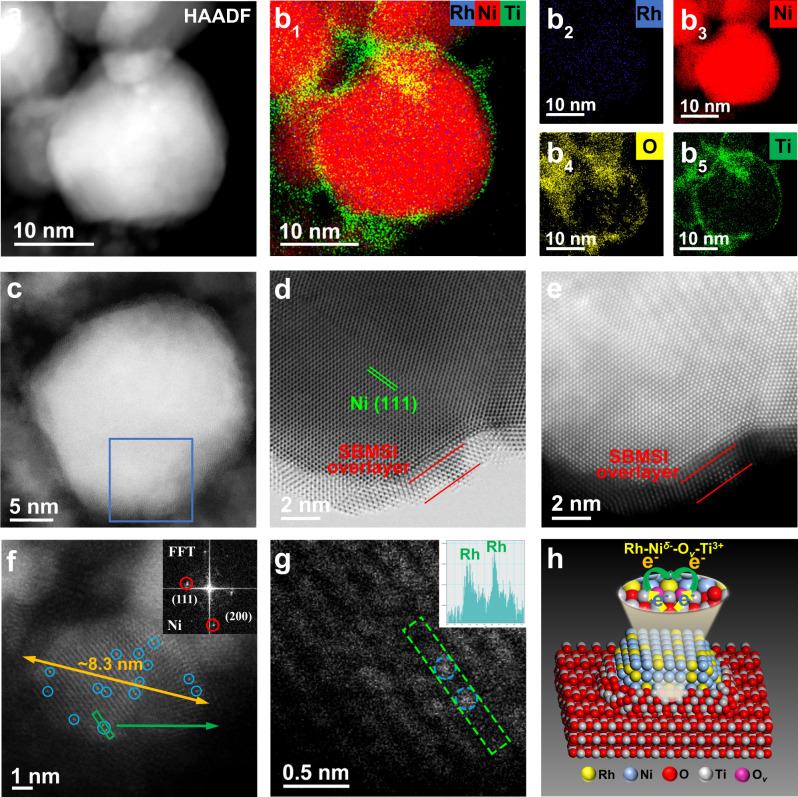
Microstructure fine-structure characterizations. **a**, **c**, **d** ac-HAADF-STEM and **e** BF-STEM images, **b**_**1**_–**b**_**5**_ corresponding EDS mapping, and **f**, **g** high-resolution STEM images of the 0.5RhNi/TiO_2_ catalyst with atomically dispersed Rh species marked by blue circles. **h** Schematic diagram of the 0.5RhNi/TiO_2_ catalyst.

Aberration-corrected high-angle annular dark-field scanning transmission electron microscopy (ac-HAADF-STEM) image and energy-dispersive spectroscopy (EDS) measurements were carried out to explore the interfacial structure of *x*RhNi/TiO_2_ samples (Fig. 2 and Supplementary Figs. 18–23). For 0.3RhNi/TiO_2_ and 0.5RhNi/TiO_2_ catalysts, Rh tends to present an atomic-level dispersion on the surface of Ni particles to form RhNi bimetallic structure (Fig. 2f, g, and Supplementary Fig. 18), and a significant interface encapsulation of TiO_2_ on the surrounding of RhNi nanoparticle is observed after H_2_ activation (Fig. 2b_1_–b_5_, d, e, and Supplementary Fig. 20). However, visible TiO_2-*x*_ overlayer recedes over RuNi bimetallic NPs when exposed to air oxidation conditions (Supplementary Fig. 21), which is consistent with the phenomenon from classical SMSI^4,9,13,16,30–32^. For 0.8RhNi/TiO_2_ and 1.0RhNi/TiO_2_ sample with a higher Rh content, Rh species shows an aggregation state on the surface of Ni particles (Supplementary Figs. 22 and 23). The results intuitively confirm the well-defined interface structure between RhNi bimetal and TiO_2_ support. In addition, Ni/TiO_2_ and Rh/TiO_2_ samples also show classic SMSI effect as demonstrated by STEM and elemental line scanning, in which the reversible overlayer of TiO_2–*x*_ appears and disappears under the reduction and oxidation conditions, respectively (Supplementary Figs. 24–26).

**Fig. 3:**
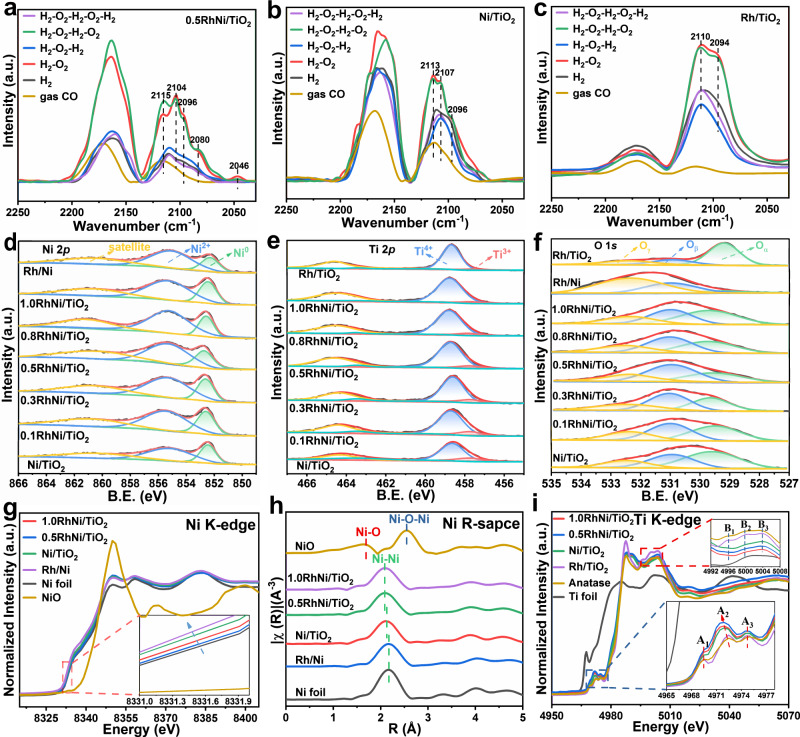
Geometric and electronic properties characterizations. CO-DRIFT spectra at ambiance temperature over **a** 0.5RhNi/TiO_2_, **b** Ni/TiO_2_, and **c** Rh/TiO_2_ catalysts during five-cycle H_2_ reduction-O_2_ oxidation. XPS spectra of **d** Ni 2*p*, **e** Ti 2*p*, and **f** O 1 *s* for various samples. Normalized spectra of **g** Ni K-edge XANES, **h** Ni K-edge EXAFS at R-space, and **i** Ti K-edge XANES for various samples.

The CO-DRIFT was used to further identify the structure evolution as demonstrated above (Fig. 3a–c, and Supplementary Fig. 27). The peaks at 2115, 2104, and 2096 cm^−1^ are assigned to the CO adsorbed at Ni and Rh sites, and the bands at 2080 and 2046 cm^−1^ are attributed to RhNi bimetallic interface sites. The alternative changes of weakened and strengthened CO adsorption intensity during five-cycle of H_2_ reduction-O_2_ oxidation correspond to a reversible encapsulation and decapsulation of TiO_2-*x*_ overlayer. Notably, a more pronounced variation is observed over 0.5RhNi/TiO_2_ catalyst, signifying a stronger interaction from RhNi bimetal and TiO_2_ support. For Rh/Ni sample, the Rh clusters are dispersed on Ni without SMSI effect (Supplementary Figs. 27 and 28).

The electronic structure of *x*RhNi/TiO_2_ samples was investigated by X-ray photoelectron spectroscopy (XPS). As the Rh content increases from 0 to 0.5%, the binding energy of Ni 2*p* moves to higher energy, indicating a decreased Ni electron density on 0.5RhNi/TiO_2_ catalyst (Fig. 3d and Supplementary Note 6). However, with a further increase of Rh loading from 0.5% to 1.0%, the binding energy of Ni shifts back to lower energy. The binding energy of Rh exhibits an opposite variation relative to Ni (Supplementary Fig. 29). The results indicate that the electron transfer from Ni to Rh becomes weak when the Rh distribution converts from atomic level to clusters or particles. This volcanic change trend of electron density for Ni species verifies that this bimetal-support interface breaks through the traditional electron transfer mode between single metal and oxide support. Compared with Ni/TiO_2_, the Ti^3+^/(Ti^3+^+Ti^4+^) ratio in *x*RhNi/TiO_2_ samples declines gradually with the increment of Rh loading (Fig. 3e). Notably, the Ti^3+^ species in Rh/TiO_2_ displays a significant reduction compared with Ni/TiO_2_, indicating Ti^3+^ mainly originates from Ni-TiO_2–*x*_ interaction and the formation of RhNi bimetal would change such interaction. In addition, the O_*β*_/(O_*α*_ + O_*β*_ + O_*γ*_) ratio (O_*α*_: lattice oxygen O^2−^; O_*β*_: chemisorbed oxygen O^−^_2_ or O^−^; O_*γ*_: other oxygen species including adsorbed water) shows a rise firstly and then a decline (Fig. 3f), and the maximal value is present in 0.5RhNi/TiO_2_ (Supplementary Table 3), indicating that a lower Ni electron density promotes the combination of oxygen species. The variation in RhNi-TiO_2_ interaction was further proved by H_2_-TPR and H_2_-TPD analysis (Supplementary Fig. 30 and Supplementary Note 7). The results above demonstrate that the electronic effect in *x*RhNi/TiO_2_ samples can be finely regulated through tuning Rh content.

In addition, the interface electronic and coordination structure were studied *via* X-ray absorption spectroscopy (XAS). As shown in normalized XANES spectra of Ni K-edge (Fig. 3g), the absorption edge of all samples is located at lower photon energy relative to Ni foil, indicating the existence of a negative valence state (Ni^*δ*−^ species), which is similar to other TiO_2_-supported group VIII metals with electron transfer from TiO_2–*x*_ to the interfacial metal atoms^4,9,16,30–32^. Notably, the absorption edge of 0.5RhNi/TiO_2_ displays a minimum shift to lower energy among these samples, indicative of the lowest electron density of interfacial Ni atom. Meanwhile, the fitting results from FT *k*^3^-weighted Fourier transforms of the extended X-ray absorption fine-structure (EXAFS) spectra and wavelet transforms (Fig. 3h, Supplementary Figs. 31 and 32, and Supplementary Table 4) show that the Ni−Ni bond length in Ni/TiO_2_ sample becomes shorter compared with Ni foil and Rh/Ni, owing to the interaction between Ni and TiO_2_ support. Moreover, the Ni−Ni bond length in 0.5RhNi/TiO_2_ and 1.0RhNi/TiO_2_ shortens further relative to Ni/TiO_2_.

The normalized Ti K-edge XANES data are presented in Fig. 3i. All the samples show similar curves to the anatase reference in pre-edge range (4968 to 4980 eV) and post-edge region (4992 to 5008 eV), but the A_1_ peak and the three peaks (denoted as B_1_, B_2_ and B_3_) for Ni/TiO_2_, 0.5RhNi/TiO_2_ and 1.0RhNi/TiO_2_ samples are less resolved; and the intensity of their A_2_ peaks becomes stronger accompanied with a shift to lower energy. The results indicate the presence of distorted octahedral Ti−O environment associated with oxygen vacancies^13,33^. Fourier transform of Ti K-edge EXAFS spectra display that the first shell of Ti−O bond in *x*RhNi/TiO_2_ samples gives an obviously less than sixfold coordination number (Supplementary Fig. 33, Supplementary Table 5 and Supplementary Note 8), which is the most significant for 0.5RhNi/TiO_2_. Based on the results afore-mentioned, a unique geometric and electronic structure between RhNi bimetal and TiO_2_ support in *x*RhNi/TiO_2_ catalysts is demonstrated clearly (Fig. 2h), defined as the strong bimetallic-support interaction (SBMSI), which is responsible for the outstanding catalytic performance of 0.5RhNi/TiO_2_.

### In situ spectral characterization for reaction mechanism

**Fig. 4:**
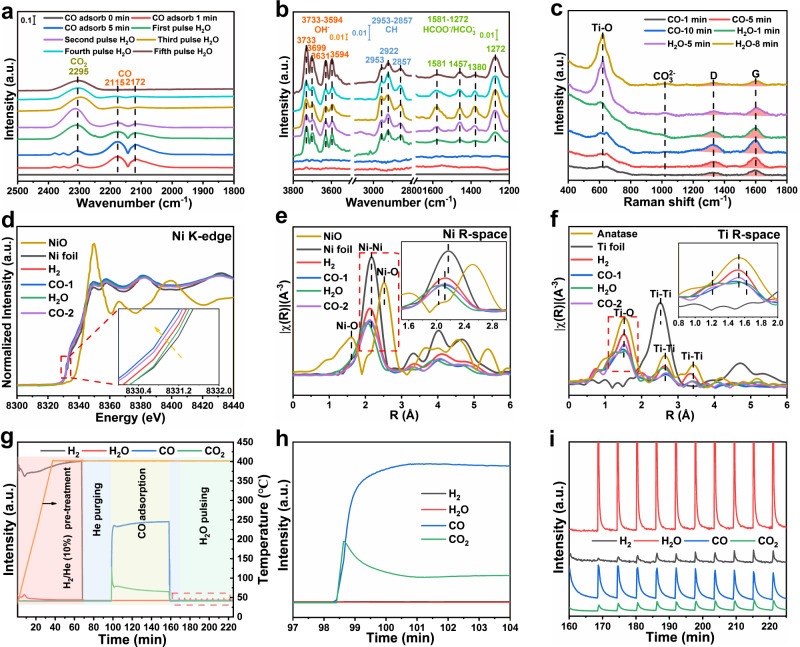
In situ characterizations and reaction mechanism of CO reforming. **a**, **b** In situ DRIFT spectra, **c** in situ Raman spectra, **d** in situ normalized XANES spectra, **e** in situ Fourier-transform EXAFS spectra at Ni K-edge in R-space, and **f** Ti K-edge in R-space of CO adsorption and then H_2_O adsorption on 0.5RhNi/TiO_2_ catalyst at 400 °C (H_2_, CO-1, H_2_O, and CO-2 denote the spectra after successive H_2_ pretreatment, CO adsorption, H_2_O adsorption, and CO adsorption again for 20 min on 0.5RhNi/TiO_2_ catalyst, respectively). **g** Mass spectral analysis for CO and H_2_O pulse over 0.5RhNi/TiO_2_ catalyst and **h**, **i** corresponding local magnification regions in **g**.

Based on the catalytic evaluations and kinetic studies, the key steps determining H_2_ generation is the transformation of intermediate products (CO and CH_*x*_). A series of in situ DRIFT measurements were carried out to investigate the influence of SBMSI on CO and CH_*x*_ transformation. As 0.5RhNi/TiO_2_ is exposed to CO at 400 °C, CO_2_ adsorption bands within 2270–2389 cm^−1^ appear^34,35^, whose intensity enhances gradually along with time, indicating the occurrence of CO disproportionation on the catalyst surface to produce CO_2_ and C species (Supplementary Fig. 34 and Supplementary Note 9). Subsequently, a switching to H_2_O pulse leads to the decline of CO signal, accompanied by the formation of Ti^3+^−OH and Ti^4+^−OH species (3733–3594 cm^−1^); and several absorption bands including formate species (2953–2857, 1581, and 1380 cm^−1^) and bidentate hydrogen-carbonates species (1457 and 1272 cm^−1^) are observed (Fig. 4a, b)^1,9,36,37^. The peak intensity of formate enhances firstly and then declines with the pulse of water, indicating that such species is an important reaction intermediate. To further identify the generation path for formate intermediate, another H_2_O pulse test is implemented again after purging the surface CO and CO_2_ by He (Supplementary Fig. 35). The analogous formate intermediate is detected, indicating that formate is derived from the further transformation of carbon species produced by CO disproportionation. In situ Raman was further used to verify this issue (Fig. 4c), in which two remarkable bands at 1330 and 1609 cm^−1^ ascribed to D and G bands of carbon species were observed after purging CO^18,38,39^. Afterwards, the band intensity of carbon species declines, and the Ti−O signal increases for 0.5RhNi/TiO_2_ catalyst with the introduction of saturated water vapor. Furthermore, when CO and saturated water vapor are introduced together, very few carbon species are detected, indicating that accumulation and consumption of carbon maintain a balance under WGS reaction conditions (Supplementary Fig. 36).

Furthermore, the TPSR-mass spectral analysis is carried out. Firstly, the 0.5RhNi/TiO_2_ catalyst was pretreated in H_2_ at 400 °C for 30 min followed by He flushing for 30 min. Then, the gas was switched to CO, He, and saturated water vapor in turn to monitor the gas signals (Fig. 4g–i). The formation of CO_2_ was detected synchronously with the introduction of CO, indicating the occurrence of CO disproportionation to generate CO_2_ and C species (Fig. 4h), in accordance with the results of in situ DRIFT (Fig. 4a and Supplementary Figs. 34 and 35a) and in situ Raman spectra (Fig. 4c). Subsequently, He flushing followed by a saturated water vapor pulse over the catalyst gave rise to the generation of H_2_ and CO_2_ (Fig. 4i). Such phenomenon demonstrates that H_2_O dissociation occurs on oxygen vacancy of TiO_2_ to generate active hydroxyl/oxygen groups, which further react with surface carbon species to give formate intermediate; subsequently, formate species undergoes decomposition to produce CO_2_ and H_2_. The results above demonstrate that the WGS reaction obeys an associative mechanism on the surface of 0.5RhNi/TiO_2_.

For Ni/TiO_2_ catalyst, a similar associative reaction mechanism was also verified (Supplementary Figs. 38a and 39), except a lower intensity of formate intermediate, corresponding to its lower CO conversion (Supplementary Note 11 and Supplementary Note 12). For Rh/Ni catalyst without SBMSI, CO disproportionation process occurs; however, no associative reaction intermediate is detected after purging H_2_O pulse as proved by in situ DRIFT and in situ Raman results (Supplementary Fig. 40 and Supplementary Note 13). Furthermore, the mass spectral analysis confirms that H_2_O experiences dissociation over Rh/Ni catalyst to produce H_2_ and active O species, and then O species reacts with CO or C species to form CO_2_ (Supplementary Fig. 37 and Supplementary Note 10), which accords with redox mechanism^20,40–42^. Moreover, in situ Raman spectra also confirm the existence of carbonate species (1000−1200 cm^−1^) on Rh/Ni and 0.5RhNi/TiO_2_ catalyst. The more significant intensity in the former case originates from a stronger binding of CO_2_ (Fig. 4c and Supplementary Fig. 40c), which is consistent with the test results. The impact of CO_2_ was discussed in detail based on in situ DRIFT, in situ Raman and *Quasi*-in situ XPS in supporting information (Supplementary Figs. 41–44 and Supplementary Notes 14–16), where 0.5RhNi/TiO_2_ with SBMSI shows the strongest CO_2_ resistance. For Rh/TiO_2_ catalyst, CO disproportionation does not occur along with fewer reaction intermediates after H_2_O pulse (Supplementary Figs. 38c and 45, and Supplementary Note 17), which is associated with its poor WGS reaction activity.

In addition, we also carried out in situ DRIFT investigations for CH_4_ steam reforming on these catalysts to imitate the transformation of CH_*x*_ species. After purging CH_4_, an obvious peak appears at 1542 cm^−1^ assigned to bidentate formate intermediate on 0.5RhNi/TiO_2_, Ni/TiO_2_ and Rh/Ni samples (Supplementary Fig. 46). When introducing saturated water vapor into the reaction cell at 400 °C, formate intermediate declines accompanied with the formation of CO_2_. Both the consumption rate of formate intermediate and the band intensity of *δ*(CH_*x*_) at 1542 and 1302 cm^−1^ decrease in the following order: 0.5RhNi/TiO_2_ > Ni/TiO_2_ > Rh/Ni (Supplementary Fig. 47 and Supplementary Note 18), in accordance with the test results (Fig. 1b, e). However, no obvious intermediate is found in the case of Rh/TiO_2_ due to its poor catalytic performance (Supplementary Fig. 46h). Furthermore, no obvious carbon species is observed in in situ Raman spectra for 0.5RhNi/TiO_2_ and Rh/Ni catalysts, ruling out the complete dehydrogenation of CH_*x*_ in reforming processes (Supplementary Fig. 48 and Supplementary Notes 19), since the most stable CH fragment can react with hydroxy group or active oxygen to produce formate intermediate (CH_*x*_ → CH + H_*x*–1_ → HCOO^−^)^37,43–45^. After switching to a saturated water vapor, carbonate species (1000−1200 cm^−1^) resulting from CO_2_ behaves a stronger binding onto Rh/Ni than 0.5RhNi/TiO_2_ catalyst (Supplementary Fig. 48), similar to the CO reforming process. Owing to the similar O−C−O structure of formate to CO_2_ or carbonate, the lower conversion of CH_*x*_ on Rh/Ni is possibly associated with the stronger binding of formate to Ni sites, which inhibits its further transformation. Therefore, the 0.5RhNi/TiO_2_ catalyst with unique coordination and electronic structure resulting from SBMSI reduces the binding ability of species with analogous COO^−^ structure (CO_2_, carbonate, or formate), and thus promotes the generation and transformation of formate intermediate, accounting for its excellent reforming activity of CO and CH_*x*_.

In situ XAFS measurements were performed to track the dynamic evolution of coordination structure and electronic state of bimetal-support interface sites during steam reforming process. For the 0.5RhNi/TiO_2_ sample, when CO is introduced into the reaction system (CO-1), the absorption edge of Ni shifts to lower energy (Fig. 4d), accompanied with a decrease in the coordination number of Ni−Ni bond (Fig. 4e, Supplementary Fig. 49b and Supplementary Table 6). The corresponding variations in Ti K-edge XAFS spectra are also observed, where the A_2_ peak shifts to lower energy (Supplementary Fig. 49c) with a decline in the coordination number of Ti−O bond (Fig. 4f). This is attributed to the catalyst reconstruction resulting from CO disproportionation, in which the generated carbon species fixed to Ni sites reacts with active oxygen species offered by TiO_2_ at interface sites. Once the reaction atmosphere is switched to a saturated water vapor (H_2_O), the absorption edge of Ni moves toward higher energy and Ni−Ni bond length becomes shorter (Fig. 4d, e), due to the formation of formate intermediate on the catalyst surface. In addition, the intensity of A_2_ peak in Ti K-edge XANES spectra (Supplementary Fig. 49c) declines and the Ti−O bond splits upon exposure to H_2_O vapor (Fig. 4f). This is ascribed to the quenching of oxygen vacancy in TiO_2_ by active hydroxy species (from Ti^3+^–O_*v*_ to Ti^4+^–OH^−^), along with a structure reconfiguration. After purging CO again (CO-2), the catalyst structure is restored to its original state and a reaction cycle is completed. The results substantiate that the bimetal-support interfacial sites participate in the steam reforming processes, accompanied by the interface reconstruction in the case of 0.5RhNi/TiO_2_ catalyst. In contrast, the metal sites on Rh/Ni catalyst play a dual role toward activation adsorption of both water molecule and reactants, corresponding to its poor catalytic properties. A detailed discussion on in situ XAFS measurements was offered in supplementary materials (Supplementary Fig. 50, Supplementary Table 7 and Supplementary Note 20).

**Fig. 5:**
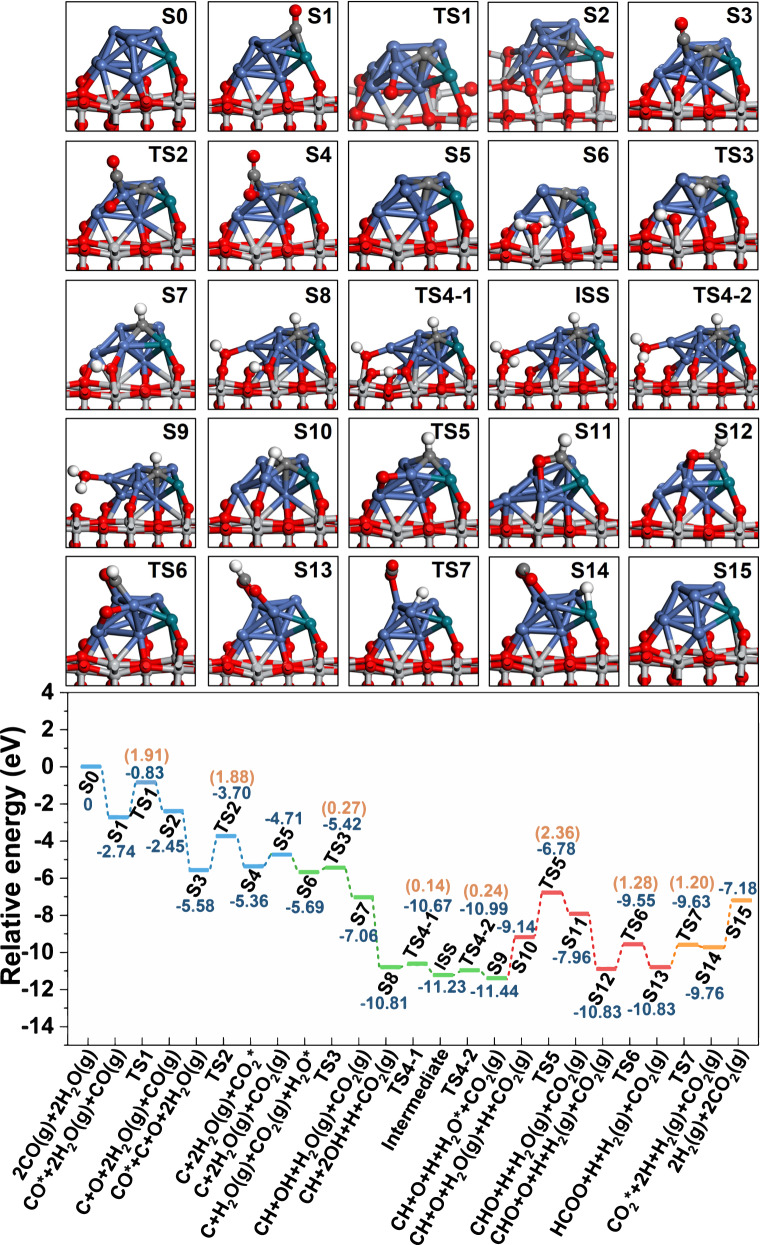
DFT calculation and schematic illustration. Reaction mechanism of stream reforming of CO on the surface of Rh_1_Ni_7_/TiO_2–*x*_. ‘S’ denotes a stable sorption state; ‘TS’ denotes a transition state; ‘ISS’ denotes an intermedia stable state. (Blue, green, red, and orange dotted lines indicate CO disproportionation, H_2_O dissociation, formate generation, and CO_2_ desorption, respectively; blue and orange numbers represent adsorption energy and reaction energy barrier, respectively).

Furthermore, we carried out first-principle calculations to further understand the roles of bimetal-support interfacial sites in ethanol conversion and steam reforming processes of CO and CH_*x*_, and the Rh_1_Ni_7_/TiO_2-*x*_ model systems was built (Supplementary Fig. 51 and Supplementary Note 21). Based on the Bader charge analysis results, compared with the Ni_8_/TiO_2*-x*_ (Supplementary Figs. 52a, c), the Rh_1_Ni_7_/TiO_2*-x*_ system (Supplementary Figs. 52b, d) with SBMSI weakens the charge transfer from the oxygen vacancy on TiO_2*-x*_ support to the Rh_1_Ni_7_ bimetallic interface (Supplementary Fig. 52e), which is consistent with the electronic structure characterization (Fig. 3d, g). For the ESR reaction mechanism, the reaction energy barriers for ethanol dehydrogenation and acetaldehyde decomposition were studied through DFT calculations. Based on the calculation results (Supplementary Figs. 53−62, Supplementary Table 8 and Supplementary Note 22), the optimal ethanol dehydrogenation path in Ni_8_/TiO_2−*x*_ and Rh_1_Ni_7_/TiO_2−*x*_ systems follows CH_3_CH_2_OH → CH_3_CH_2_O* → CH_3_CHO* → CH_3_CO*, and then CH_3_CO* undergoes C−C bond breaking to produce CH_3_* and CO, which is consistent with the product distribution and *operando* characterizations (DRIFTS spectra and pulse experiment). Compared with Ni_8_/TiO_2−*x*_, the energy barriers for ethanol dehydrogenation and C−C bond cleavage decrease from 1.65 and 1.31 eV to 1.03 and 1.17 eV on Rh_1_Ni_7_/TiO_2−*x*_, respectively. For the reaction mechanism of stream reforming of CO (Supplementary Figs. 63–72, Supplementary Table 8 and Supplementary Note 23), the potential energy profiles over Rh_1_Ni_7_/TiO_2*-x*_ and Ni_8_/TiO_2-*x*_ catalysts are similar and shown in Fig. 5 and Supplementary Fig. 73, which mainly consists of four steps. Firstly, CO molecule undergoes activation adsorption at the hollow sites (adjacent to two Ni atoms and one Rh atom for Rh_1_Ni_7_/TiO_2*-x*_; adjacent to three Ni atoms for Ni_8_/TiO_2*-x*_), and then dissociates to form C and O, followed by disproportionation reaction with another CO molecule to generate CO_2_ and C species (blue dotted lines in Fig. 5 and Supplementary Fig. 73); subsequently, H_2_O molecule experiences activation adsorption at interfacial oxygen vacancy, which dissociates to active hydroxyl and oxygen species (green dotted lines in Fig. 5 and Supplementary Fig. 73); afterwards, the hydrogen from H_2_O dissociation binds to the carbon species to generate CH fragment, which is then attacked by active oxygen species to produce the HCOO^−^ intermediate (red dotted lines in Fig. 5 and Supplementary Fig. 73); finally, formate undergoes decomposition to produce CO_2_ and H_2_ (orange lines in Fig. 5 and Supplementary Fig. 73). According to the calculation results, the formation of HCOO^−^ intermediate is the rate-determining step with an energy barrier of 2.36 and 2.79 eV on Rh_1_Ni_7_/TiO_2*-x*_ and Ni_8_/TiO_2-*x*_ catalysts, respectively. In addition, for the steam reforming processes of CH_*x*_, the successive dehydrogenation of methyl occurs firstly on the surface of Ni and RhNi bimetal sites to generate CH fragment (Supplementary Figs. 74, 75 and Supplementary Note 24), followed by a similar process mentioned above for the transform of CH to HCOO^−^ (Supplementary Figs. 67 and 71). In contrast, the formate formation from C/CH fragment shows an energy barrier of 2.36 and 2.79 eV on Rh_1_Ni_7_/TiO_2−*x*_ and Ni_8_/TiO_2−*x*_ catalysts, respectively, much larger than that of ethanol dehydrogenation (1.03 and 1.65 eV) and acetaldehyde decomposition (1.17 and 1.31 eV), indicating that the transformation of CO and CH_*x*_ is the crucial step, in accordance with the experimental results. Especially, the lower reaction energy barriers on Rh_1_Ni_7_/TiO_2−*x*_ relative to Ni_8_/TiO_2−*x*_ verify that the ESR reaction is boosted at the bimetal-support interface sites, in well agreement with the catalytic evaluations.

In summary, we report a RhNi/TiO_2_ catalytic system with well-defined SBMSI towards ESR reaction. The obtained 0.5RhNi/TiO_2_ catalyst gives exceptional hydrogen production (H_2_ yield: 61.7%) and catalysis stability (300 h) at a relatively low temperature (400 °C). The microscopic fine-structure of RhNi/TiO_2_ was studied by STEM, CO-DRIFT, and XAFS, in which the RhNi bimetallic nanoparticle with a reversible TiO_2_ coating exhibited a multiple electron transfer pathway at the interfacial active sites (Rh-Ni^*δ*−^-O_*v*_-Ti^3+^). A comprehensive investigation including in situ spectroscopic characterizations, *operando* pulse experiments, kinetics studies, and DFT calculations substantiates that the ESR reaction in the presence of 0.5RhNi/TiO_2_ catalyst follows a CO/CH_*x*_-mediated reforming process rather than the acetate path. This bimetal-support interface (Rh-Ni^*δ*−^-O_*v*_-Ti^3+^) plays a decisive role in the steam reforming of CO and CH_*x*_ originating from ethanol dissociation, which is involved in the rate-determining step of ESR reaction. The modulated geometric and electronic structure of interfacial active sites resulting from SBMSI reduce the binding ability of species with COO^−^ structure (CO_2_, carbonate, or formate). This facilitates the generation and transformation of formate intermediate from CO/CH_*x*_ reforming processes, which ensures a prominent hydrogen production rate and catalytic stability. The well-defined SBMSI demonstrated in this work can be extended to other structure-sensitive reactions involving multiple reaction substrates.

## Methods

### Chemicals and materials

Chemical reagents, including Ni(NO_3_)_2_·6H_2_O, Al(NO_3_)_3_·9H_2_O, tetrabutyl titanate, anatase, and urea were bought from Aladdin chemical reagent company. RhCl_3_·3H_2_O was purchased from Beijing HWRK CHEM; absolute ethanol was purchased from Tianjin DaMao chemical reagent factory. The above chemical reagents were used directly without further purification. Quartz sand (SiO_2_, 40−60 mesh) was purchased from Tianjin Guangfu fine chemical research institute, and washed by using concentrated HCl prior to use. Deionized (DI) water with a resistivity of 18.2 MΩ cm was used in all experimental processes.

### Synthesis of catalysts

RhNiTi-LDHs (RhNiTi-layered double hydroxides) precursors were prepared *via* urea homogeneous precipitation method. Briefly, tetrabutyl titanate (0.013 mol L^−1^), Ni(NO_3_)_2_·6H_2_O (0.040 mol L^−1^), and urea (0.500 mol L^−1^) were dissolved in deionized water (150 mL) with vigorous stirring for 8 h at refluxing temperature (95 °C). After 40 min of reaction, a certain amount of RhCl_3_·3H_2_O aqueous solution (7.6 mg mL^−1^) was slowly dripped into above solution. The resulting precipitate was filtered, washed thoroughly with deionized water until neutral, followed by drying at 80 °C for 12 h to obtain RhNiTi-LDHs. Subsequently, the RhNiTi-MMO (RhNiTi-mixed metal oxide) samples were obtained *via* calcining the RhNiTi-LDH precursors at 500 °C in air for 4 h followed by cooling to room temperature. Prior to the catalytic reaction, the RhNiTi-MMO samples were treated in a mixture gas (H_2_/N_2_ = 1/9; flow rate: 50 mL min^−1^) at 400 °C for 2 h to obtain RhNi/TiO_2_ catalysts. The resulting samples with various Rh content are denoted as *x*RhNi/TiO_2_ (*x* = 0.1, 0.3, 0.5, 0.8, or 1.0), where the *x* represents the theoretical mass percentage of Rh (e.g., 0.5RhNi/TiO_2_ indicates a 0.5 *wt*.% Rh in this sample). According to the same method described above, the Rh/Ni and Ni/TiO_2_ catalysts were also prepared without the introduction of Ti and Rh, respectively. In addition, the Rh/TiO_2_ sample was prepared by traditional impregnation method with 0.5 *wt*.% Rh loading by using anatase as support. The calcination and reduction conditions are consistent with those of *x*RhNi/TiO_2_ samples. Inductively coupled plasma atomic emission spectroscopy (ICP-AES) is used to determine the chemical composition of these samples, which is close to the feeding ratio (Supplementary Table 1).

### Catalyst characterizations

The powder XRD (X-ray diffraction) patterns are recorded on a Rigaku XRD-6000 diffractometer using a nickel-filtered Cu K_*α*_ radiation source (*λ* = 0.15418 nm) at 40 kV and 30 mA with a scanning rate of 8° min^−1^ and a 2*θ* angle ranging from 5° to 80°. Crystalline phases are identified by comparison with the reference data from International Center for Diffraction Data (ICDD) files. The chemical composition of various samples is measured by ICP-AES (Shimadzu ICPS-7500). The specific surface area and pore structure parameters of samples are obtained from N_2_ adsorption and desorption isotherms by using a Quantachrome Auosorb-1C-VP analyzer based on the Brunauer-Emmett-Teller (BET) and Barret-Joyner-Halenda (BJH) models. Sample morphology and structure are characterized by scanning electron microscopy (SEM, Zeiss Supra 55) with applied voltage of 20 kV and transmission electron microscopy (TEM, JEOL JEM-2010) with accelerating voltage of 200 kV. Aberration-corrected high-angle annular dark-field scanning transmission electron microscopy (AC-HAADF-STEM) and element energy-dispersive spectroscopy (EDS) mapping images are conducted on a JEOL JEM-ARM200F equipment. The chemical states of sample surface are investigated by using Thermo VG Escalab 250 X-ray photoelectron spectroscopy (XPS) with Al K_*α*_ as a radiation source at 300 W under UHV (2 × 10^−9^ Torr). The sample after treatment was transferred into sample rod in glove box with N_2_ atmosphere, and sample charging effects are eliminated by correcting the observed spectra with C 1 *s* binding energy value of 284.8 eV.

Carbon dioxide temperature programmed desorption (CO_2_-TPD), hydrogen temperature programmed desorption (H_2_-TPD), and hydrogen temperature programmed reduction (H_2_-TPR) are carried out on a Micromeritics Chemi-Sorb 2920 instrument equipped with a thermal conductivity detector (TCD). For the CO_2_-TPD, the sample (0.08 g) was firstly pretreated at 400 °C in a H_2_ atmosphere for 1 h, followed by purging with He for 0.5 h, and then the temperature was decreased to 50 °C. Subsequently, 5% CO_2_ was introduced with He as carrier gas until saturation adsorption. Then, pure He was purged, along with the increase of temperature from 50 to 700 °C (rate: 10 °C min^−1^) for the collection of signals. For the H_2_-TPD, the sample (0.08 g) was firstly pretreated at 400 °C in a H_2_ atmosphere for 1 h, followed by purging with Ar for 0.5 h, and then the temperature was decreased to 50 °C. Afterwards, a 5% H_2_ was introduced with Ar as carrier gas until a saturation adsorption. The gas was switched to pure Ar, along with the increase of temperature from 50 to 700 °C (rate: 10 °C min^−1^) for signal collection. For the H_2_-TPR, the sample (0.1 g) was firstly pretreated at 250 °C in a Ar atmosphere for 1 h, followed by the decrease of temperature to 50 °C. Then, the gas was switched to a 5% H_2_ with Ar as carrier gas. The temperature was increased from 50 to 600 °C with a rate of 10 °C min^−1^, and meanwhile the H_2_ consumption signal was recorded.

In situ diffuse reflectance infrared fourier-transform spectra (in situ DRIFTS) are recorded on a VERTEX 70 BRUKER spectrometer equipped with CaF windows and a mercury-cadmium-telluride (MCT) detector, with a resolution of 4 cm^−1^ using 100 scans. The catalyst was filled into an in situ reaction cell and pressed into a flat surface. Firstly, the sample was pretreated at 400 °C in a 10% H_2_ flow for 1 h followed by purging He for 0.5 h at 400 °C. Prior to the test, the reference baseline was collected; then a switching to CO/CH_4_ was performed and the diffuse signals were collected at different time points. Furthermore, H_2_O/H_2_O + CO_2_ was introduced by a pulse with N_2_ as carrier gas *via* quantitative loop (1 μL) for signal collection. CO-DRIFT adsorption is carried out at ambiance temperature (20 °C) with CO concentration of 0.5%. For the five-cycle processes of H_2_ reduction-O_2_ oxidation with 10%H_2_/N_2_ and air atmosphere, respectively, the reduction and oxidation were performed for 1.0 h at 400 °C. In situ Raman spectra were recorded on a Renishaw in Via-Reflexm equipped with a laser (532 nm). The catalyst was filled into an in situ reaction cell and pretreated in a 10% H_2_ flow at 400 °C for 1 h followed by purging He at 400 °C for 0.5 h. Afterwards, the Raman signals were collected continually during the adsorption of CO and H_2_O/CO_2_. In situ x-ray absorption fine-structure spectra (in situ XAFS) at Ni K-edge and Ti K-edge are performed at the beamline 1W1B of the Beijing Synchrotron Radiation Facility (BSRF), Institute of High Energy Physics (IHEP), Chinese Academy of Sciences (CAS). The 50 mg of different catalysts were filled into an in situ reaction cell and pretreated in a 10% H_2_ flow at 400 °C for 1 h followed by purging He at 400 °C for 0.5 h. Afterwards, the signals were collected during the adsorption of CO and H_2_O. Mass spectral analysis of CO and H_2_O pulsing tests are carried out by Micromeritics Chemi-Sorb 2920 instrument equipped with a mass spectrometry detector (MS).

### Catalytic evaluations for ESR

Catalytic performances of as-synthesized samples toward steam reforming of ethanol (ESR) reaction are studied in a fix-bed reactor with a stainless steel tube (interior diameter: 10 mm) at atmospheric pressure. Prior to the catalytic reaction, 0.15 g of catalyst mixed with quartz sand (40−60 mesh, 1.50 g) was pretreated in a gaseous mixture of H_2_ and N_2_ (1:9, v/v; a total gas flow of 50.0 mL min^−1^) at 400 °C for 2 h, and then cooled to reaction temperature (350 °C and 400 °C) in N_2_ atmosphere. The water and ethanol mixture with steam/carbon (S/C) ratio is 3 was injected into the reaction system by using a HPLC pump at a rate of 0.060 mL min^−1^. The reactants were evaporated in a preheater (170 °C) with a heating belt to avoid product condensation, followed by mixing with nitrogen gas (50.0 mL min^−1^). The temperature of the whole installation was modulated by a K-type thermocouple. When the reaction was carried out at 350 and 400 °C for 1.5 h, the products were analyzed online by using a gas chromatograph (Shimadzu, GC-17A) with both FID and TCD detectors equipped with TDX-01 and HP-PLOT/Q columns, respectively. Ethanol conversion (*X*), product yield (*Y*), and hydrogen selecticity (${S}_{{{{{{{\rm{H}}}}}}}_{2}}$) are calculated as following equations.

1 $${X}_{{{{{{\rm{EtOH}}}}}}}=\frac{{F}_{{{{{{\rm{EtOH}}}}}},{{{{{\rm{in}}}}}}}-{F}_{{{{{{\rm{EtOH}}}}}},{{{{{\rm{out}}}}}}}}{{F}_{{{{{{\rm{EtOH}}}}}},{{{{{\rm{in}}}}}}}}\times 100\%$$

2 $${Y}_{{{{{{{\rm{H}}}}}}}_{2}}=\frac{{F}_{{{{{{{\rm{H}}}}}}}_{2}}}{6\times {F}_{{{{{{\rm{EtOH}}}}}},{{{{{\rm{in}}}}}}}}\times 100\%$$

3 $${Y}_{{C}_{i}}=\frac{{F}_{{C}_{i}\times j}}{2\times {F}_{{{{{{\rm{EtOH}}}}}},{{{{{\rm{in}}}}}}}}\times 100\%$$

4 $${S}_{{{{{{{\rm{H}}}}}}}_{2}}=\frac{{F}_{{{{{{{\rm{H}}}}}}}_{2}}}{{F}_{{{{{{{\rm{H}}}}}}}_{2}}+2\times {F}_{{{{{{{\rm{CH}}}}}}}_{4}}+2\times {{{\mbox{F}}}}_{{{{\mbox{CH}}}}_{3}{{\mbox{CHO}}}}}\times 100\%$$

*F*_EtOH,in/out_ is the molar flow rate of ethanol at the inlet/outlet of the reactor, respectively. ${F}_{{{{{{{\rm{H}}}}}}}_{2}}$ and ${F}_{{C}_{i}}$ denote the molar flow rate of H_2_ and C-containing product at the reactor outlet, respectively, where the *j* indicates the number of carbon atoms in the latter. The molar flow of acetaldehyde, ethylene, and methane is determined based on FID results. The molar flow of other gas products (H_2_, CO and CO_2_) in the effluent are measured by TCD results, which are calculated based on Eqs. (5) and (6):

5 $${V}_{j}=V\times {A}_{j}\times \frac{{y}_{j}^{{std}}}{{A}_{j}^{{std}}}$$

6 $${PV}=n{{{{{\rm{R}}}}}}T$$

where *T*, *P*, R, *n*, and *V* are the room temperature (K), pressure (Pa), molar gas constant (8.314 J mol^−1^ K^−1^), molar number, and total volumetric flow of the gas outlet, respectively. *A*_*j*_ is the peak area of component *j* obtained from TCD signal. *A*^std^_j_ and *y*^std^_j_ are the peak area and the molar fraction of component *j* in the standard gas mixture, respectively.

### Reaction dynamics analysis

For the measurement of reaction rate of ethanol and acetaldehyde, CO, and CH_4_ steam reforming reaction, the quartz sand (SiO_2_, 50−300 mg) and the catalyst sample (5−30 mg) are sieved separately (40−60 mesh) and then physically mixed together before installed into the reactor tube. Reaction conditions for kinetics studies over 0.5RhNi/TiO_2_, Ni/TiO_2_, Rh/Ni, and Rh/TiO_2_ catalysts are as follows: liquid feed of ethanol and acetaldehyde at 0.040 mL min^−1^, liquid feed of H_2_O at 0.032 mL min^−1^, gas flow rate of CO at 50 mL min^−1^ and CH_4_ at 10.0 mL min^−1^, CO_2_/H_2_ at 10.0 mL min^−1^, N_2_ carrier at 50 mL min^−1^, reaction temperature: 240−400 °C, time on stream: 0.5 h. For the determination of reaction order of ethanol dehydrogenation and acetaldehyde decomposition, the initial partial pressure of ethanol and acetaldehyde are 7−39 kPa and 4−15 kPa, respectively. For the determination of the reaction order of CH_4_ steam reforming, the initial partial pressure of CH_4_ is 4−12 kPa. For the determination of the reaction order of CO steam reforming, the initial partial pressure of H_2_O, CO, CO_2_, and H_2_ is tuned within 20−40 kPa, 4−17 kPa, 3−13 kPa, and 4−23 kPa, respectively.

### Computational details

Density functional theory (DFT) calculations are performed in Vienna ab initio simulation package (VASP) with the generalized gradient approximation (GGA) using the Perdew-Burke-Ernzerhof (PBE) functional^46^. The projected augmented wave (PAW) potentials are used to describe the ionic cores, and valence electrons are also considered using a plane wave basis set with a kinetic energy cutoff of 400 eV^47^. Geometry optimizations are performed with the force convergency smaller than 0.05 eV A^−1^, where the same convergency is applied for the location of transition states by the constrained optimizations. The original bulk structure is optimized before the construction of surfaces with the Monkhorst-Pack k-point of 3 × 3 × 1. The TiO_2_(110) surface with 24 Ti and 48 O atoms is applied with half of the atoms at the bottom fixed in all the calculations. A Ni_8_ cluster with 8 Ni atoms is placed on the TiO_2_(110) surface to describe the interface of Ni/TiO_2_. According to the STEM results, one Ni atom is then replaced by one Rh atom on Ni_8_ surface to build the RhNi/TiO_2_ interface structure. A Monkhorst-Pack k-point 3 × 3 × 1 is applied for all the calculations on surfaces. In addition, the effect from the Hubbard U corrections is considered beyond the accuracy of DFT calculations of GGA, where *U* value (employed as U-J) of 3.5 is applied for Ti, Ni, and Rh.

Transition state (TS) searches are performed at the same theoretical level with the CI-NEB method. All the models are the most stable structure obtained through optimization and screening. The formation energy (*FE*_O*v*_) is used in analyzing oxygen vacancy formation (O_*v*_), defined as

7 $${{FE}}_{{Ov}}={E}_{{{{{{\rm{vo}}}}}}-{{{{{\rm{slab}}}}}}}+{E}_{{{{{{\rm{o}}}}}}-{{{{{\rm{gas}}}}}}}-{E}_{{{{{{\rm{slab}}}}}}}$$

where *E*_vo-slab_, *E*_o-gas_, and *E*_slab_ are the total energies for the oxygen vacancy slab, the oxygen atom in the gas phase, and the clean surface, respectively.

The adsorption energy (*E*_ads_) is calculated as

8 $${E}_{{{{{{\rm{ads}}}}}}}={E}_{{{{{{\rm{total}}}}}}}-\left({E}_{{{{{{\rm{slab}}}}}}}+{E}_{{{{{{\rm{g}}}}}}}\right)$$

where *E*_total_ is the total energy after adsorption; *E*_slab_ is the energy of the clean slab before adsorption, and *E*_g_ is the energy of the free adsorbate in the gas phase.

The energy barrier (*E*_a_) is obtained from the electronic energy difference between the transition state (*E*_TS_) and its corresponding initial state (*E*_IS_), which is calculated by

9 $${E}_{{{{{{\rm{a}}}}}}}={E}_{{{{{{\rm{TS}}}}}}}-{E}_{{{{{{\rm{IS}}}}}}}$$

In this work, a Ni_7_Rh_1_/TiO_2–*x*_ model is applied for calculation, in which Ni_7_Rh_1_ cluster is supported on the TiO_2_(110) surface with oxygen vacancy. Before the calculations, the model of Ni_7_Rh_1_/TiO_2–*x*_ is optimized. The lattice parameters of TiO_2_ support are: *a* = *b* = 3.79 Å, *c* = 9.56 Å, *α* = *β* = *γ* = 90° (body-centered tetragonal) with a p(2 × 2) supercell.

## Supplementary information

Supplementary Information

Peer Review File

## Data Availability

The primary data that support the plots within this paper and other finding of this study are available from the corresponding author on reasonable request. Source data are provided with this paper.

## Source data

Source data

## References

[1] Larmier K (2017). CO_2_-to-methanol hydrogenation on zirconia-supported copper nanoparticles: reaction intermediates and the role of the metal–support interface. Angew. Chem. Int. Ed..

[2] Parastaev A (2020). Boosting CO_2_ hydrogenation via size-dependent metal–support interactions in cobalt/ceria-based catalysts. Nat. Catal..

[3] Lin L (2017). Low-temperature hydrogen production from water and methanol using Pt/α-MoC catalysts. Nature.

[4] Frey H, Beck A, Huang X, van Bokhoven JA, Willinger MG (2022). Dynamic interplay between metal nanoparticles and oxide support under redox conditions. Science.

[5] Hernandez Mejia C, van Deelen TW, de Jong KP (2018). Activity enhancement of cobalt catalysts by tuning metal-support interactions. Nat. Commun..

[6] Li S (2017). Tuning the selectivity of catalytic carbon dioxide hydrogenation over iridium/cerium oxide catalysts with a strong metal–support interaction. Angew. Chem. Int. Ed..

[7] Zhang Y (2020). Ru/TiO_2_ Catalysts with size-dependent metal/support interaction for tunable reactivity in fischer–tropsch synthesis. ACS Catal..

[8] Xin H (2022). Overturning CO_2_ hydrogenation selectivity with high activity via reaction-induced strong metal–support interactions. J. Am. Chem. Soc..

[9] Matsubu JC (2017). Adsorbate-mediated strong metal–support interactions in oxide-supported Rh catalysts. Nat. Chem..

[10] Gesesse GD (2020). A soft-chemistry assisted strong metal–support interaction on a designed plasmonic core–shell photocatalyst for enhanced photocatalytic hydrogen production. Nanoscale.

[11] Zhang J (2019). Wet-chemistry strong metal–support interactions in titania-supported Au catalysts. J. Am. Chem. Soc..

[12] Li X (2020). Controlling CO_2_ hydrogenation selectivity by metal-supported electron transfer. Angew. Chem. Int. Ed..

[13] Tang M (2021). Facet-dependent oxidative strong metal-support interactions of palladium–TiO_2_ determined by in situ transmission electron microscopy. Angew. Chem. Int. Ed..

[14] Xie C, Niu Z, Kim D, Li M, Yang P (2020). Surface and interface control in nanoparticle catalysis. Chem. Rev..

[15] Graciani J (2014). Highly active copper-ceria and copper-ceria-titania catalysts for methanol synthesis from CO_2_. Science.

[16] Zhang Y (2020). Tuning reactivity of Fischer–Tropsch synthesis by regulating TiO_x_ overlayer over Ru/TiO_2_ nanocatalysts. Nat. Commun..

[17] Chen L (2021). Insights into the mechanism of methanol steam reforming tandem reaction over CeO_2_ supported single-site catalysts. J. Am. Chem. Soc..

[18] Wu Y (2021). Role of Fe species of Ni-based catalysts for efficient low-temperature ethanol steam reforming. JACS Au.

[19] Yu K (2012). Non-syngas direct steam reforming of methanol to hydrogen and carbon dioxide at low temperature. Nat. Commun..

[20] Zhang X (2021). A stable low-temperature H_2_-production catalyst by crowding Pt on α-MoC. Nature.

[21] Li Y (2021). Dynamic structure of active sites in ceria-supported Pt catalysts for the water gas shift reaction. Nat. Commun..

[22] Zhang Z-S (2020). Intrinsically active surface in a Pt/γ-Mo_2_N catalyst for the water–gas shift reaction: molybdenum nitride or molybdenum oxide?. J. Am. Chem. Soc..

[23] Chen S, Pei C, Gong J (2019). Insights into interface engineering in steam reforming reactions for hydrogen production. Energ. Environ. Sci..

[24] Zanchet D, Santos JBO, Damyanova S, Gallo JMR, Bueno JMC (2015). Toward understanding metal-catalyzed ethanol reforming. ACS Catal..

[25] Mattos LV, Jacobs G, Davis BH, Noronha FB (2012). Production of hydrogen from ethanol: review of reaction mechanism and catalyst deactivation. Chem. Rev..

[26] Artrith N, Lin Z, Chen JG (2020). Predicting the activity and selectivity of bimetallic metal catalysts for ethanol reforming using machine learning. ACS Catal..

[27] Deluga GA, Salge JR, Schmidt LD, Verykios XE (2004). Renewable hydrogen from ethanol by autothermal reforming. Science.

[28] Crowley S, Castaldi MJ (2016). Mechanistic insights into catalytic ethanol steam reforming using isotope-labeled reactants. Angew. Chem. Int. Ed..

[29] Tian H (2021). Tunable metal-oxide interaction with balanced Ni^0^/Ni^2+^ sites of Ni_x_Mg_1−x_O for ethanol steam reforming. Appl. Catal. B-Environ..

[30] Tauster SJ, Fung SC, Garten RL (1978). Strong metal-support interactions. Group 8 noble metals supported on titanium dioxide. J. Am. Chem. Soc..

[31] Beck A (2020). The dynamics of overlayer formation on catalyst nanoparticles and strong metal-support interaction. Nat. Commun..

[32] Han B (2020). Strong metal–support interactions between Pt single atoms and TiO_2_. Angew. Chem. Int. Ed..

[33] Schwartz V (2007). Structural investigation of Au catalysts on TiO_2_−SiO_2_ supports: nature of the local structure of Ti and Au atoms by EXAFS and XANES. J. Phys. Chem. C..

[34] Zhu Y (2021). Environment of metal–O–Fe bonds enabling high activity in CO_2_ reduction on single metal atoms and on supported nanoparticles. J. Am. Chem. Soc..

[35] Pramhaas V (2021). Interplay between CO disproportionation and oxidation: on the origin of the CO reaction onset on atomic layer deposition-grown Pt/ZrO_2_ model catalysts. ACS Catal..

[36] Galhardo TS (2021). Optimizing active sites for high CO selectivity during CO_2_ hydrogenation over supported nickel catalysts. J. Am. Chem. Soc..

[37] He Y (2020). In situ identification of reaction intermediates and mechanistic understandings of methane oxidation over hematite: a combined experimental and theoretical study. J. Am. Chem. Soc..

[38] Tang C (2020). Coordination tunes selectivity: two-electron oxygen reduction on high-loading molybdenum single-atom catalysts. Angew. Chem. Int. Ed..

[39] Dobrik G (2022). Large-area nanoengineering of graphene corrugations for visible-frequency graphene plasmons. Nat. Nanotechnol..

[40] Chen Y (2018). Identifying size effects of Pt as single atoms and nanoparticles supported on FeO_x_ for the water-gas shift reaction. ACS Catal..

[41] Zhang Z (2017). The most active Cu facet for low-temperature water gas shift reaction. Nat. Commun..

[42] Yao S (2017). Atomic-layered Au clusters on α-MoC as catalysts for the low-temperature water-gas shift reaction. Science.

[43] Chen S (2021). Elucidation of active sites for CH_4_ catalytic oxidation over Pd/CeO_2_ via tailoring metal–support interactions. ACS Catal..

[44] Marcinkowski MD (2018). Pt/Cu single-atom alloys as coke-resistant catalysts for efficient C–H activation. Nat. Chem..

[45] Zhao Z-J, Chiu C-C, Gong J (2015). Molecular understandings on the activation of light hydrocarbons over heterogeneous catalysts. Chem. Sci..

[46] Kresse G, Hafner J (1993). Ab initio molecular dynamics for liquid metals. Phys. Rev. B.

[47] Pan Y (2009). Highly ordered, Millimeter-scale, continuous, single-crystalline graphene monolayer formed on Ru (0001). Adv. Mater..

