# Supplementary material for: A strong bimetal-support interaction in ethanol steam reforming
Source: Nat Commun. 2023 Jun 2;14:3189. doi: 10.1038/s41467-023-38883-x (PMC10238400; doi:10.1038/s41467-023-38883-x)
Supplement: Supplementary file 1 — Supplementary Information [file 41467_2023_38883_MOESM1_ESM.pdf]

Supplementary Information

A strong bimetal-support interaction in ethanol steam reforming

Hao Meng[1], Yusen Yang[1]*, Tianyao Shen[1], Wei Liu[1], Lei Wang[1], Pan Yin[1], Zhen Ren[1], Yiming Niu[2], Bingsen Zhang[2], Lirong Zheng[3], Hong Yan[1], Jian Zhang[1]*, Feng-Shou Xiao[1,4]*, Min Wei[1]*, Xue Duan[1]

[1]State Key Laboratory of Chemical Resource Engineering, Beijing Advanced Innovation Center for Soft Matter Science and Engineering, Beijing University of Chemical Technology, Beijing 100029, P. R. China.

[2]Shenyang National Laboratory for Materials Science, Institute of Metal Research, Chinese Academy of Sciences, Shenyang 110016, P. R. China.

[3]Institute of High Energy Physics, Chinese Academy of Sciences, Beijing 100049, P. R. China.

[4]Key Lab of Biomass Chemical Engineering of Ministry of Education, College of Chemical and Biological Engineering, Zhejiang University, Hangzhou 310027, P. R. China.

Author Information

***** Corresponding authors. Tel: +86-10-64412131; Fax: +86-10-64425385.

E-mail addresses: yangyusen@buct.edu.cn (Y. Yang); jianzhangbuct@buct.edu.cn (J. Zhang); fsxiao@zju.edu.cn (F. -S Xiao); weimin@mail.buct.edu.cn (M. Wei).

Studies on External and Internal diffusion Effects for ESR Tests[1,2]

$$k_c = \frac{(1-\phi)D_{AB}\,Sh'}{\phi d_p} \quad (1)$$

$$Sh' = (Re')^{1/2}\,Sc^{1/3} \quad (2)$$

$$Sc = \frac{\nu}{D_{AB}} \quad (3)$$

$$Re' = \frac{U\,d_p}{(1-\phi)\nu\gamma} \quad (4)$$

$$U = \frac{\upsilon_0}{A_c} \quad (5)$$

$$D_{AB} = \frac{1}{3}l\mu\,(1-e^{\frac{-2r_p}{l}}) \quad (6)$$

$$De = \frac{D_{AB}\phi_p\sigma_c}{\tau} \quad (7)$$

$$\eta = (\frac{5}{16\sigma^2})(\frac{m\mathrm{k}T}{\pi})^{1/2} \quad (8)$$

$$\nu = \frac{\eta}{\rho} \quad (9)$$

Where $-r_A'$ = reaction rate, kmol kg_{cat}^{-1} s^{-1}

n = reaction order

R = catalyst particle radius, m

γ = shape factor, 1.0

ρ_b = bulk density of catalyst bed, kg m^{-3}

= $(1-\phi)$ (ϕ = porosity or void fraction of packed bed)

ρ_c = solid catalyst density, kg m^{-3}

C_{Ab} = bulk gas concentration of A, kmol m^{-3}

k_c = mass transfer coefficient, m s^{-1}

ν = kinematic viscosity, m^2 s^{-1}

d_p = particle diameter, m

U = superficial velocity, m s^{-1}

υ_0 = volumetric flow rate, m^3 s^{-1}

A_c = cross-sectional area of the tube, m^2

D_{AB} = gas-phase diffusivity, m^2 s^{-1}

D_e = effective gas-phase diffusivity, m^2 s^{-1}

l = molecular average free path, m

μ = average molecular rate of motion, m s^{-1}

ϕ_p = pellet porosity

σ_c = constriction factor

τ = tortuosity

s = molecular diameter, Å

k = Boltzmann's constant, J K^{-1}

m = the mass of the molecule, g

η = viscosity

According to the Mears Criterion for external diffusion, if $\frac{-r_A' \rho_b R n}{k_c C_{Ab}} < 0.15$, then external mass transfer effects can be neglected.

$\frac{-r_A' \rho_b R n}{k_c C_{Ab}}$ = (3.86×10^{-5} kmol kg$_{cat}^{-1}$ s^{-1})(452 kg m^{-3})(3.75×10^{-4} m)^2/[(6.3×10^{-3} m^2 s^{-1})(3.32×10^{-3} kmol m^{-3})] = 1.17×10^{-4} < 0.15

According to the Weisz-Prater Criterion for internal diffusion, if $C_{WP} = \frac{-r'_{A(obs)} \rho_c R^2}{D_e C_{As}} < 1$, then internal mass transfer effects can be neglected.

$C_{WP} = \frac{-r'_{A(obs)} \rho_c R^2}{D_e C_{As}}$ = (3.86×10^{-5} kmol kg$_{cat}^{-1}$ s^{-1})(567 kg m^{-3})(3.75×10^{-4} m)^2/[(4.44×10^{-6} m^2 s^{-1})(3.32×10^{-3} kmol m^{-3})] = 0.209 < 1

Therefore, in this work, both external and internal diffusion for ESR tests can be neglected.

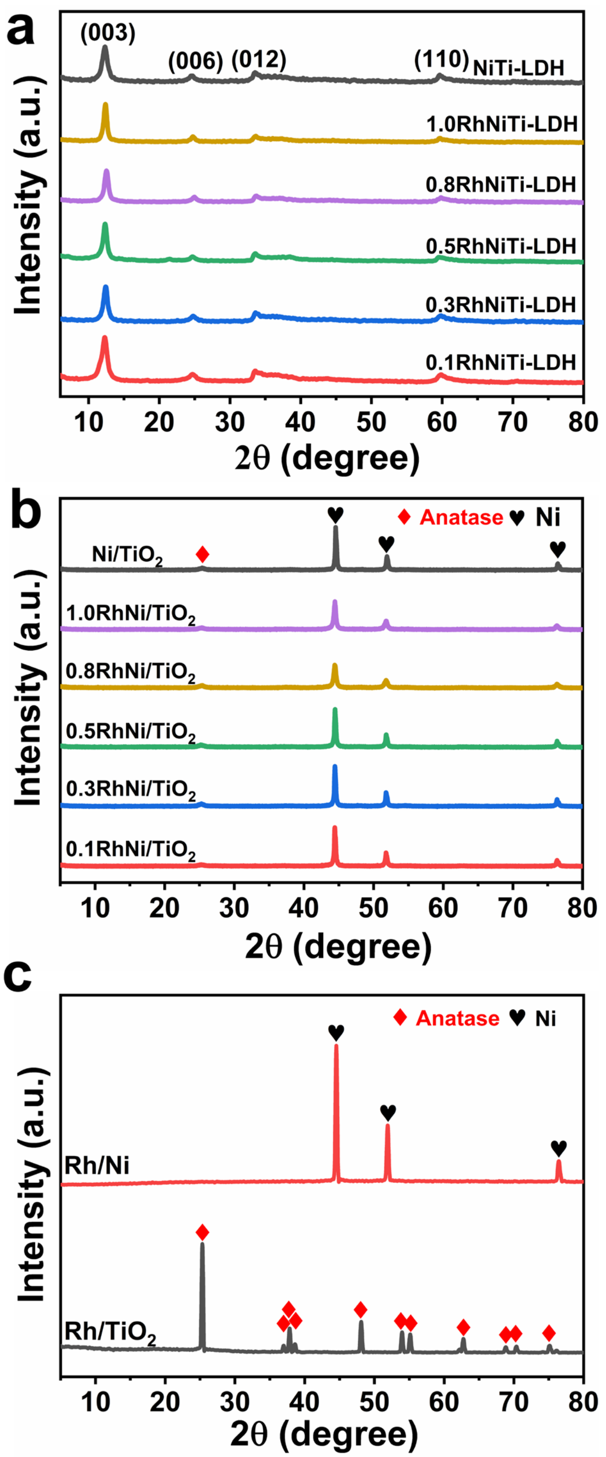

Supplementary Figure 1. Structure characterization of various samples. XRD patterns of **a** LDH precursors, **b**, **c** as-prepared Ni/TiO_2, 0.1RhNi/TiO_2, 0.3RhNi/TiO_2, 0.5RhNi/TiO_2, 0.8RhNi/TiO_2, 1.0RhNi/TiO_2, Rh/Ni and Rh/TiO_2 catalysts.

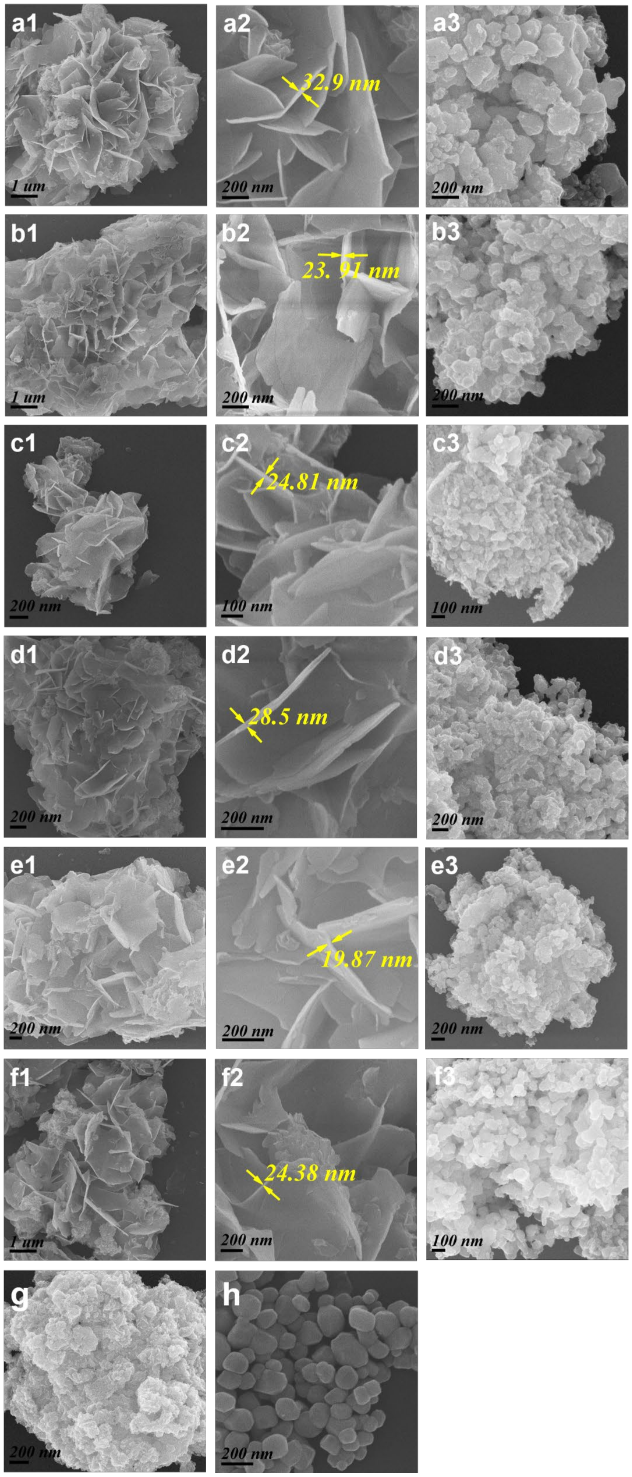

Supplementary Figure 2. Structure characterization of various samples. SEM images of various samples: **a1**, **a2** NiTi-LDH; **b1**, **b2** 0.1RhNiTi-LDH; **c1**, **c2** 0.3RhNiTi-LDH; **d1**, **d2** 0.5RhNiTi-LDH; **e1**, **e2** 0.8RhNiTi-LDH; **f1**, **f2** 1.0RhNiTi-LDH; **a3** Ni/TiO_2; **b3** 0.1RhNi/TiO_2; **c3** 0.3RhNi/TiO_2; **d3** 0.5RhNi/TiO_2; **e3** 0.8RhNi/TiO_2; **f3** 1.0RhNi/TiO_2; **g** Rh/Ni and **h** Rh/TiO_2.

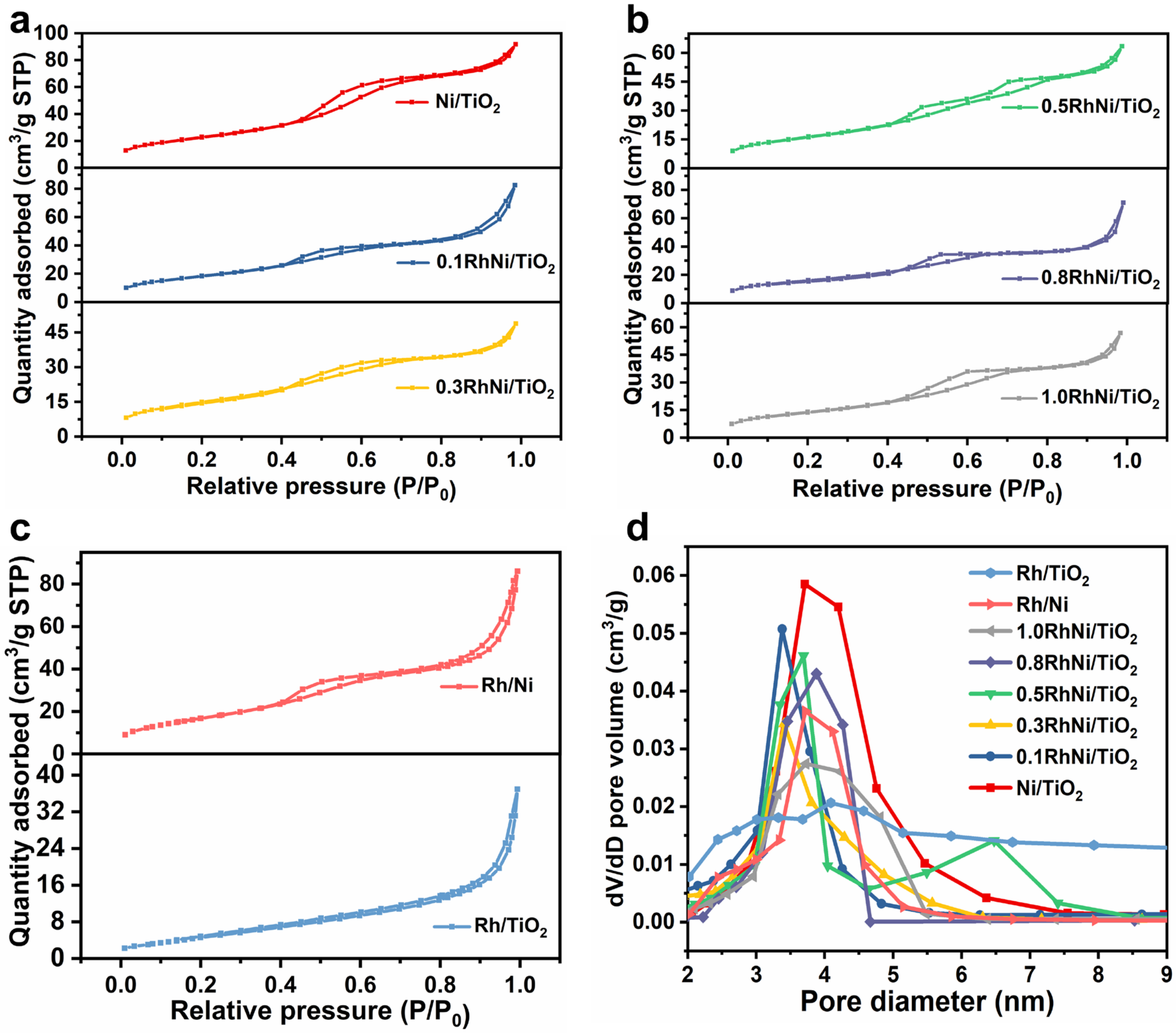

Supplementary Figure 3. N_2 adsorption–desorption experiment of various samples. a-c N_2-adsoption and desorption curves and **d** pore size distribution of Ni/TiO_2, $0.1RhNi/TiO_2$, $0.3RhNi/TiO_2$, $0.5RhNi/TiO_2$, $0.8RhNi/TiO_2$, $1.0RhNi/TiO_2$, Rh/Ni and Rh/TiO_2 samples.

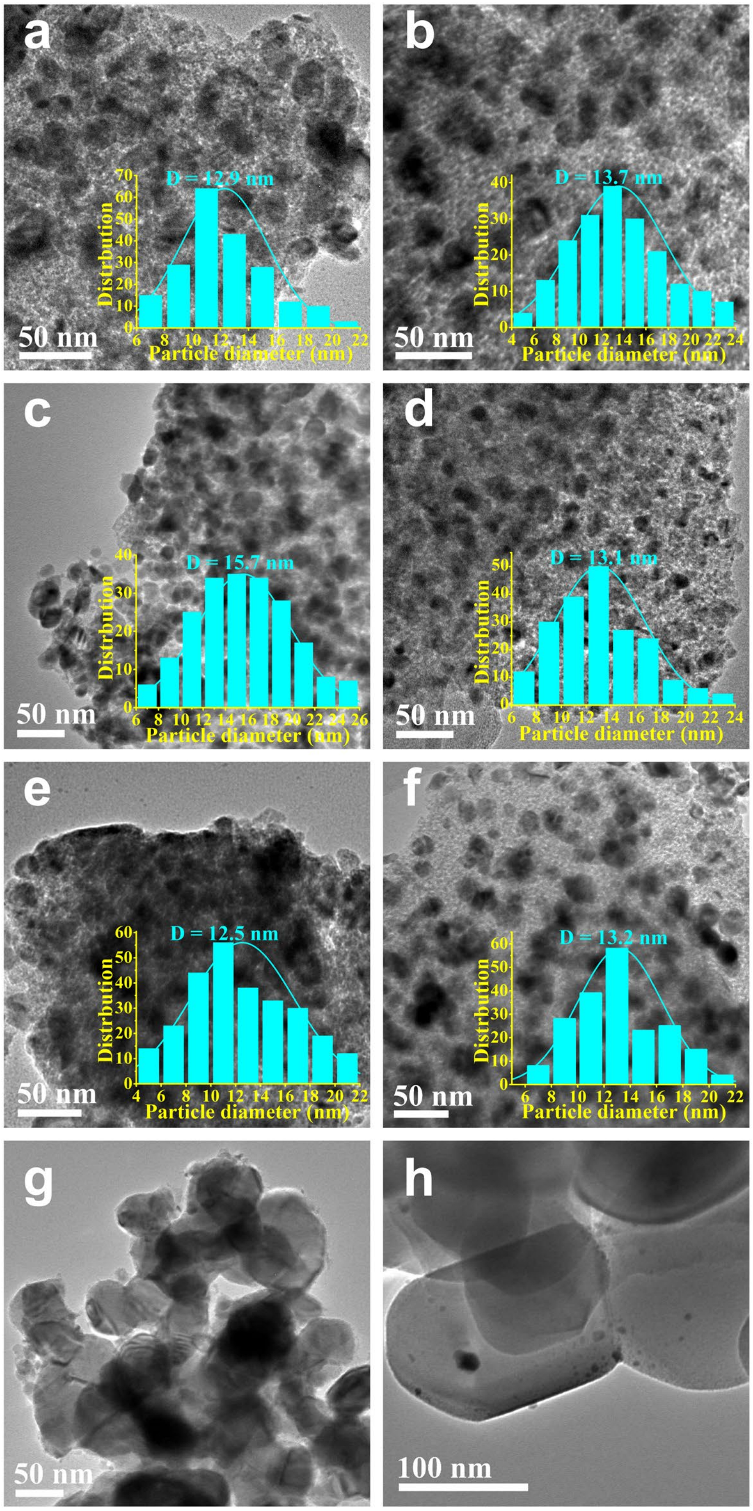

Supplementary Figure 4. Structure characterization of various samples. TEM images of **a** Ni/TiO_2, **b** 0.1RhNi/TiO_2, **c** 0.3RhNi/TiO_2, **d** 0.5RhNi/TiO_2, **e** 0.8RhNi/TiO_2, **f** 1.0RhNi/TiO_2, **g** Rh/Ni and **h** Rh/TiO_2 (inset: the histogram for size distribution of Ni nanoparticles).

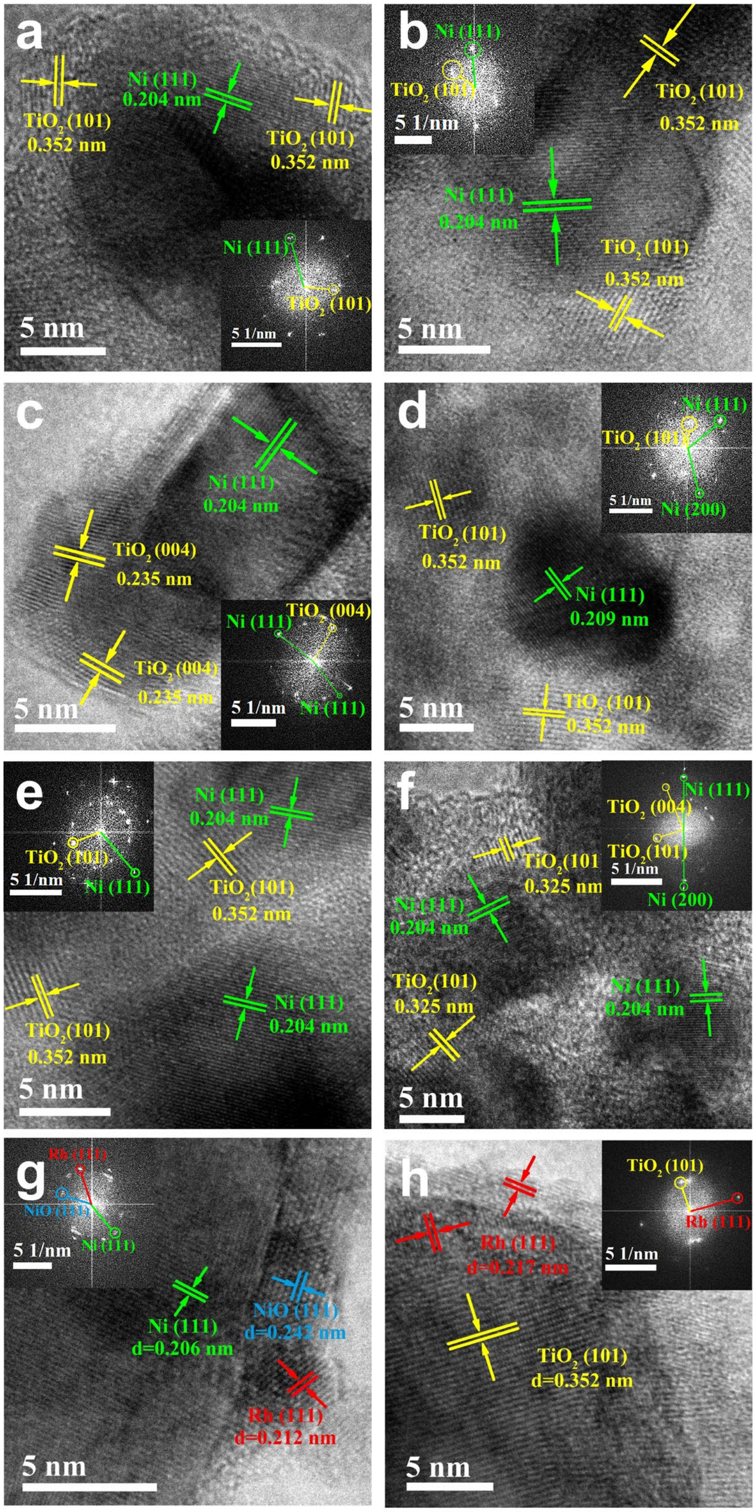

Supplementary Figure 5. Structure characterization of various samples. HR-TEM images of **a** Ni/TiO_2, **b** 0.1RhNi/TiO_2, **c** 0.3RhNi/TiO_2, **d** 0.5RhNi/TiO_2, **e** 0.8RhNi/TiO_2, **f** 1.0RhNi/TiO_2 **g** Rh/Ni and **h** Rh/TiO_2 (inset: fast Fourier transform (FFT) patterns of panel).

Supplementary Note 1

The XRD patterns of *x*RhNiTi-LDHs precursors show a series of characteristic diffraction peaks of hydrotalcite-like structure (003, 006, 012 and 110) without other impure phase (Supplementary Fig. 1a)[3,4]. As shown in the SEM images (Supplementary Figs. 2a1-f1 and Supplementary Figs. 2a2-f2), the introduction of trace Rh does not significantly affect its surface topography, and all these *x*RhNiTi-LDHs samples display a frizzy flowerlike morphology. After a treatment in H_2 atmosphere at 400 °C, the crystallite surface becomes rough with high specific surface area and abundant pore structure (Supplementary Figs. 2a3-f3, Supplementary Fig. 3 and Supplementary Table 1). The diffraction peaks at 2θ 44.5°, 51.9° and 76.5° are indexed to a face-centered cubic (fcc) Ni phase (JCPDS 7440-02-0), and the reflection at 2θ 25.3° corresponds to anatase TiO_2 phase (JCPDS 21-1272) (Supplementary Fig. 1b). The control samples of Rh/TiO_2 and Rh/Ni show characteristic diffraction peaks of anatase TiO_2 phase (JCPDS 21-1272) and fcc Ni phase (JCPDS 7440-02-0), respectively (Supplementary Fig. 1c). No reflection associated with Rh is observed for all these samples, suggesting that Rh species exists at a high dispersion or its content is below the detection limit. Transmission electron microscopy (TEM) images of *x*RhNi/TiO_2 samples show that Ni nanoparticles are well-dispersed within the TiO_2 matrix with similar particle size of 12−16 nm (Supplementary Figs. 4a-4f). From the HR-TEM images (Supplementary Figs. 5a-5f), two clear crystalline phases are identified for Ni/TiO_2 and *x*RhNi/TiO_2 samples: the lattice fringe of 0.204 nm corresponds to the Ni(111) plane of cubic phase and that of 0.352 nm is due to the TiO_2(101) plane from anatase phase. Owing to the high dispersion of Rh as well as similar Z-contrast between Rh and Ni, it is hard to ascertain the distribution of Rh element in *x*RhNi/TiO_2 samples. However, for the Rh/Ni and Rh/TiO_2 control samples, except for the existence of lattice stripes belonging to Ni and TiO_2, the Rh(111) lattice

stripes on the surface of Ni and TiO_2 are identified, respectively (Supplementary Figs. 5g and 5h). The results above indicate that the interaction between Ni nanoparticles and TiO_2 support favors a high dispersion of Rh species in the case of *x*RhNi/TiO_2 samples.

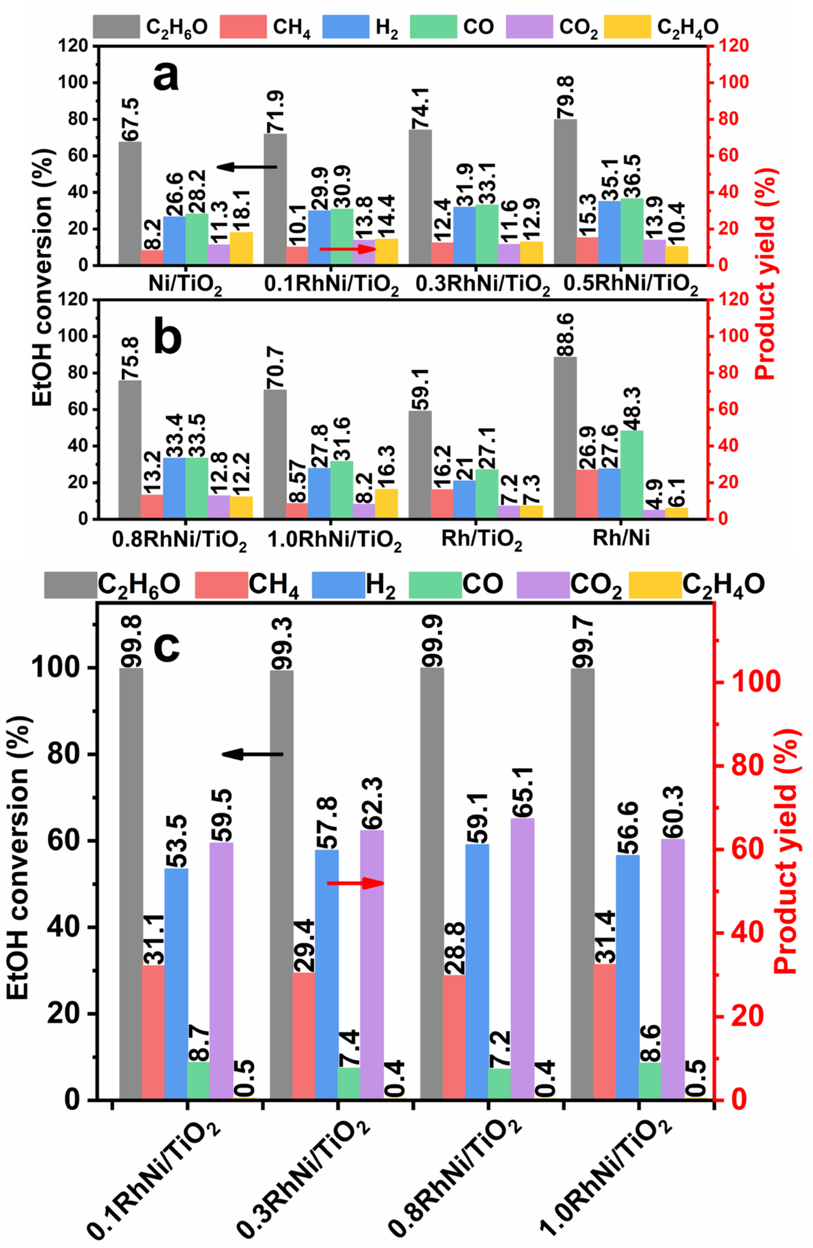

Supplementary Figure 6. Catalytic activity of various samples. Ethanol conversion and products yield over various catalysts at **a**, **b** 350 °C and **c** 400 °C (ESR reaction conditions: catalyst (0.15 g) + SiO_2 (1.50 g); liquid feed of S/C = 3 at 0.060 mL min^{-1}; N_2 carrier at 50.0 mL min^{-1}; reaction temperature: 350/400 °C; time on stream: 1.5 h).

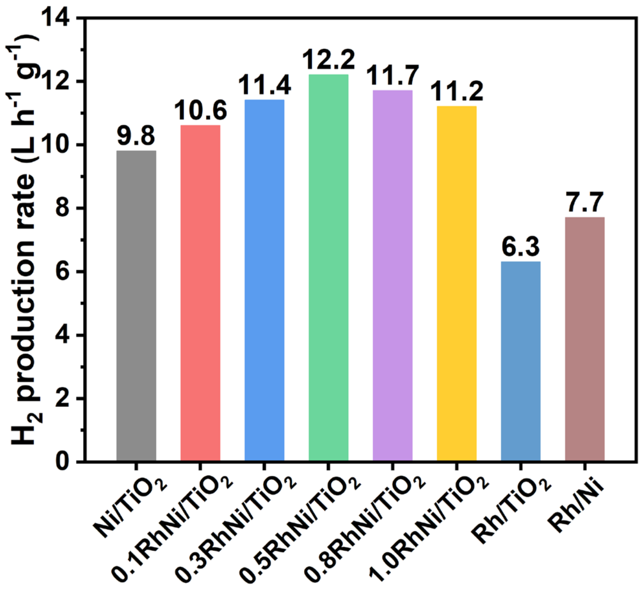

Supplementary Figure 7. Catalytic activity of various samples. H_2 production rate over various catalysts (ESR reaction conditions: catalyst (0.15 g) + SiO_2 (1.50 g); liquid feed of S/C = 3 at 0.060 mL min^{-1}; N_2 carrier at 50.0 mL min^{-1}; reaction temperature: 400 °C, time on stream: 1.5 h).

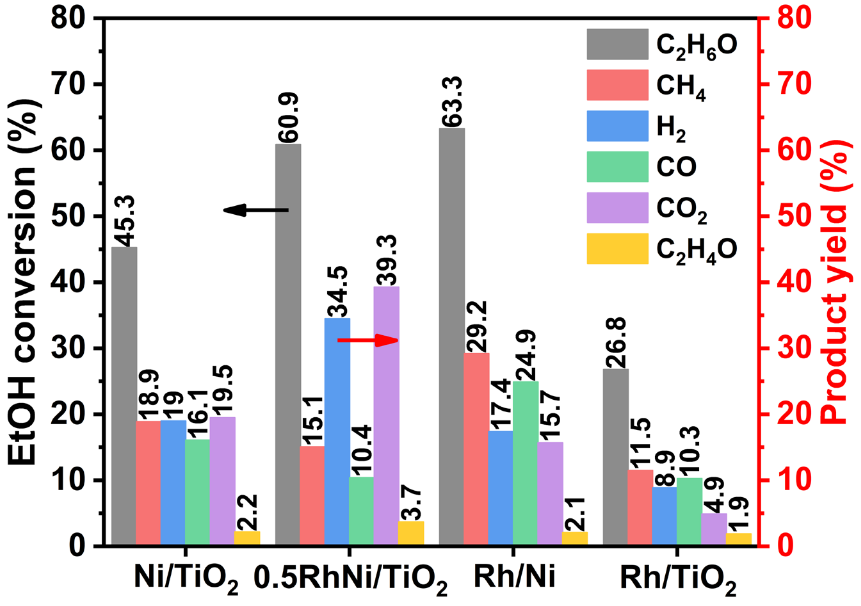

Supplementary Figure 8. Catalytic activity of various samples. Ethanol conversion and product yield over various catalysts. ESR reaction conditions: catalyst (0.05 g) + SiO_2 (0.50 g); liquid feed of S/C = 3 at 0.060 mL min^{-1}; N_2 carrier at 50.0 mL min^{-1}; reaction temperature: 400 °C; time on stream: 1.5 h.

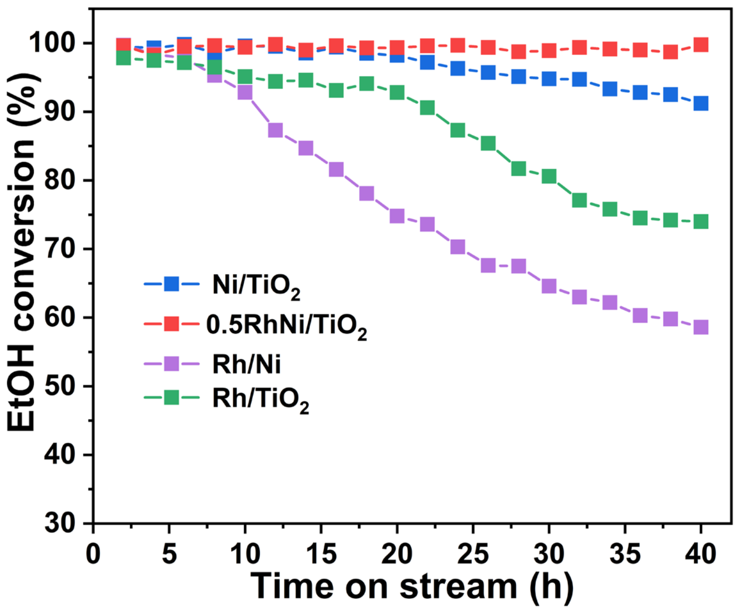

Supplementary Figure 9. Catalytic activity of various samples. Time on stream (TOS) tests for Ni/TiO_2, 0.5RhNi/TiO_2, Rh/Ni and Rh/TiO_2 at 400 °C, respectively.

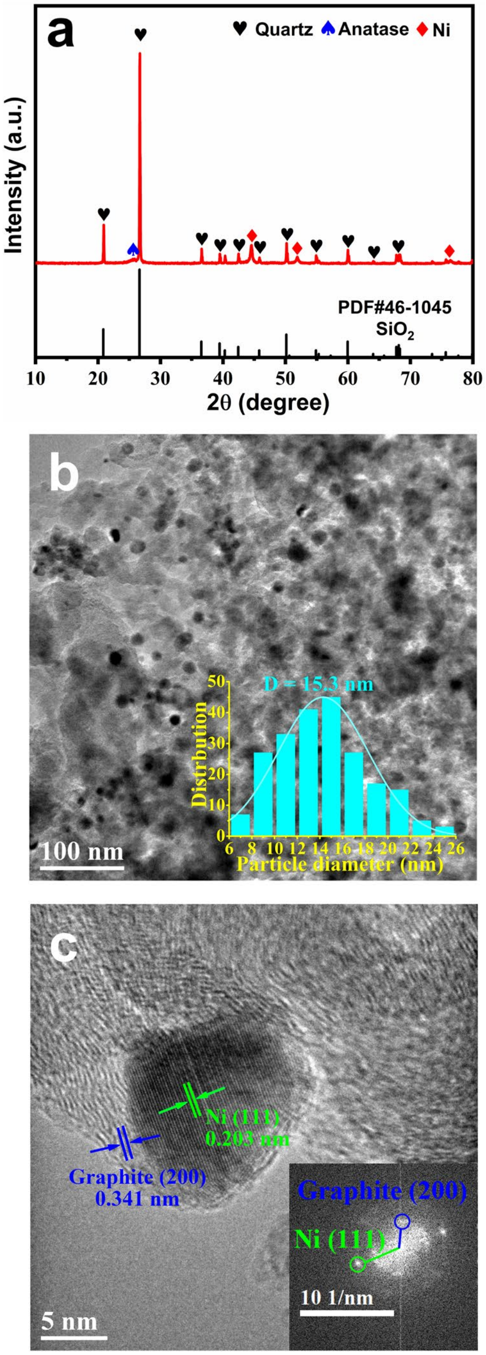

Supplementary Figure 10. Structure characterization of used 0.5RhNi/TiO_2 catalyst. a XRD pattern, **b** TEM and **c** HR-TEM images of 0.5RhNi/TiO_2 catalyst after 300 h of stability test. The quartz crystal structure displayed in **a** could be attributed to the remains of quartz sand during the catalyst retrieve process.

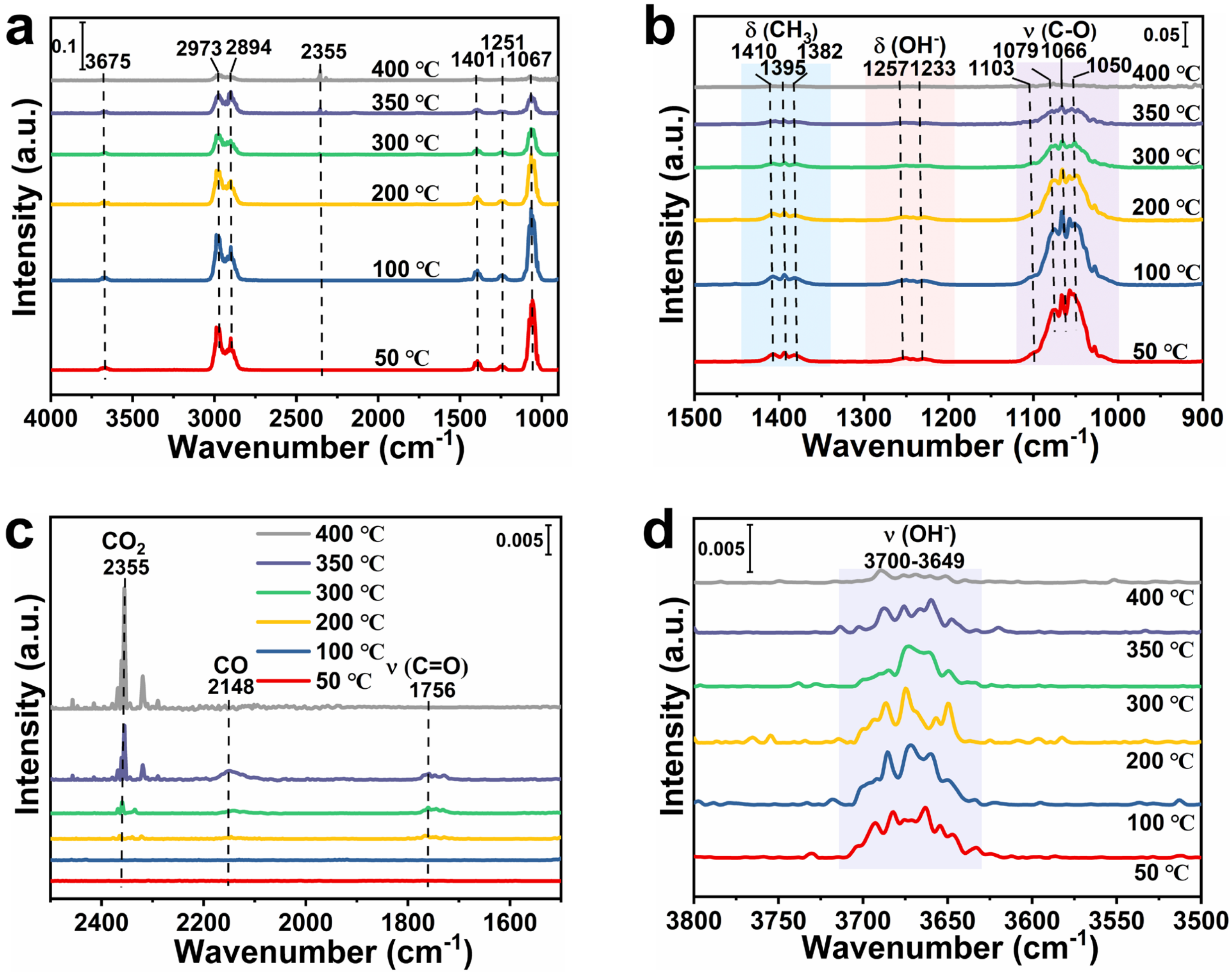

Supplementary Figure 11. ***Operando*** **DRIFTS spectra of 0.5RhNi/TiO2 catalyst. a** *Operando* DRIFTS spectra of ethanol and water (S/C = 3) chemisorption over 0.5RhNi/TiO_2 catalyst from 50 to 400 °C. **b-d** Local magnification regions in (a).

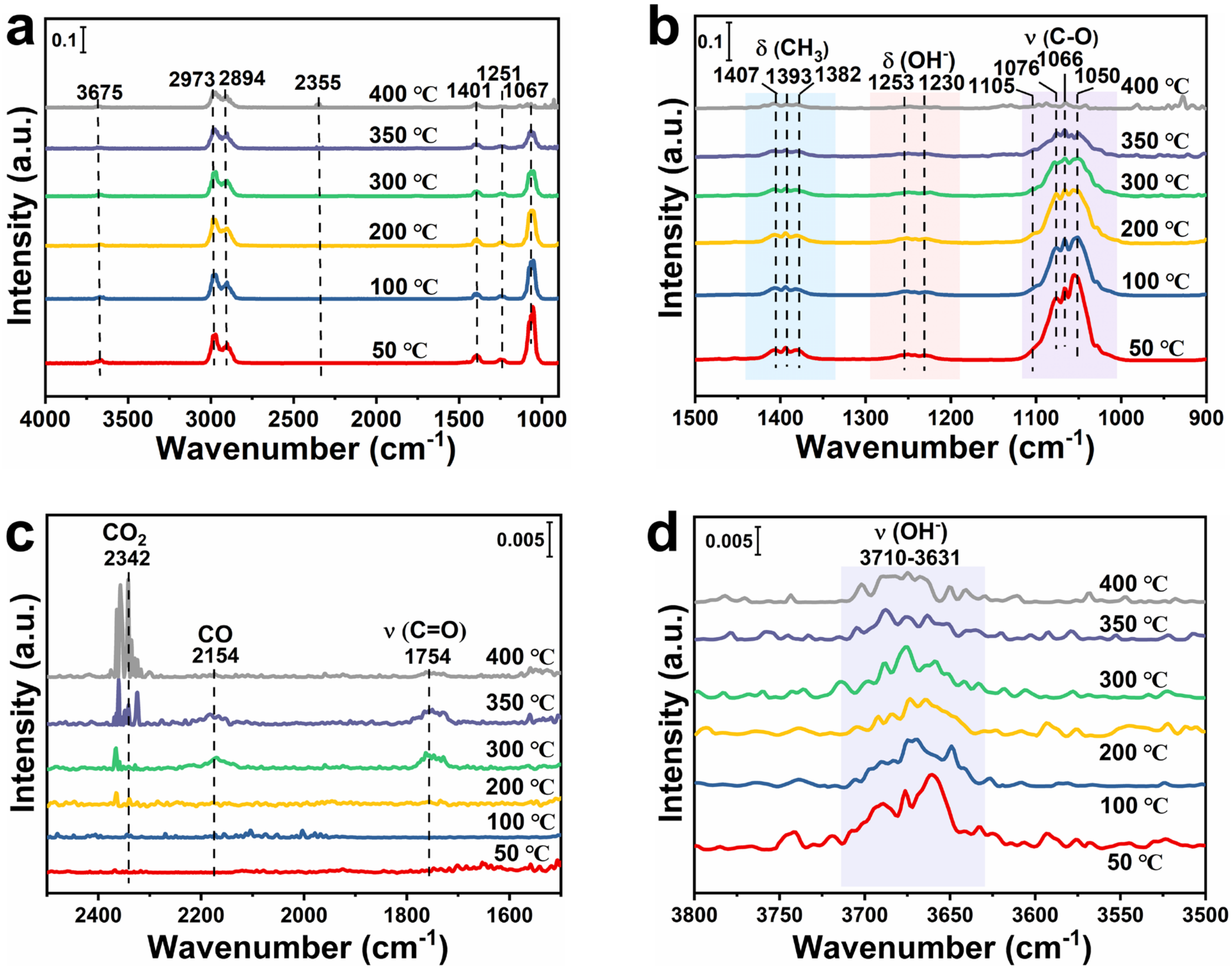

Supplementary Figure 12. *Operando* DRIFTS spectra of Ni/TiO$_2$ catalyst. a *Operando* DRIFTS spectra of ethanol and water (S/C = 3) chemisorption over Ni/TiO$_2$ catalyst from 50 to 400 °C. **b-d** Local magnification regions in (a).

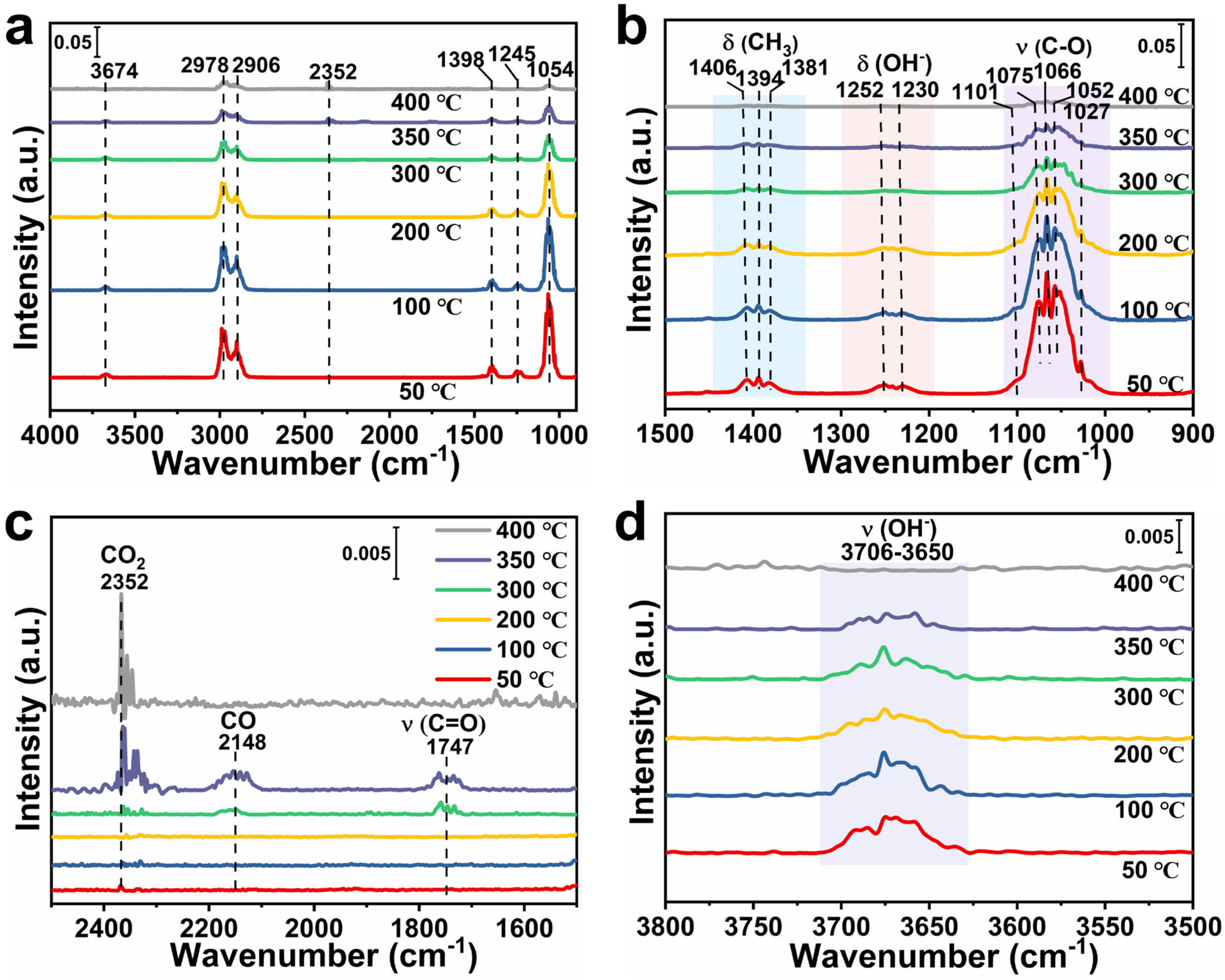

Supplementary Figure 13. *Operando* DRIFTS spectra of Rh/Ni catalyst. a *Operando* DRIFTS spectra of ethanol and water (S/C = 3) chemisorption over Rh/Ni catalyst from 50 to 400 °C. **b-d** Local magnification regions in (a).

Supplementary Note 2

According to previous studies, two reaction paths are normally proposed on ESR reaction: (1) ethanol dehydrogenation plus acetaldehyde decomposition to generate CO, CH_x and H_2, followed by steam reforming of CO and CH_x to produce CO_2 and H_2; (2) ethanol dehydrogenation plus acetaldehyde oxidation (by H_2O derived OH or O species) to acetate followed by decomposition to generate CO_2 and H_2[5-7]. As shown in Supplementary Fig. 11, the adsorption of ethanol on 0.5RhNi/TiO_2 catalyst results in adsorbed ethoxide species at 50 °C, where the band at

1050–1103 cm^{-1} (Supplementary Fig. 11b) is assigned to the *v*(C–O) monodentate and bidentate vibrations of oxethyl. The bands located at 2800–3000 cm^{-1} (Supplementary Fig. 11a) are attributed to asymmetric and symmetric stretching vibration of methyl and methylene groups, accompanied by the bend vibration mode within 1380–1410 cm^{-1} (Supplementary Fig. 11b)[8]. The bands at 1257 cm^{-1} (Supplementary Fig. 11b) and 3700–3649 cm^{-1} (Supplementary Fig. 11d) are specified as OH group in ethanol with bend vibration and stretching vibration modes, respectively[9]. As the temperature increases to 200–350 °C, the ν(C=O) vibration band assigned to acetaldehyde species is observed at 1756 cm^{-1} (Supplementary Fig. 11c), whose intensity enhances along with the rise of temperature. This signifies the occurrence of ethanol and ethoxy species dehydrogenation to acetaldehyde[10]. In addition, CO and CO_2 adsorption bands appear at 300–350 °C accompanied by the decline of acetaldehyde and ethoxy species, indicating the decomposition of acetaldehyde to produce CO as well as the occurrence of WGS reaction to generate CO_2. When the temperature reaches 400 °C, ethoxy species and acetaldehyde are completely transformed. The residual peaks at 2973 and 2894 cm^{-1} are ascribed to CH_x species[11], whose intensity declines gradually due to its reaction with H_2O to produce CO_2 and H_2. The results demonstrate that the ESR reaction over 0.5RhNi/TiO_2 catalyst obeys ethanol dehydrogenation to acetaldehyde followed by acetaldehyde decomposition to CO and CH_x, rather than the acetate path (Fig. 1a). The conversion of CO and CH_x intermediates is the key step for this path. A similar phenomenon is also found in Ni/TiO_2 (Supplementary Fig. 12) and Rh/Ni catalysts (Supplementary Fig. 13).

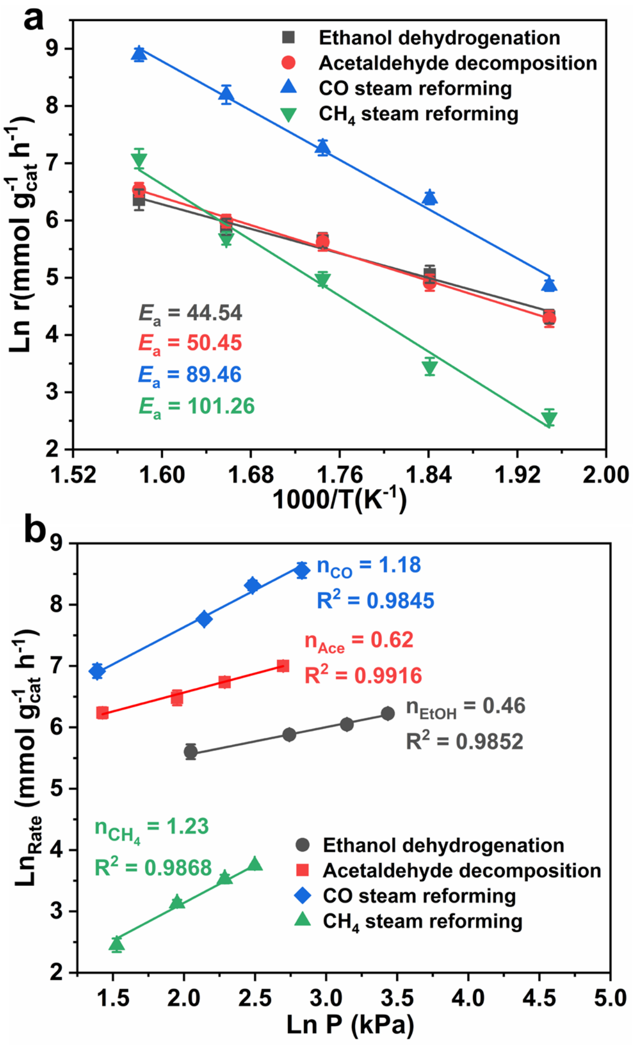

Supplementary Figure 14. Kinetic analysis of 0.5RhNi/TiO_2 catalyst. a Apparent activation energy and **b** reaction order for ethanol dehydrogenation, acetaldehyde decomposition, steam reforming of CO (or CH_4) within kinetic range (conversion less than 10%). Reaction conditions: catalyst (5–30 mg) + SiO_2 (50–300 mg), liquid feed of ethanol (or acetaldehyde) at 0.04 mL min^{-1}, CO at 50 mL min^{-1}, CH_4 at 10 at mL min^{-1}, N_2 carrier at 50 mL min^{-1}, reaction temperature: 240–360 °C, time on stream: 0.5 h.

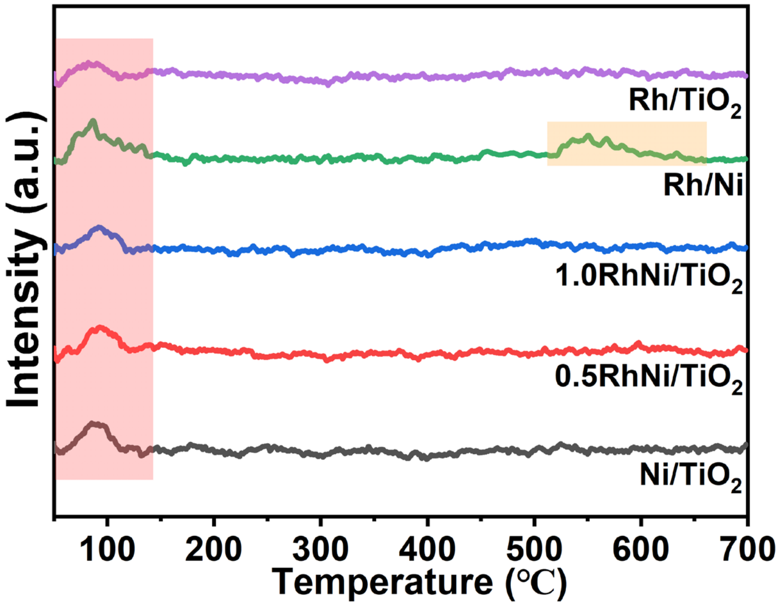

Supplementary Figure 15. CO_2-TPD experiment. CO_2-TPD profiles of various catalysts.

Supplementary Note 3

The CO_2-TPD (Supplementary Fig. 15) profiles show that the Rh/Ni catalyst gives a rather high temperature of CO_2 desorption peak above 500 °C. Such a strong CO_2 adsorption ability would poison catalyst surface, which is the crucial reason for the poor CO conversion during the long-term catalytic reaction.

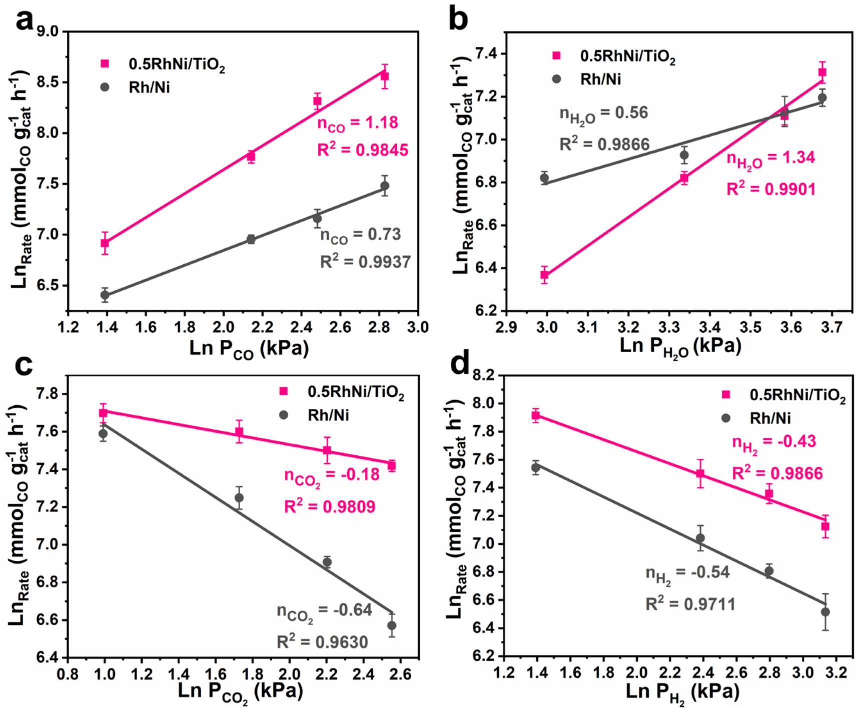

Supplementary Figure 16. Reaction order measurement of 0.5RhNi/TiO_2 and Rh/Ni catalysts. Reaction order of **a** CO, **b** H_2O, **c** CO_2 and **d** H_2 during water-gas shift reaction in the presence of 0.5RhNi/TiO_2 and Rh/Ni catalyst, respectively. Error bars represent the standard deviation from at least three independent measurements.

Supplementary Note 4

As shown in Supplementary Figs. 16a and 16b, the results of reaction order measurement for WGS reaction show that 0.5RhNi/TiO_2 and Rh/Ni give significantly different positive value on CO and H_2O. This is related to their different reaction mechanism, *i.e.*, an associative mechanism in the case of 0.5RhNi/TiO_2 catalyst whilst a redox mechanism on the Rh/Ni catalyst as confirmed by the following *in situ* DRIFT analysis. Remarkably, compared with the 0.5RhNi/TiO_2 catalyst, the Rh/Ni displays a more negative reaction order on CO_2 (Supplementary Fig. 16c), but a similar value on H_2 order (Supplementary Fig. 16d). This implies that the CO_2 has a more negative effect on Rh/Ni than that on 0.5RhNi/TiO_2 catalyst, in addition to the effects from the Le Chatelier's principle (thermodynamic equilibrium).

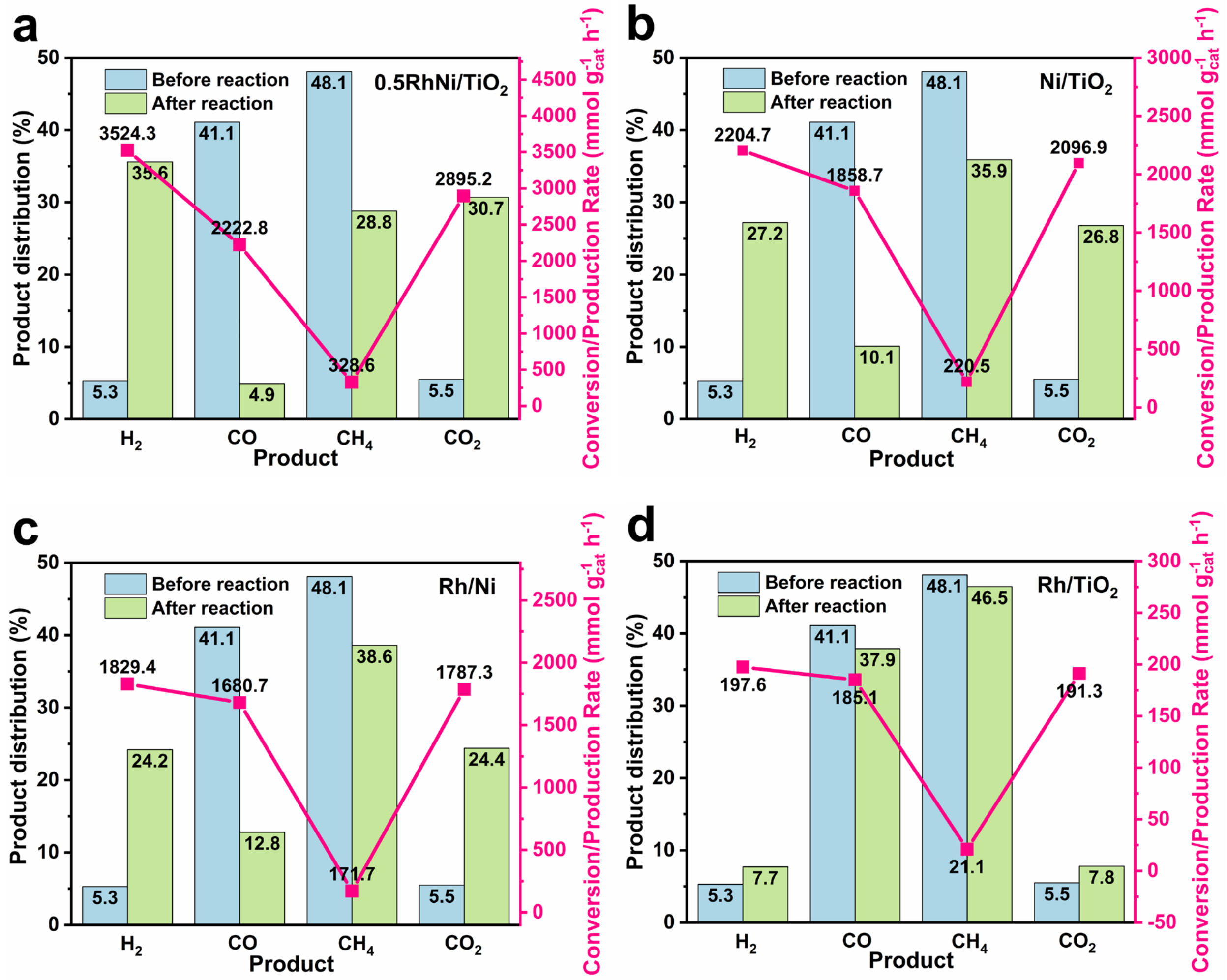

Supplementary Figure 17. Performance test of various samples. Reaction rate, production rate and production distribution of CO, CH_4, H_2 and CO_2 over **a** 0.5RhNi/TiO_2, **b** Ni/TiO_2, **c** Rh/Ni and d Rh/TiO_2 catalyst (Reaction conditions: catalyst (5 mg) + SiO_2 (50 mg); CO at 50 mL min^{-1}; CH_4 at 10 mL min^{-1}; H_2 at 5 mL min^{-1}; CO_2 at 5 mL min^{-1}; N_2 carrier at 30 mL min^{-1}; H_2O at 0.032 mL min^{-1}, 400 °C).

Supplementary Note 5

According to the Le Chatelier's principle, the reaction equilibrium could be affected by the initial gas composition. To simulate the real reaction situation, the reaction rates of CO and CH_4 steam reforming were also determined over these catalysts, where the reactants included H_2O, CO, CH_4, H_2 and CO_2, simultaneously. Based on the results from Supplementary Fig. 17, the

0.5RhNi/TiO_2 catalyst still exhibits the highest reaction rates of CO and CH_4, corresponding to its outstanding catalytic performance. This indicates the advantage of SBMSI in 0.5RhNi/TiO_2 catalyst toward steam reforming reaction.

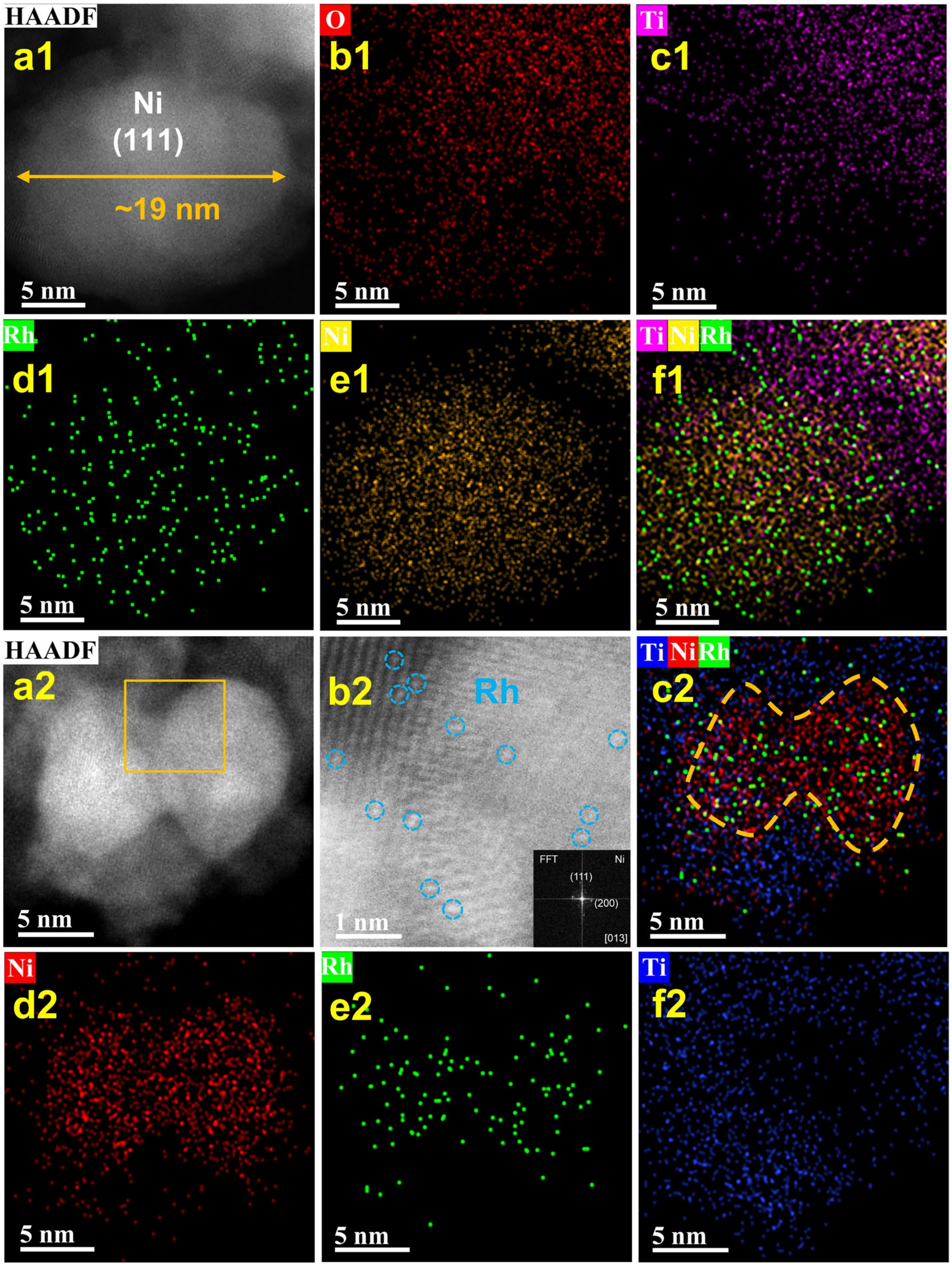

Supplementary Figure 18. Electron micrology studies on the 0.3RhNi/TiO_2. HAADF-STEM images and EDS mapping of 0.3RhNi/TiO_2 sample in various regions **a1-f1** and **a2-f2**. The Rh atoms are distributed uniformly on the surface of Ni nanoparticles to form the RhNi bimetal structure, which further establishes a bimetal-support interface with TiO_2.

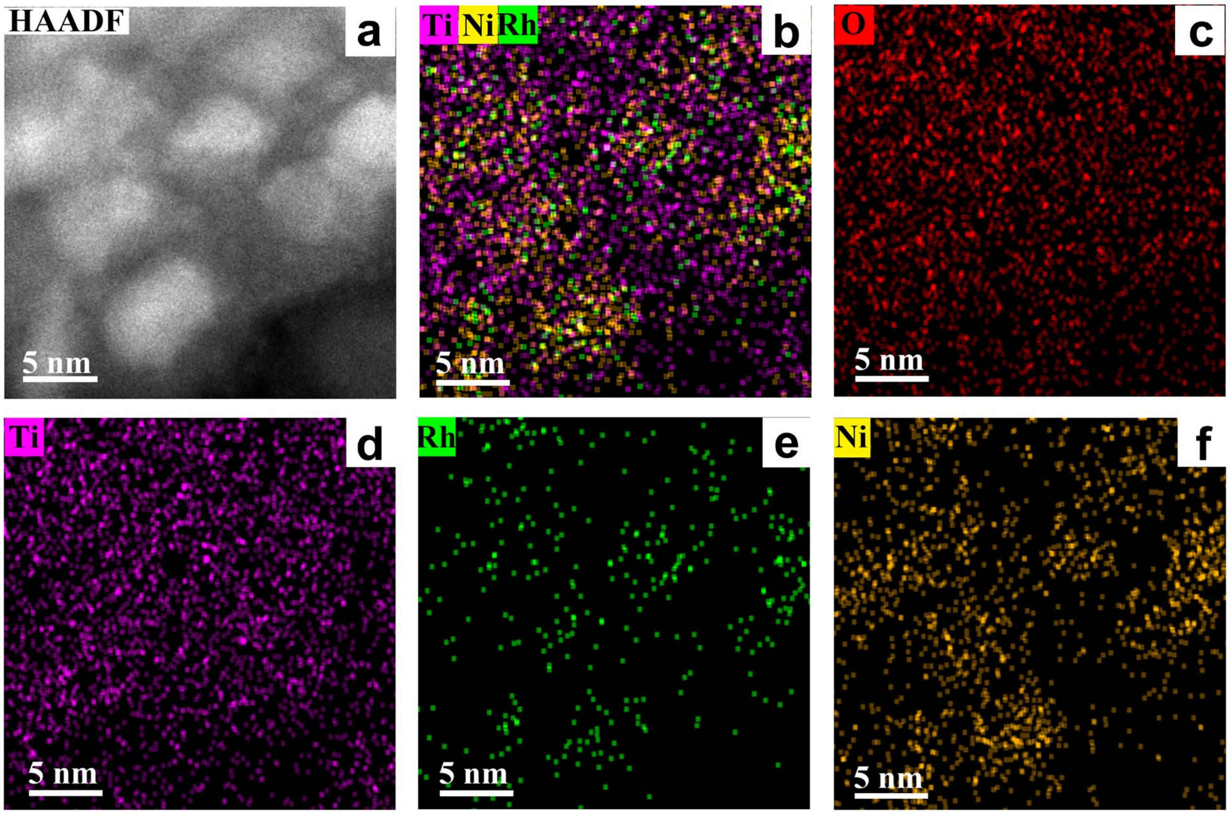

Supplementary Figure 19. Electron micrology studies on the 0.5RhNi/TiO_2. a HAADF-STEM images and **b-f** EDS mapping of 0.5RhNi/TiO_2 sample. The Rh atoms are distributed uniformly on the surface of Ni nanoparticles to form the RhNi bimetal structure, which further establishes a bimetal-support interface with TiO_2.

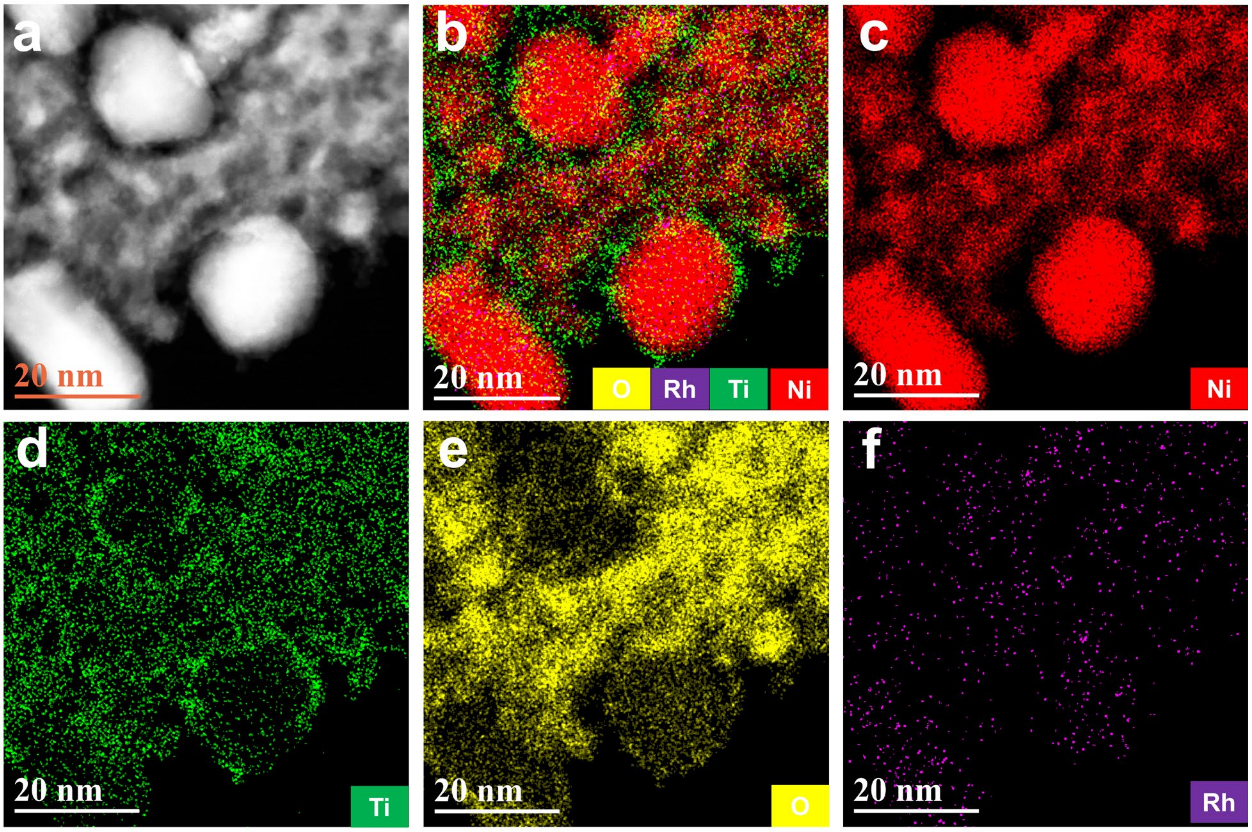

Supplementary Figure 20. Electron micrology studies on the 0.5RhNi/TiO_2. a HAADF-STEM, and **b-f** EDS mapping images of 0.5RhNi/TiO_2 sample after H_2 reduction.

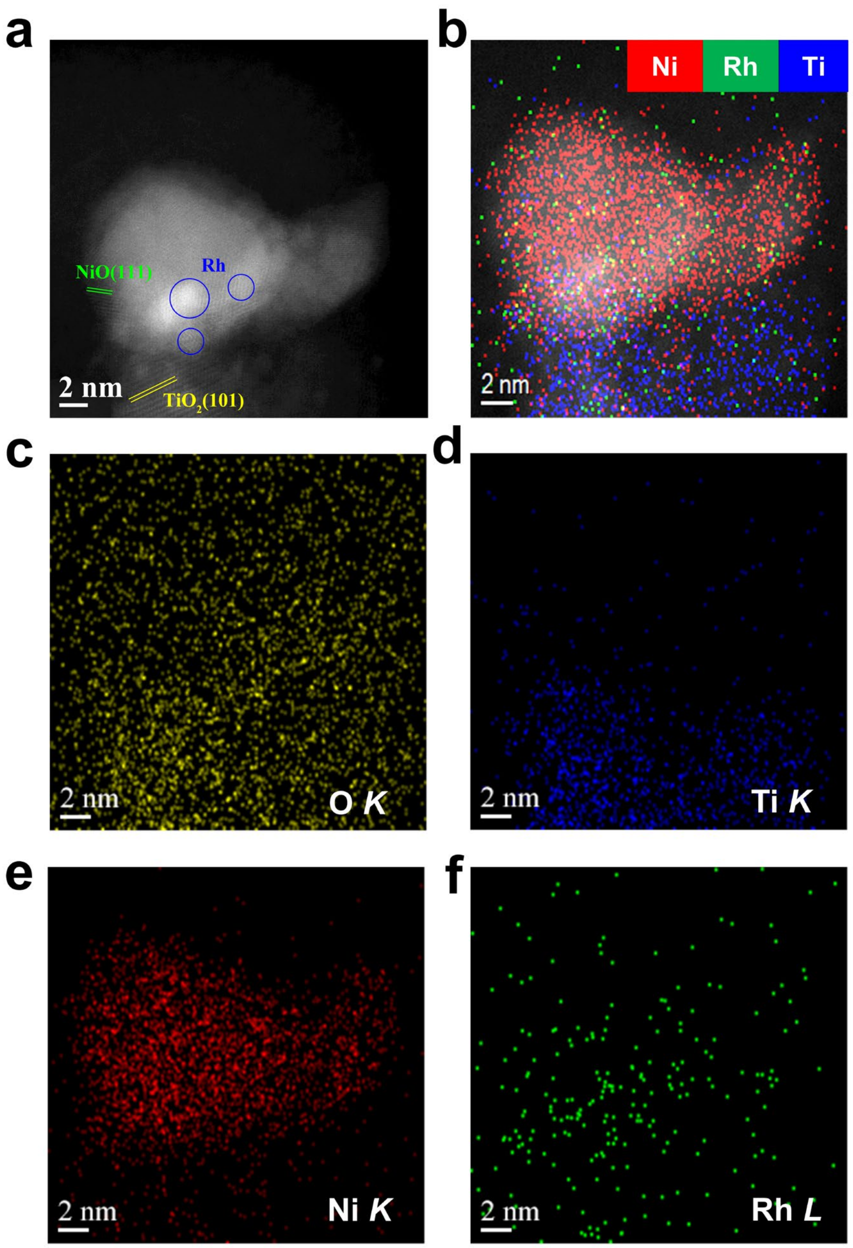

Supplementary Figure 21. Electron micrology studies on the 0.5RhNi/TiO_2. a HAADF-STEM and **b-f** EDS mapping images of 0.5RhNi/TiO_2 sample after a cycle of H_2 reduction-O_2 oxidation.

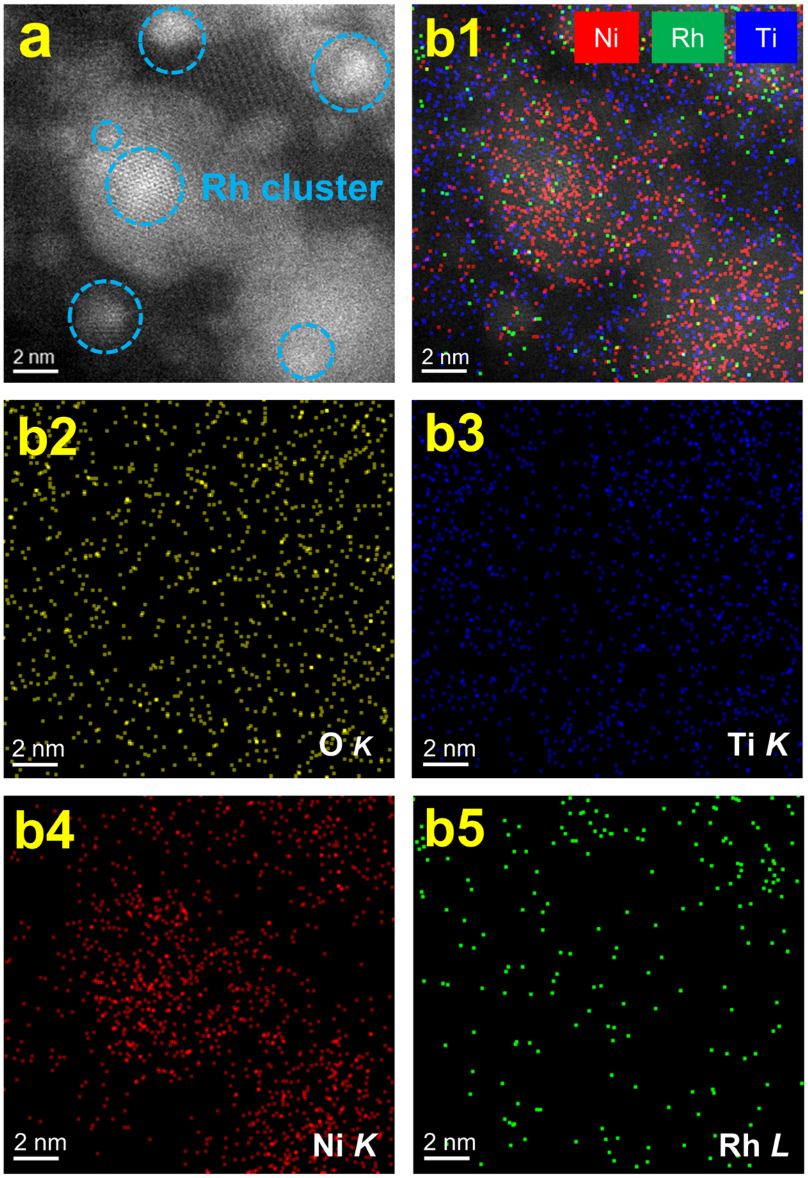

Supplementary Figure 22. Electron micrology studies on the 0.8RhNi/TiO_2. a HAADF-STEM images and **b1-b5** EDS mapping of 0.8RhNi/TiO_2 sample. A relatively high Rh content of 0.8 wt.% induces an aggregation of Rh species on the surface of Ni nanoparticle.

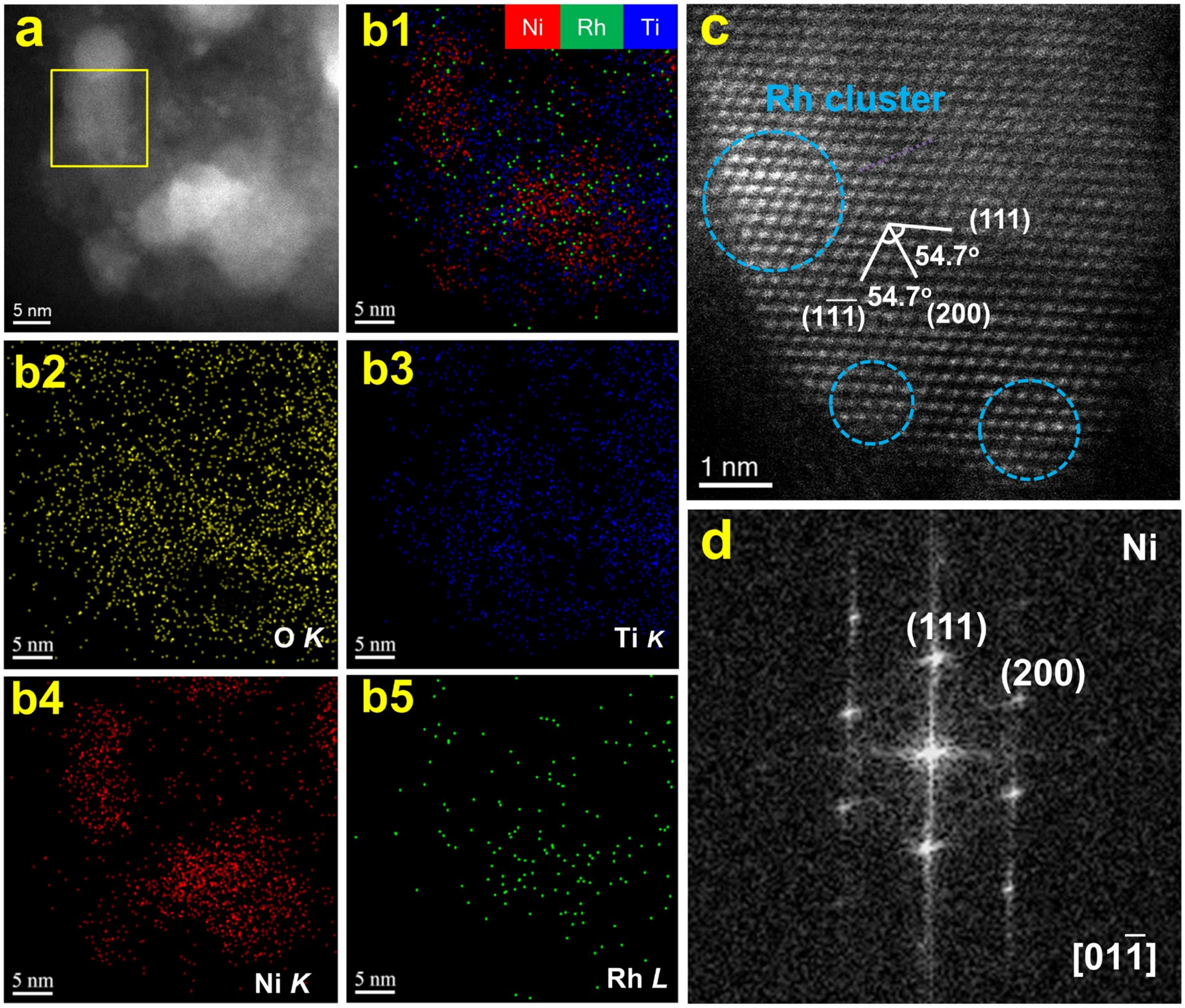

Supplementary Figure 23 Electron micrology studies on the 1.0RhNi/TiO_2. a, **c** HAADF-STEM images and **b1-b5** EDS mapping of 1.0RhNi/TiO_2 sample. **d** Fast Fourier Transform (FFT) patterns of panel. A relatively high Rh content of 0.8 *wt.*% and 1.0 *wt.*% induces an aggregation of Rh species on the surface of Ni nanoparticle.

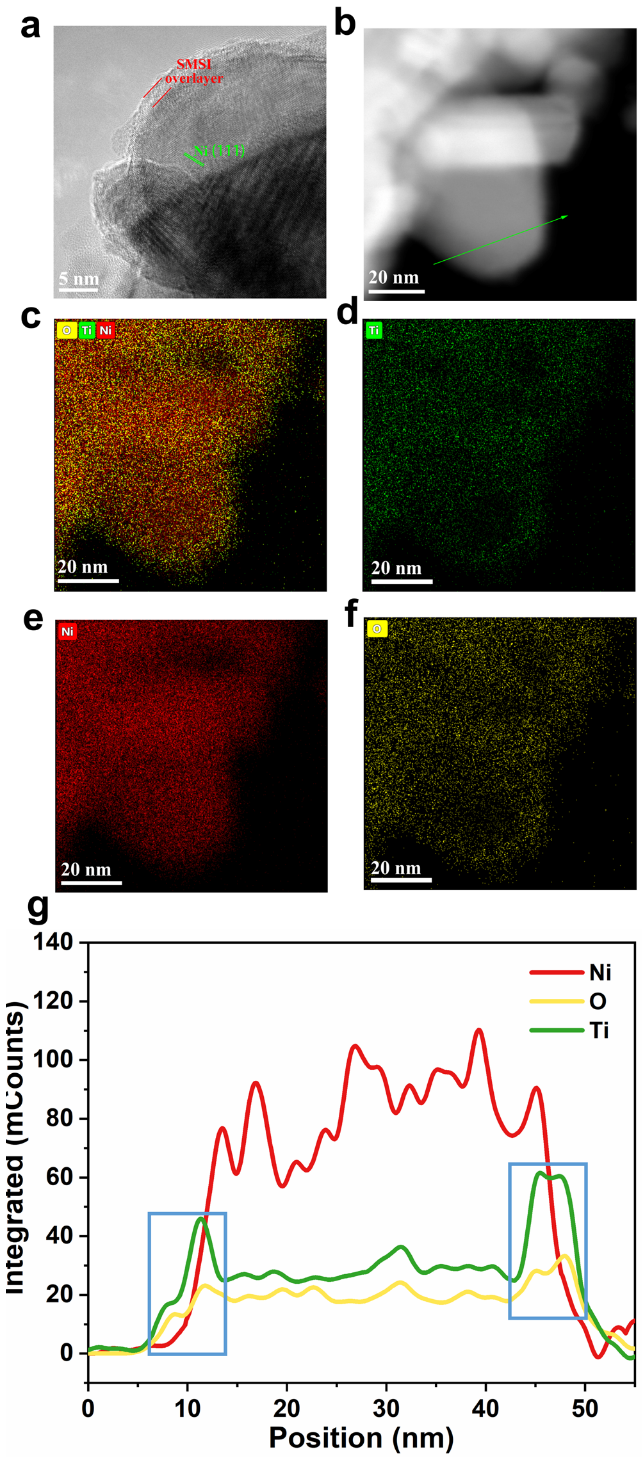

Supplementary Figure 24. Electron micrology studies on the Ni/TiO_2. a HR-TEM, **b** ac-HAADF, **c-f** EDS mapping images and **g** line scan from **b** for the Ni/TiO_2 sample after H_2 reduction.

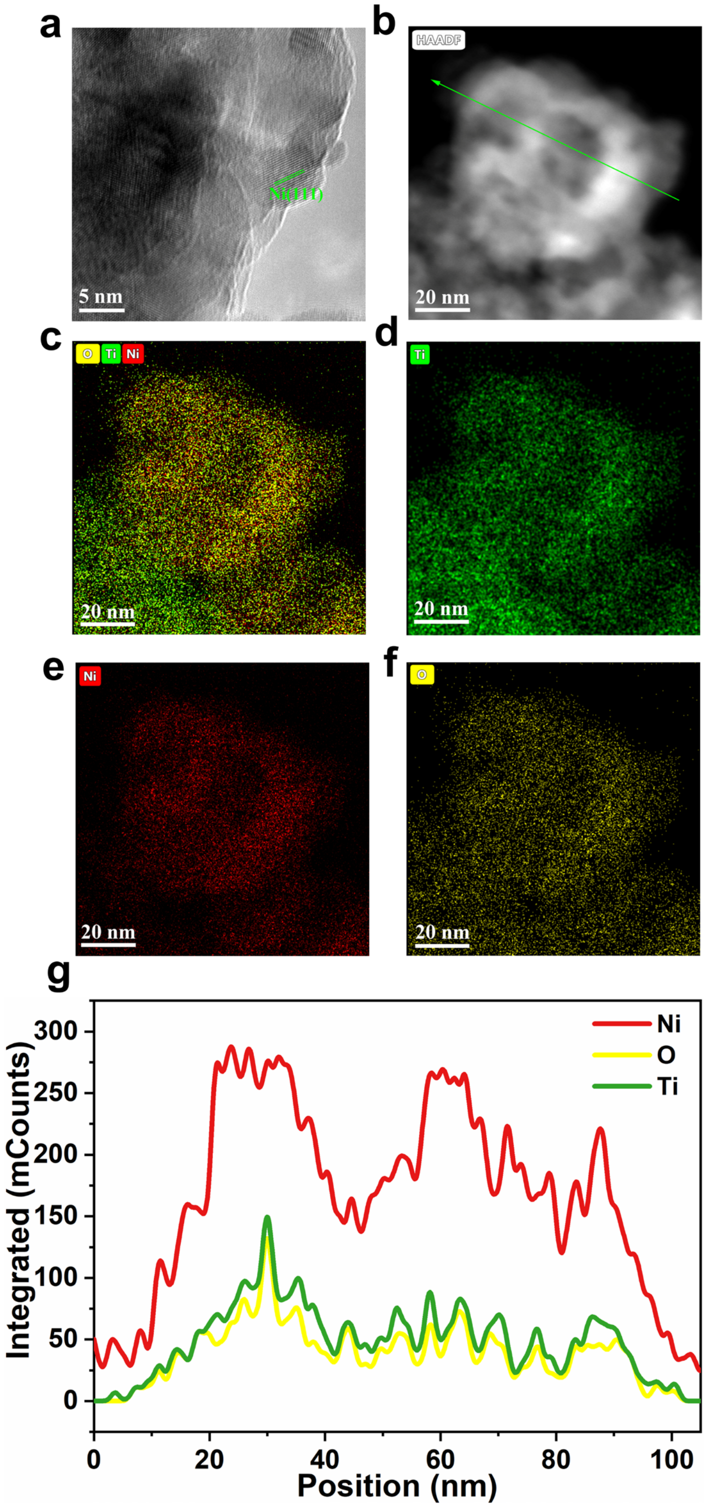

Supplementary Figure 25. Electron micrology studies on the Ni/TiO$_2$. a HR-TEM, **b** ac-HAADF, **c-f** EDS mapping images and **g** line scan signal from **b** for the Ni/TiO$_2$ sample after a cycle of H_2 reduction-O_2 oxidation.

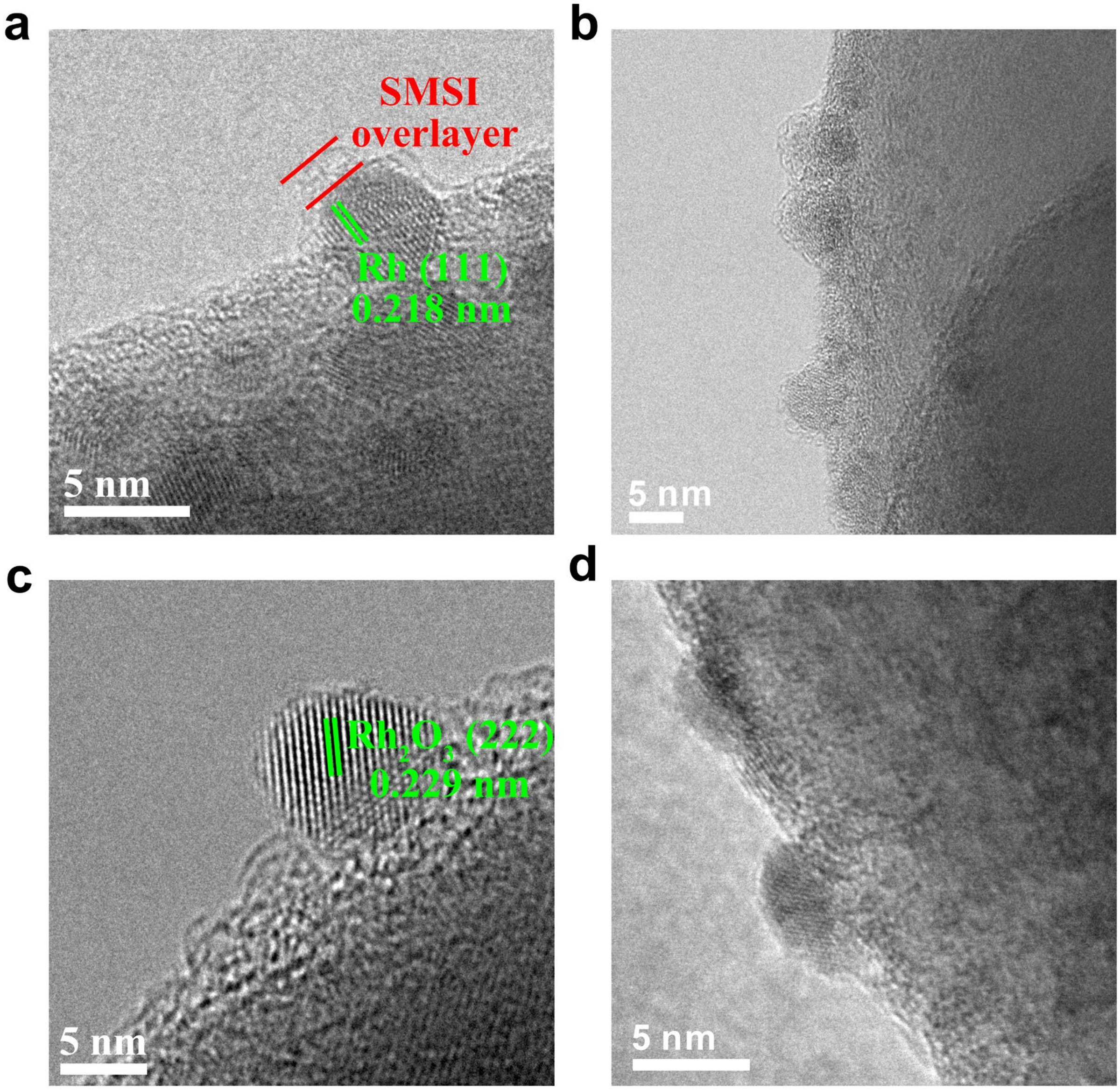

Supplementary Figure 26. Electron micrology studies on the Rh/TiO_2. HR-TEM images of Rh/TiO_2 catalyst after **a, b** H_2 reduction and then **c, d** O_2 treatment.

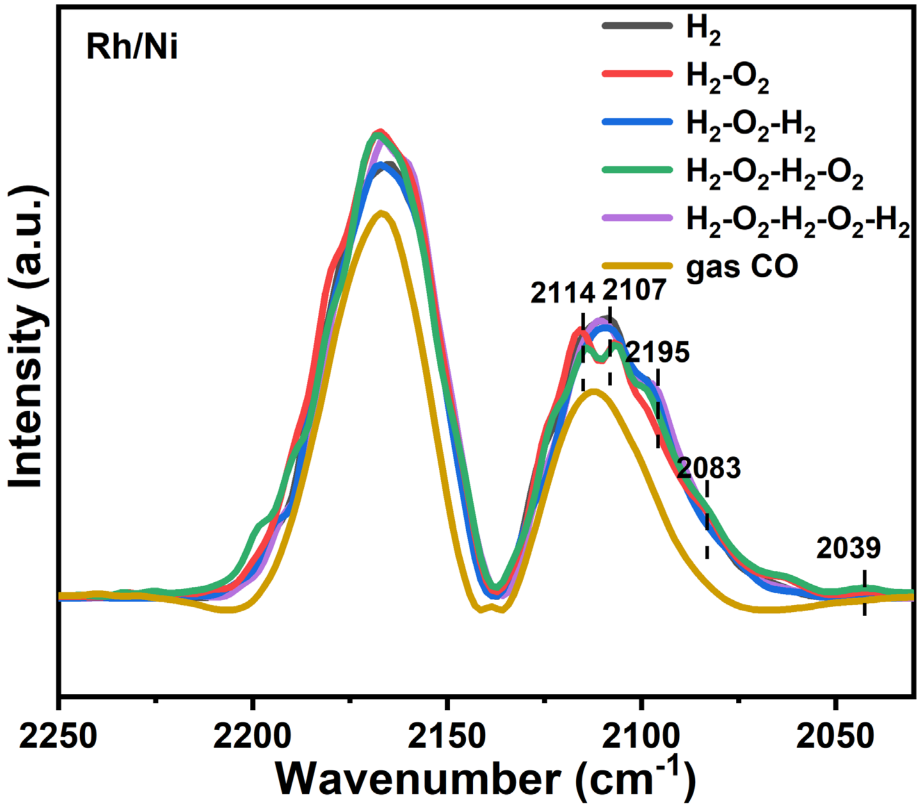

Supplementary Figure 27. CO-DRIFT spectra of Rh/Ni sample. CO-DRIFT spectra for Rh/Ni catalyst at ambiance temperature.

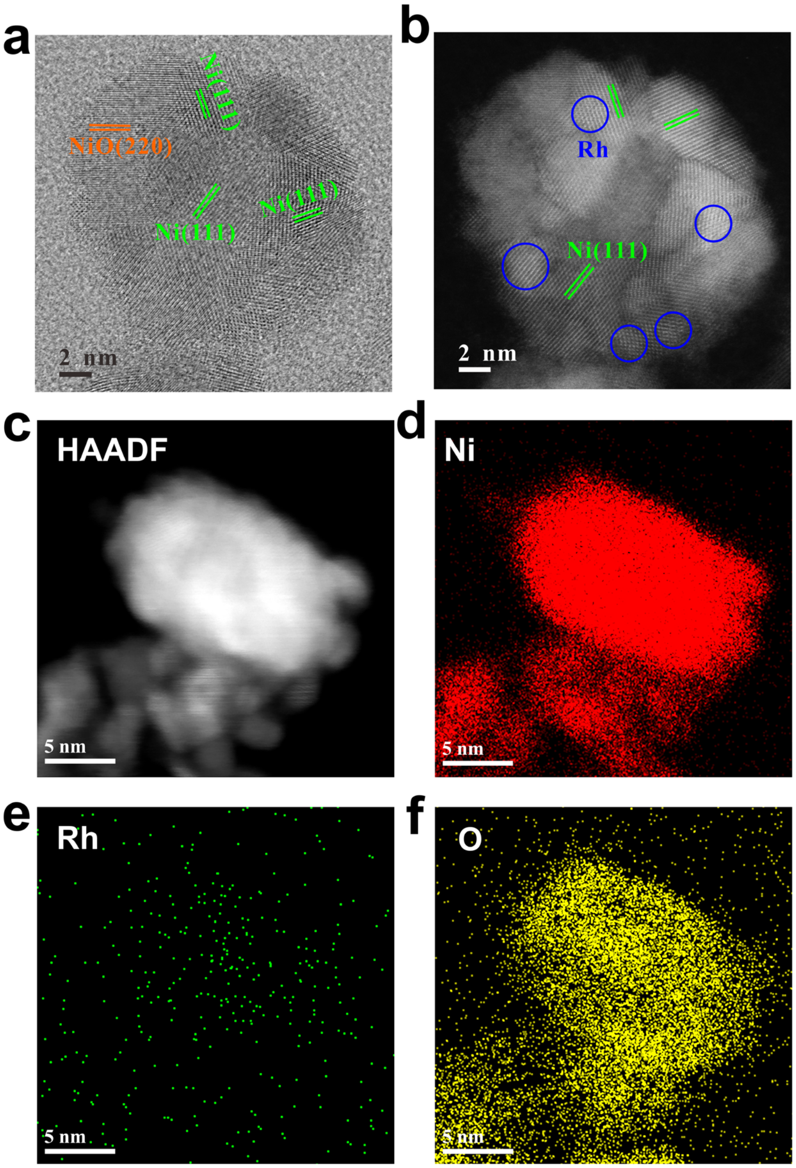

Supplementary Figure 28. Electron micrology studies on the Rh/Ni. a STEM-BF, **b, c** HAADF-STEM and **d-f** EDS mapping images of Rh/Ni catalyst after H_2 reduction.

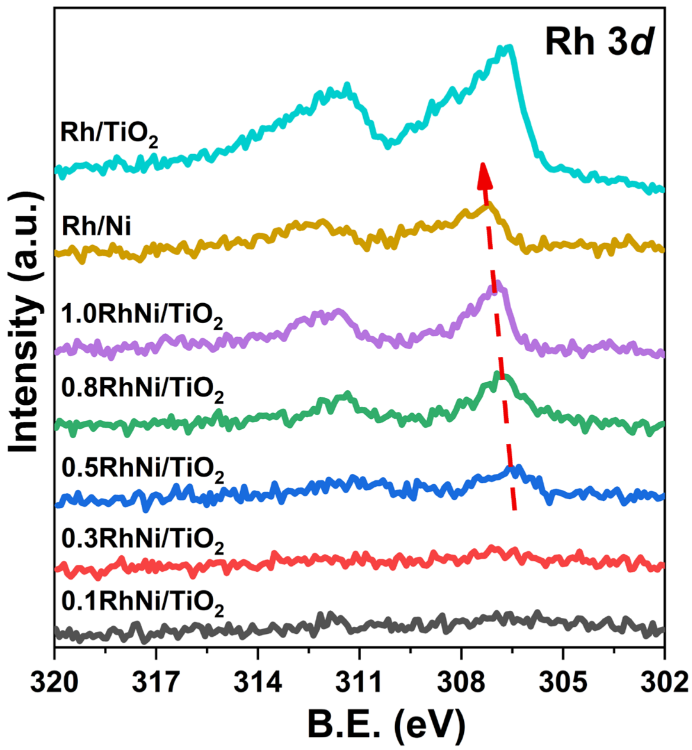

Supplementary Figure 29. XPS spectra of various samples. XPS spectra of Rh 3*d* for various samples.

Supplementary Note 6

XPS spectra of Ni 2*p* for these samples display two binding energies at 852.3–852.8 eV and 855.5–855.8 eV assigned to Ni^0 and Ni^{2+} species, respectively (Fig. 3d), and the one at 861.0–861.2 eV is ascribed to the satellite peak[11-13]. With the increase of Rh content, the electron-binding energy of Ni^0 increases firstly and then decreases; this volcano-type change trend indicates the RhNi bimetal interactions break through the traditional electron transfer mode between single metal and oxide support. XPS spectra of Ti 2*p* were used to probe the Ti species on catalysts surface (Fig. 3e). The deconvoluted peak located at lower binding energy (457.6–457.8 eV) is attributed to the Ti^{3+} species, and the one at higher binding energy (458.7–458.8 eV) is assigned to Ti^{4+} species[14,15]. This indicates that partial Ti^{4+} species at the interface acquires electrons from oxygen vacancies to produce Ti^{3+}. As shown in Supplementary Table 3, compared with Rh/TiO_2, the higher

ratio of $Ti^{3+}/(Ti^{3+}+Ti^{4+})$ on xRhNi/TiO_2 samples suggests a stronger bimetal-support interaction in the latter case (RhNi-TiO_2 interaction). With the increase of Rh content, the relative concentration of Ti^{3+} declines gradually for xRhNi/TiO_2 samples, demonstrating the regulation effects from bimetal-support interaction (SBMSI) in electron transfer. In addition, XPS spectroscopy was used to probe the oxygen species on catalysts surface. As shown in Fig. 3f, the O 1*s* spectrum is deconvoluted into three peaks (529.4–529.7, 530.9–531.2 and 532.3–532.5 eV), which are attributed to the lattice oxygen O^{2-} (denoted as O_α), chemisorbed oxygen O_2^- or O^- (denoted as O_β) and other oxygen species including adsorbed water (denoted as O_γ), respectively[16,17]. The deconvoluted results on the $O_\beta/(O_\alpha + O_\beta + O_\gamma)$ ratio for xRhNi/TiO_2 samples were listed in Supplementary Table 3. The volcano-type change trend of O_β ratio along with Rh loading is associated with the charged state of metal Ni, in which the highest O_β relative content corresponds to the lowest Ni electron density on 0.5RhNi/TiO_2. The electronic state of Rh species was studied through XPS spectra of Rh 3*d*. As shown in Supplementary Fig. 29, all these samples exhibit a lower binding energy of Rh species (306.5–307 eV) compared with Rh^0 (307.2 eV) and Rh^{3+} (308.4 eV), indicating that the Rh exists with an electron-rich state based on electron transfer from Ni to Rh[18-20]. Moreover, the binding energy of Rh species shifts to higher energy with the increase of Rh loading from 0.5% to 1.0% for xRhNi/TiO_2 samples. The results indicate that Rh acquires electrons from Ni species, and the degree of electron transfer declines with the increment of Rh loading. Combining with the results from STEM analysis (Fig. 2 and Supplementary Figs. 18-23), it is proposed that the attenuation of electronic interaction between Rh and Ni is ascribed to the dispersion state of Rh from atomic level to cluster or particle aggregation. Thus, the SBMSI in xRhNi/TiO_2 samples can be well-regulated through changing the Rh loading.

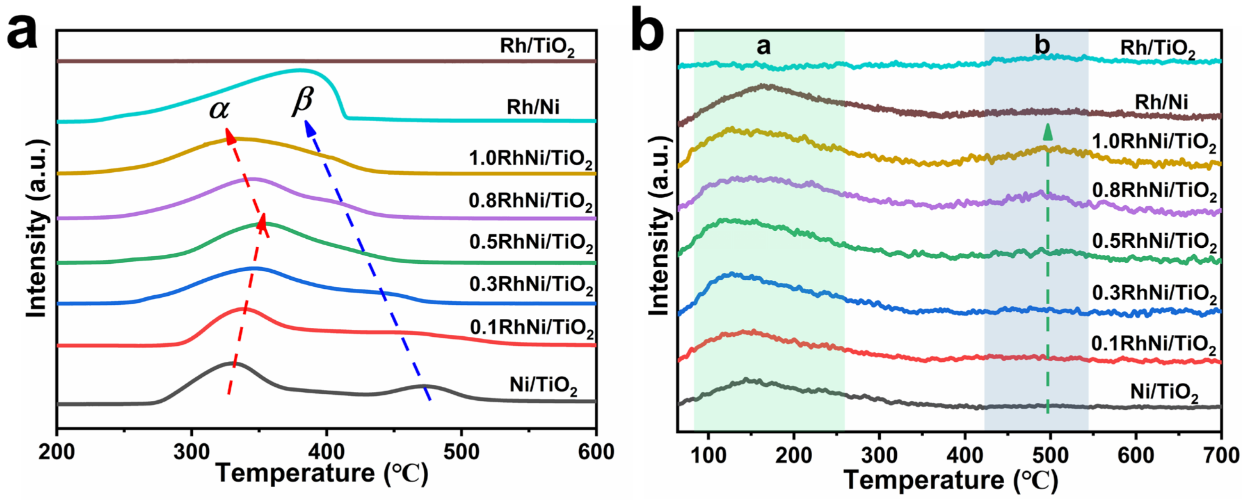

Supplementary Figure 30. H_2-TPR and H_2-TPD experiments. a H_2-TPR and **b** H_2-TPD profiles for various samples.

Supplementary Note 7

To elucidate the SBMSI effect, the reducibility of the calcined samples was studied by H_2-TPR and the curves for H_2 consumption were shown in Supplementary Fig. 30a. For *x*RhNi/TiO_2 samples, the lower temperature reduction peak (α: 300–400 °C) is attributed to the segregated bulk NiO particles that is far away from the NiO-TiO_2 interface. The higher temperature reduction peak (β: 400–500 °C) is assigned to the reduction of NiO located at the Ni-TiO_2 interface with stronger MSI interaction[21]. As the Rh loading increases from 0 to 0.5%, the low temperature reduction peak (α) moves to higher temperature, while the high temperature reduction peak (β) shifts to lower temperature. When Rh content further increases from 0.5 % to 1.0 %, the reduction peaks of both α and β move to low temperature at the same time. According to the H_2-TPD results (Supplementary Fig. 30b), the H_2 desorption peak (a: 100–200 °C) is ascribed to the H-species adsorbed on the Ni surface[22]. Both the shape and peak area of this peak are almost identical for these *x*RhNi/TiO_2 samples, in line with their similar Ni content and particle size. In addition, a weak H_2 desorption peak (b: 450-500 °C), which is attributed to the H anion species ($H^{\delta-}$) held at

oxygen vacancy at Ni-TiO_2 interface due to hydrogen overflow effect[23]. Therefore, the introduction of Rh with atomic-level dispersion promotes H_2 spillover and thus facilitates the reduction of NiO species located at the interface. With the increase of Rh content (0.5−1.0 *wt.*%), the hydrogen spillover effect enhances due to the appearance of Rh ensemble, which further boosts the reduction of segregated bulk NiO species, in accordance with the results of H_2-TPR (Supplementary Fig. 30a). The results above demonstrate an intense interaction between RhNi bimetallic particles and TiO_2 support.

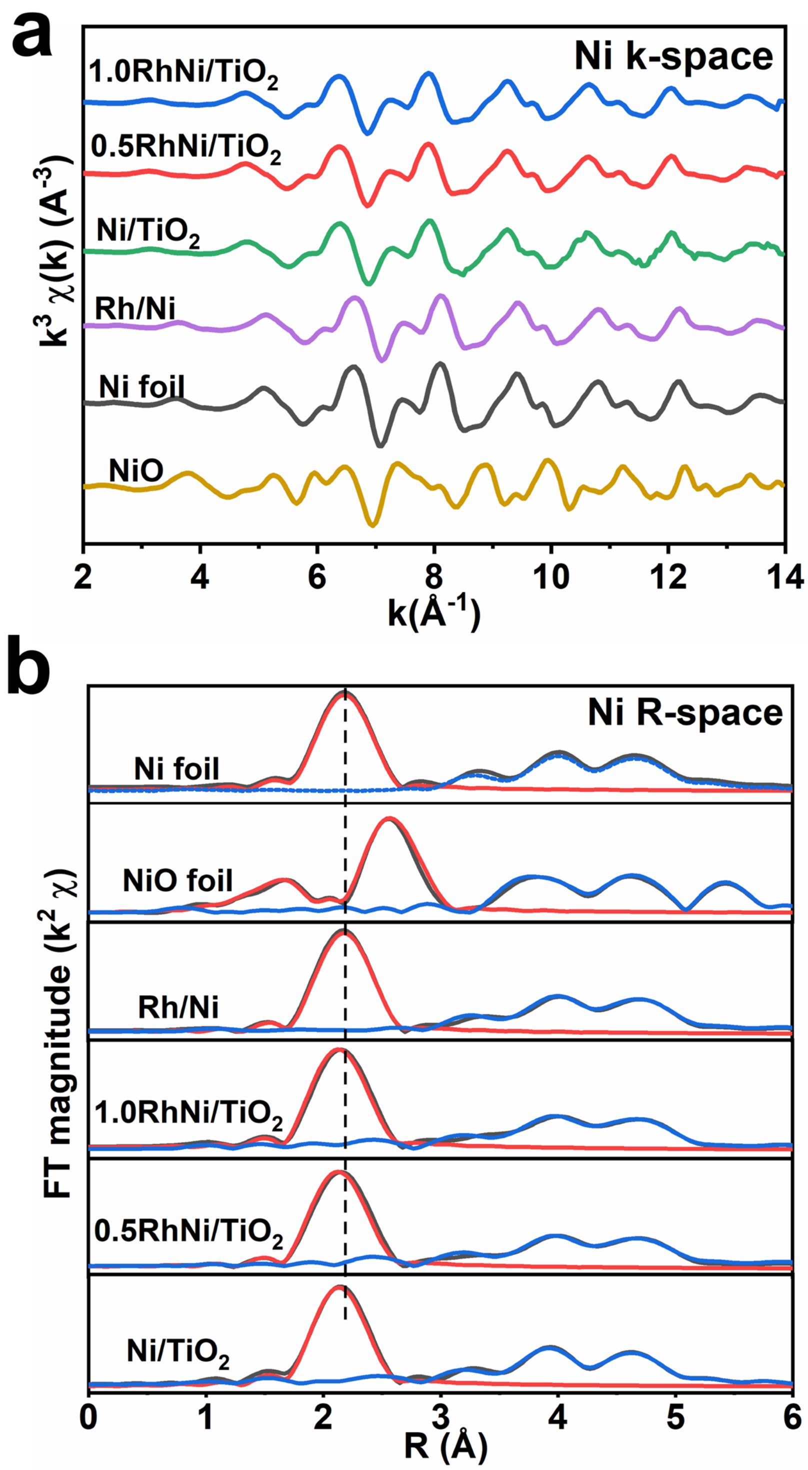

Supplementary Figure 31. Fine-structure characterization of various samples. Fourier transform of Ni K-edge **a** EXAFS spectra in k-space and **b** fitting results in R-space for various samples (the black line: experimental data; the red and blue line: fitting curves).

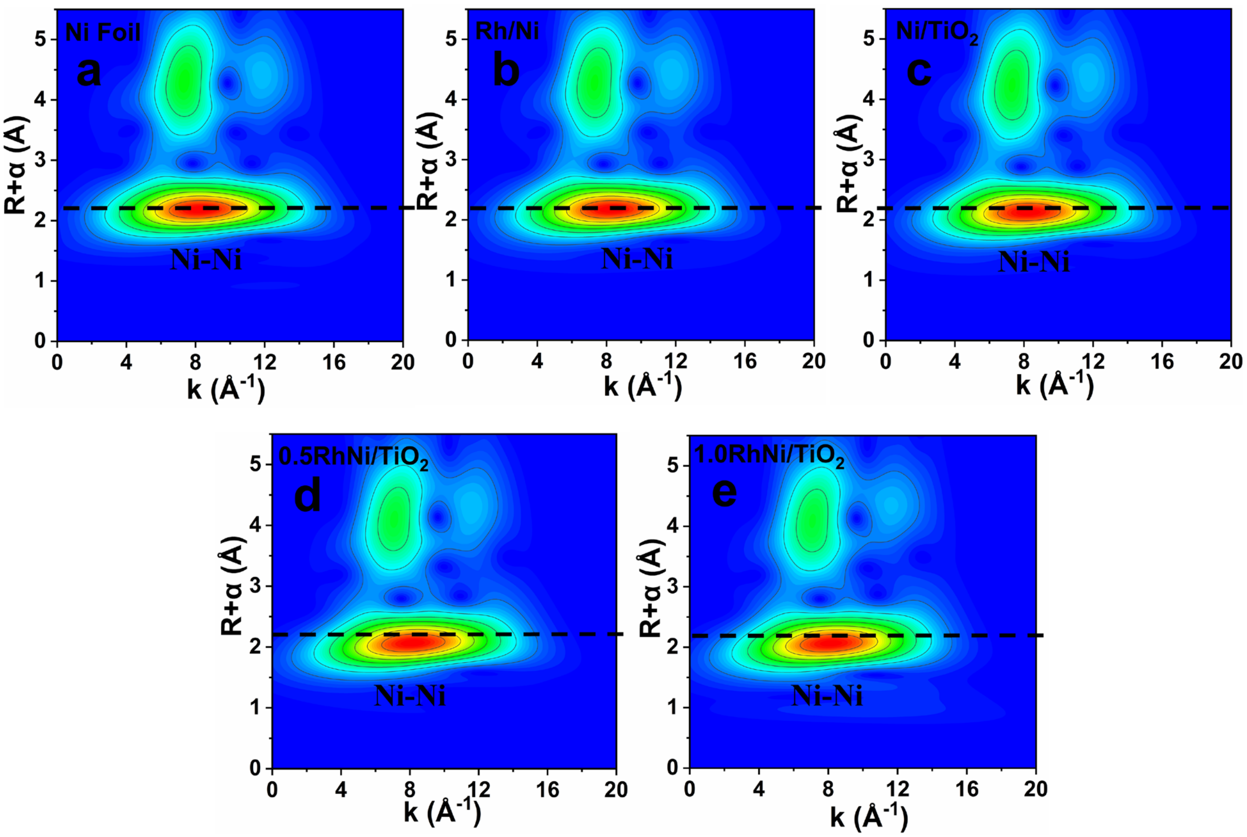

Supplementary Figure 32. Fine-structure characterization of various samples. k^3 weighted wavelet transforms for the Ni K-edge XAFS signals of **a** Ni foil, **b** Rh/Ni, **c** Ni/TiO_2, **d** 0.5RhNi/TiO_2 and **e** 1.0RhNi/TiO_2 sample based on Morlet wavelets.

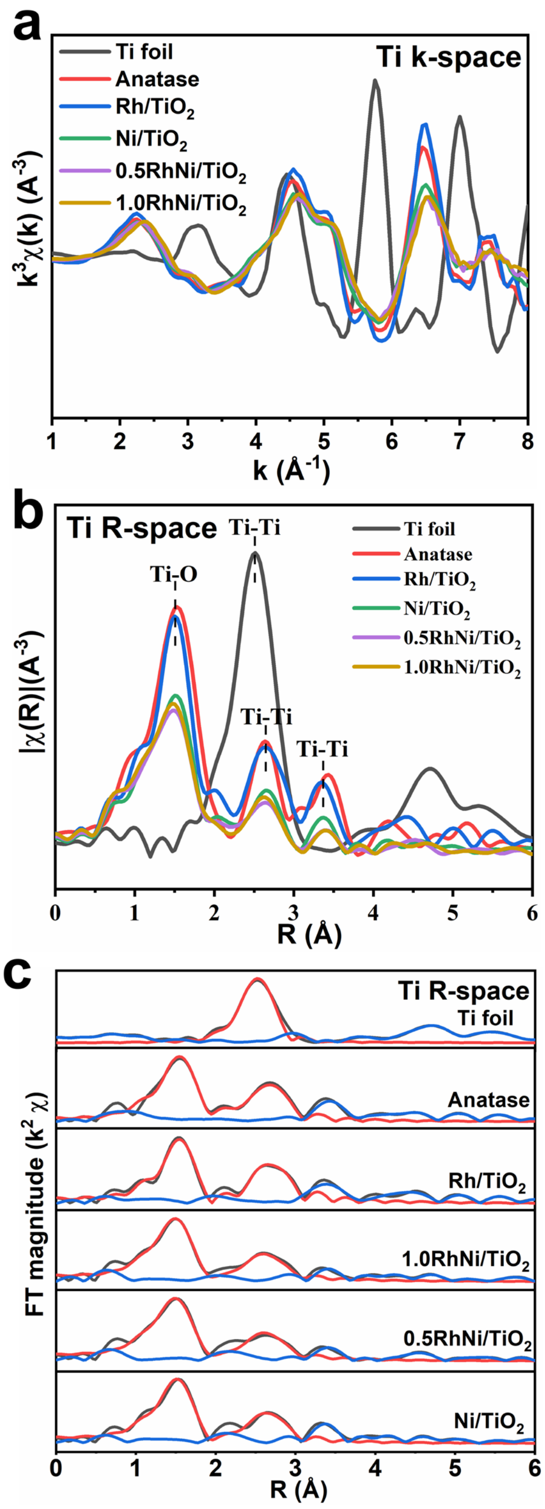

Supplementary Figure 33. Fine-structure characterization of various samples. Fourier transform of Ti K-edge EXAFS spectra in **a** k-space, **b** R-space and **c** fitting results for various samples (the black line: experimental data; the red and blue line: fitting curves).

Supplementary Note 8

The results of Fourier transform of Ti K-edge EXAFS spectra for various samples are shown in Supplementary Fig. 33. The strong peak at 1−2 Å (not phase corrected) in R-space (Supplementary Fig. 33b) corresponds to the first coordination shell of anatase (Ti−O). The other two peaks located between 2 and 4 Å (not phase corrected) are assigned to Ti−Ti bonds from four Ti atoms in the second shell. The Ti foil exhibits a peak situated within 2−3 Å (not phase corrected) ascribed to the Ti−Ti bond. The fitting results show that the first shell of Ti−O bond in *x*RhNi/TiO_2 samples gives a coordination number less than a sixfold coordination relative to anatase (Supplementary Fig. 33c and Supplementary Table 5), which is related to the existence of distorted octahedral environment from the bimetal-support interaction[24].

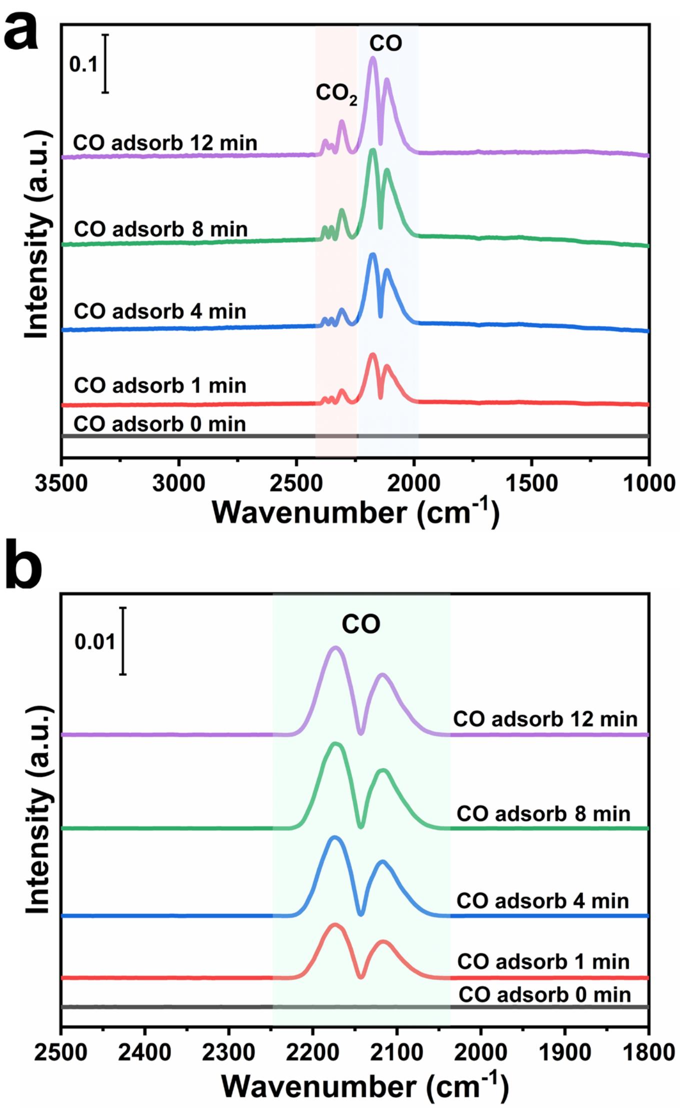

Supplementary Figure 34. *In situ* CO-DRIFT characterization of 0.5RhNi/TiO_2. *In situ* DRIFT spectra of CO adsorption on 0.5RhNi/TiO_2 catalyst at **a** 400 °C and **b** 200 °C, respectively.

Supplementary Note 9

As shown in Supplementary Fig. 34a, a rapid emergence of CO_2 signals is observed once CO is introducted into the reactor at 400 °C, indicating the occurrence of CO disproportionation in the presence of 0.5RhNi/TiO_2 catalyst. We further explored the *in situ* DRIFT spectra of CO adsorption on 0.5RhNi/TiO_2 catalyst at 200 °C under similar experiment conditions (Supplementary Fig. 34b). The results show that CO does not undergo disproportionation reaction at a lower temperature.

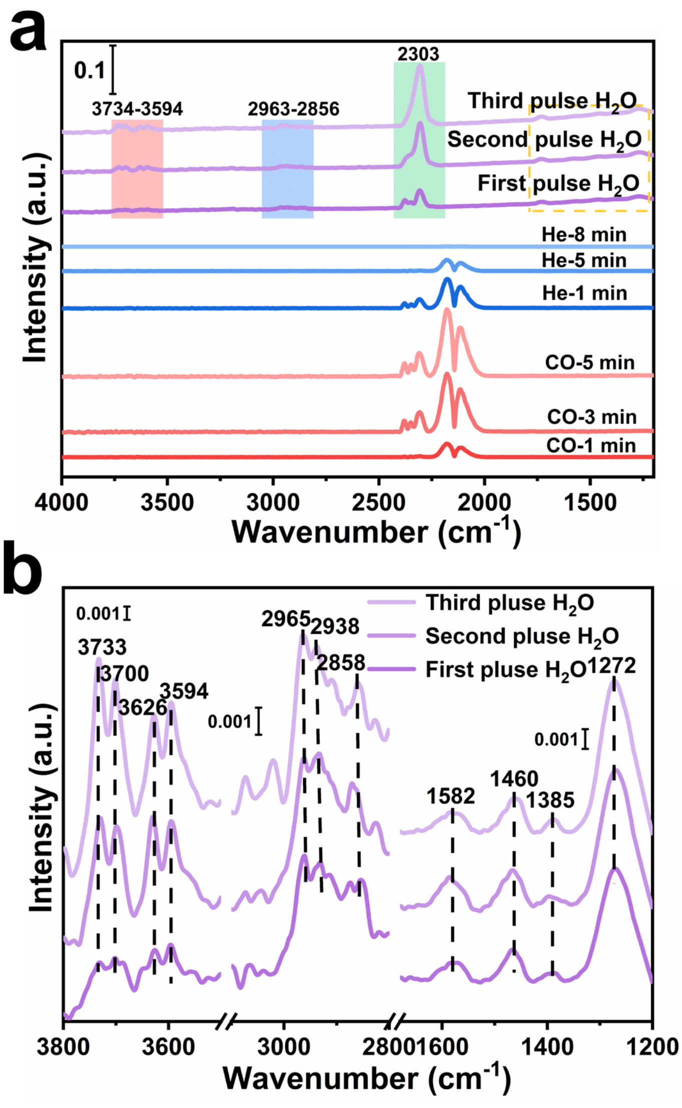

Supplementary Figure 35. *In situ* DRIFT spectra of 0.5RhNi/TiO$_2$. a *In situ* DRIFT spectra for a successive measurement including CO adsorption, He flushing and subsequent H_2O pulse over 0.5RhNi/TiO$_2$ catalyst at 400 °C. **b** Local magnification regions in **a**.

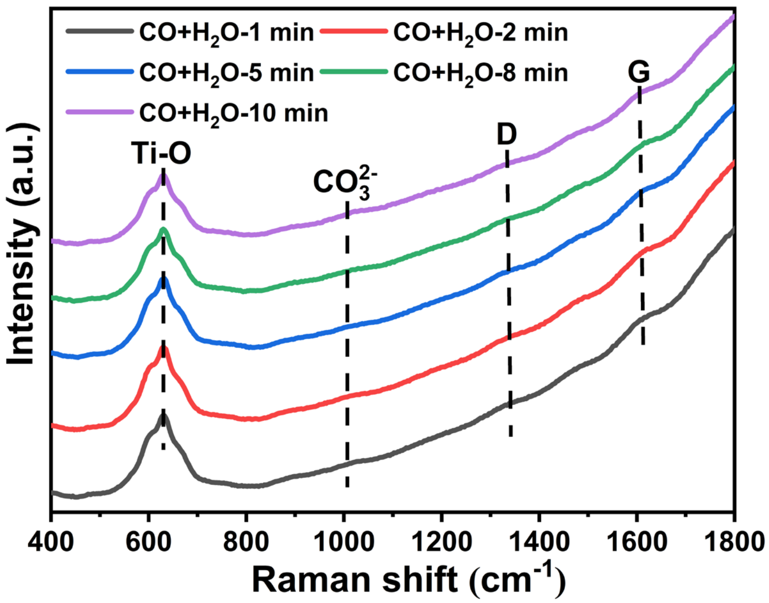

Supplementary Figure 36. *In situ* DRIFT Raman of 0.5RhNi/TiO₂. *In situ* Raman spectra for co-adsorption of CO and saturated H_2O steam over 0.5RhNi/TiO_2 catalyst at 400 °C.

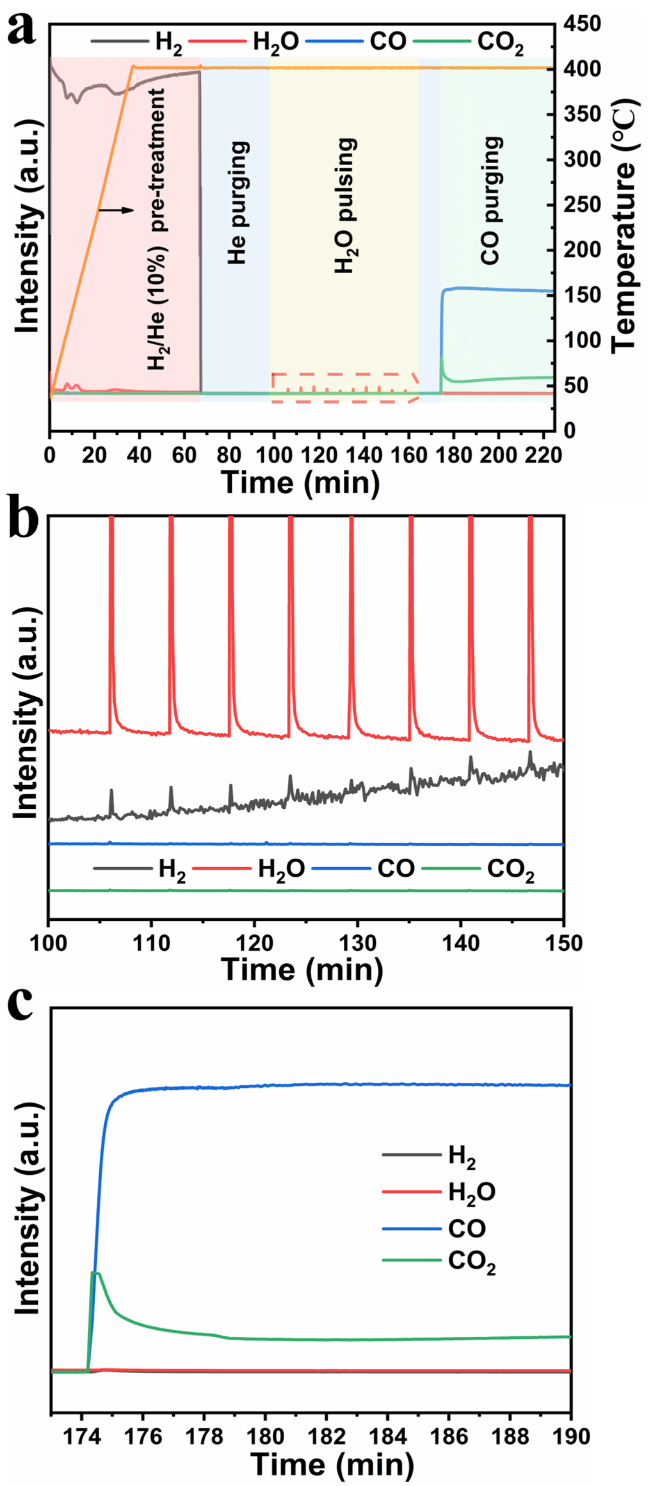

Supplementary Figure 37. Mass spectral analysis of Rh/Ni. Mass spectral analysis for CO and H_2O pulse over **a** Rh/Ni catalyst. **b**, **c** corresponding local magnification regions in **a**.

Supplementary Note 10

The Rh/Ni catalyst was pretreated with similar gas condition as executed on 0.5RhNi/TiO_2 catalyst (Fig. 4g-i). Then, the gas was switched to saturated water vapor, He and CO in turn to monitor the gas signals (Supplementary Figs. 37a-c). From Supplementary Fig. 37b, H_2 signal was immediately detected after a saturated water vapor pulse, indicating the presence of water dissociation center on Rh/Ni. Subsequently, a switching to CO led to the appearance of CO_2 (Supplementary Fig. 37c), which was attributed to the consumption of surface oxygen species derived from water dissociation. The results indicate that the steam reforming reaction obeys a redox mechanism in the presence of Rh/Ni catalyst.

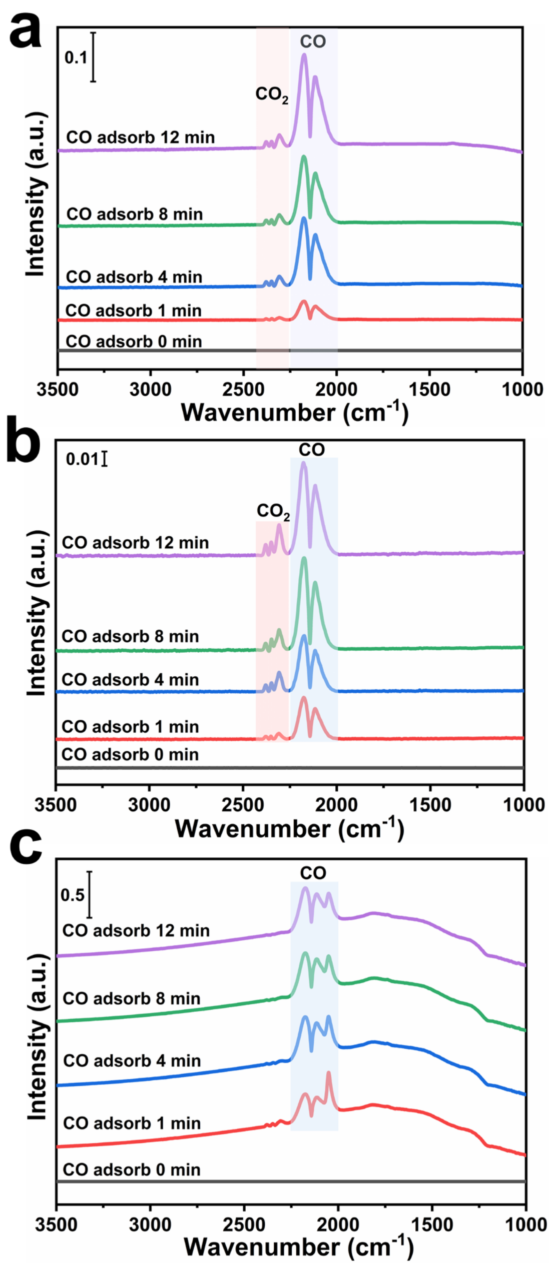

Supplementary Figure 38. *In situ* CO-DRIFT characterization of various samples. *In situ* DRIFT spectra of CO adsorption on **a** Ni/TiO_2, **b** Rh/Ni and **c** Rh/TiO_2 catalysts at 400 °C, respectively.

Supplementary Note 11

For the Ni/TiO_2 and Rh/Ni catalysts (Supplementary Figs. 38a and 38b), a similar phenomenon about CO disproportionation is observed in both cases, but it becomes weaker on

Ni/TiO_2 relative to Rh/Ni and 0.5RhNi/TiO_2 catalysts. In contrast, the CO disproportionation does not happen on Rh/TiO_2 (Supplementary Fig. 38c). Therefore, the Ni sites play a significant role in CO disproportionation, and RhNi-bimetal structure promotes this process.

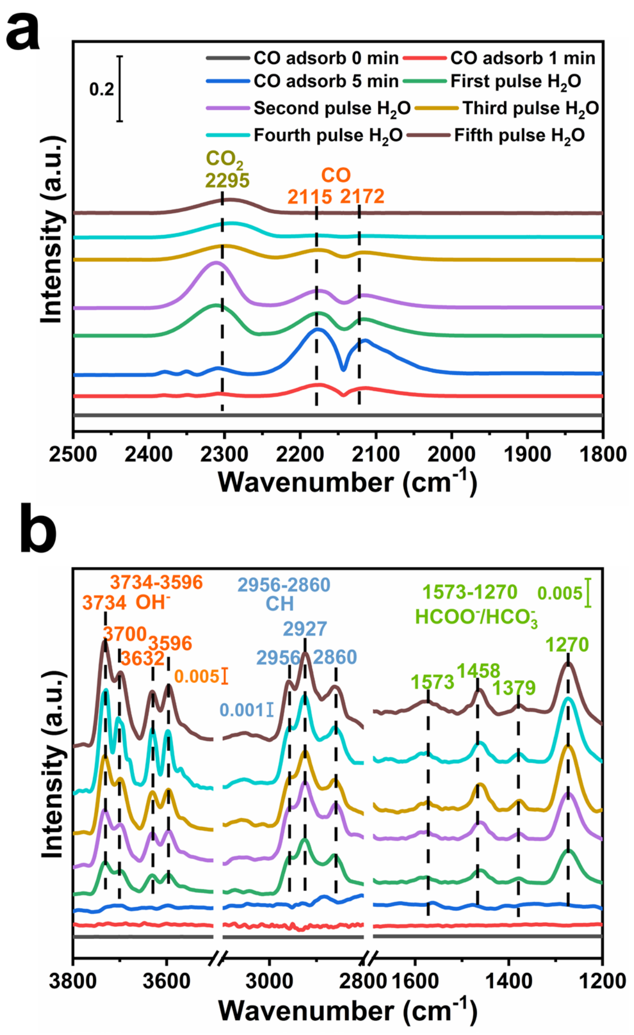

Supplementary Figure 39. *In situ* DRIFT spectra of Ni/TiO₂. a *In situ* DRIFT spectra for a successive measurement including CO adsorption and subsequent H_2O pulse on Ni/TiO_2 catalyst at 400 °C. **b** Local magnification regions in **a**.

Supplementary Note 12

For the Ni/TiO_2 catalyst with $Ni\text{-}TiO_2$ SMSI, a similar associative reaction mechanism to the $0.5RhNi/TiO_2$ catalyst was revealed through *in situ* DRIFTS analysis (Supplementary Fig. 39). However, a weaker CO disproportionation as well as a lower concentration of formate intermediate were observed, in comparison with $0.5RhNi/TiO_2$ catalyst (Fig. 4a and b), demonstrating the advantages of SBMSI in $0.5RhNi/TiO_2$ for WGS reaction.

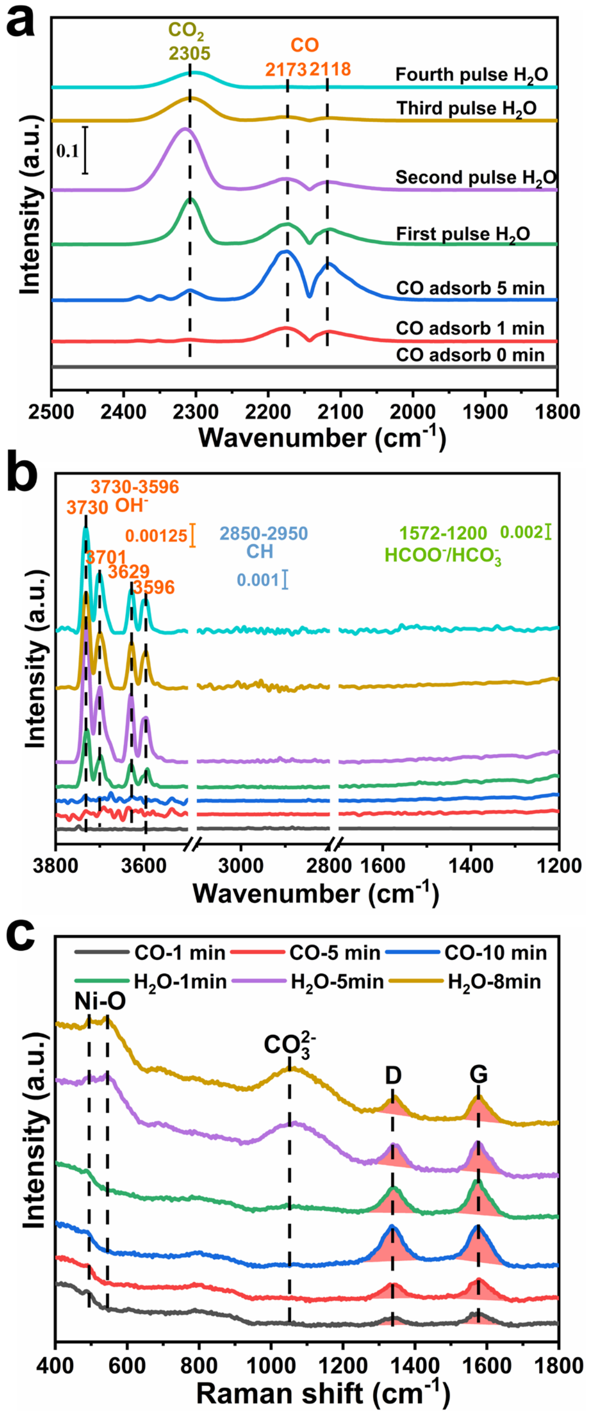

Supplementary Figure 40. *In situ* DRIFT and Raman spectra of Rh/Ni. a *In situ* DRIFT spectra for a successive measurement including CO adsorption and H_2O pulse on Rh/Ni catalyst at 400 °C. **b** Local magnification regions in **a**. **c** *In situ* Raman spectra for a successive measurement including CO adsorption and subsequent saturated H_2O steam adsorption over Rh/Ni catalyst at 400 °C.

Supplementary Note 13

For the Rh/Ni catalyst with the absence of SBMSI, the appearance of CO_2 and C species after CO adsorption indicates the occurrence of CO disproportionation reaction as demonstrated by *in situ* DRIFTS and *in situ* Raman analysis (Supplementary Fig. 40). Subsequently, a decrease in Raman signal of C specie was observed after introduction of H_2O (Supplementary Fig. 40c), but no remarkable reaction intermediate was detected in *in situ* DRIFTS results (Supplementary Fig. 40b). Meanwhile, a very small quantity of oxhydryl species (3730–3596 cm^{-1}) after H_2O pulse was found on the Rh/Ni catalyst compared with Ni/TiO_2 and $0.5RhNi/TiO_2$ (Fig. 4b, Supplementary Figs. 39b and 40b), indicating that the hydroxyl component is not stable on Ni sites relative to the oxygen vacancy of TiO_2. Furthermore, the mass spectral analysis displays an obvious H_2 signal after a saturated water vapor pulse at 400 °C (Supplementary Fig. 37e). The results confirm that the WGS reaction follows redox mechanism rather than associative mechanism in the presence of Rh/Ni catalyst. In addition, compared with the $0.5RhNi/TiO_2$ catalyst (Fig. 4c), a remarkable Raman shift at 1060 cm^{-1} assigned to carbonate species is observed on Rh/Ni (Supplementary Fig. 40c), corresponding to the strong poisoning effect imposed by CO_2 on this catalyst.

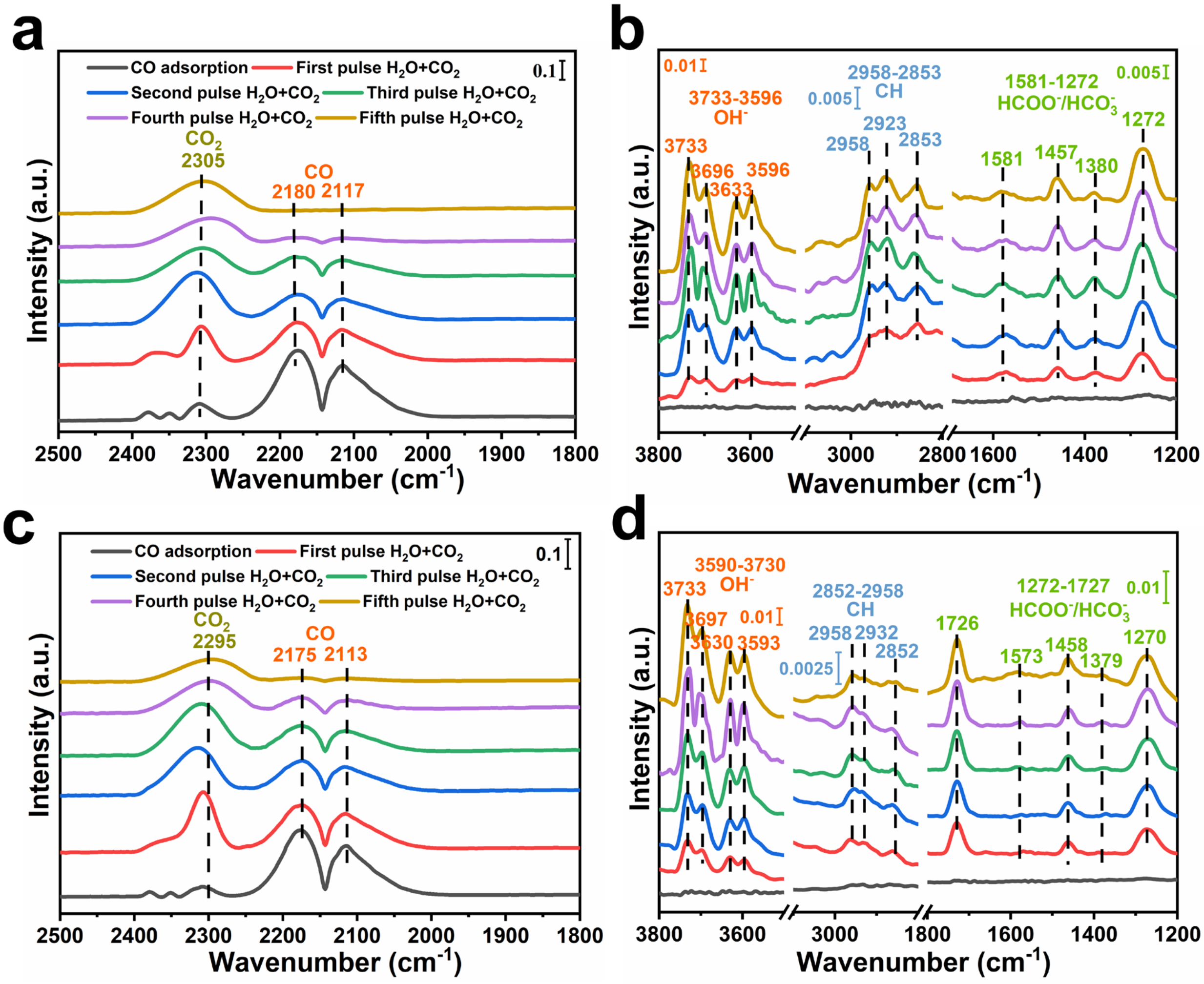

Supplementary Figure 41. ***In situ*** **DRIFT spectra of 0.5RhNi/TiO2 and Ni/TiO2.** *In situ* DRIFT spectra for a successive measurement including CO adsorption and subsequent H_2O + CO_2 pulse on **a**, **b** 0.5RhNi/TiO_2 and **c**, **d** Ni/TiO_2 catalyst at 400 °C.

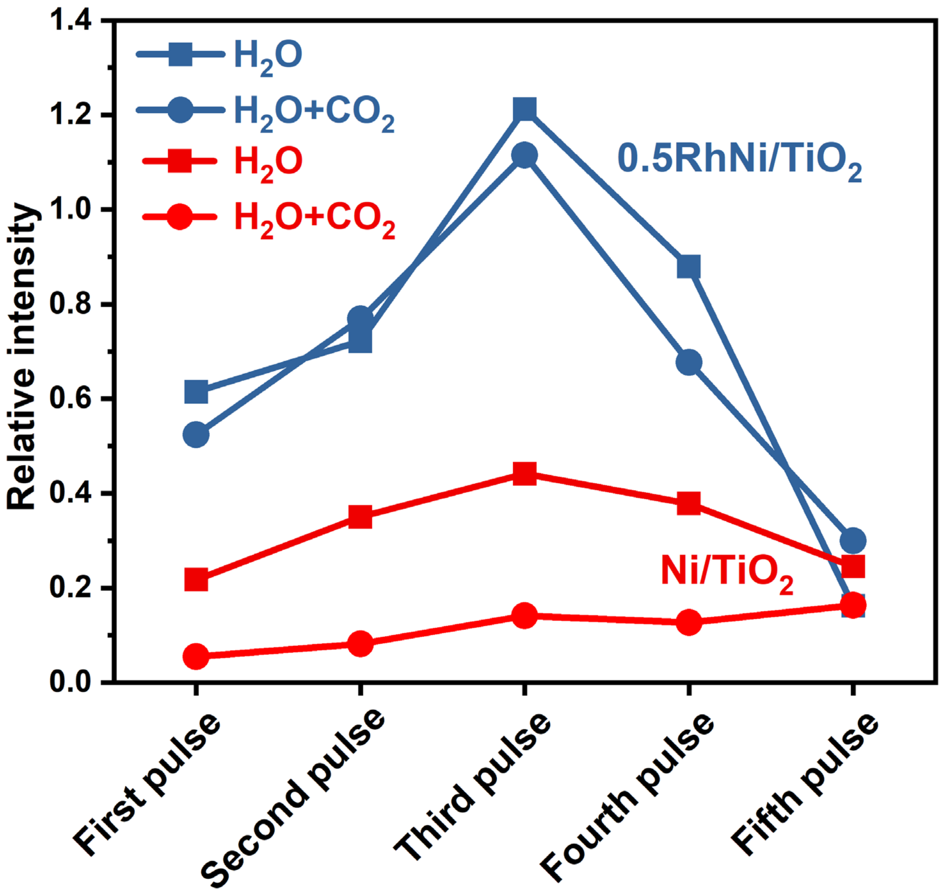

Supplementary Figure 42. Statistical results of *in situ* DRIFT spectra. Relative intensity of *v*(HCOO⁻) from H_2O or $H_2O + CO_2$ pulse shown in Fig. 3B, figs. S34b, S36b and S36d, which is calculated based on the intensity ratio of *v*(HCOO⁻, 1379 cm^{-1})/*v*(HCO_3^-, 1457 cm^{-1}) in the presence of 0.5RhNi/TiO_2 and Ni/TiO_2 catalysts, respectively.

Supplementary Note 14

CO_2 and H_2O were pulsed together in the presence of 0.5RhNi/TiO_2, Ni/TiO_2 and Rh/Ni catalysts after CO adsorption, in order to study the influence of CO_2 on steam reforming reaction. As shown in Supplementary Fig. 41, in comparison with 0.5RhNi/TiO_2, a more obvious inhibitory effect for $HCOO^-$ intermediate (1573, 1379 and 2852–2958 cm^{-1}) formation on Ni/TiO_2 sample was observed. Furthermore, the relative intensity of *v*(HCOO⁻) vibration was calculated based on the intensity ratio of *v*(HCOO⁻, 1380 cm^{-1})/*v*(HCO_3^-, 1457 cm^{-1}) after H_2O or $H_2O + CO_2$ pulse

(Supplementary Fig. 42). Relative to 0.5RhNi/TiO_2 catalyst, the formation and transformation of formate on Ni/TiO_2 were significantly inhibited due to the introduction of CO_2, corresponding to the lower CO conversion in the latter case. This demonstrates the advantages of SBMSI in 0.5RhNi/TiO_2 over SMSI in Ni/TiO_2.

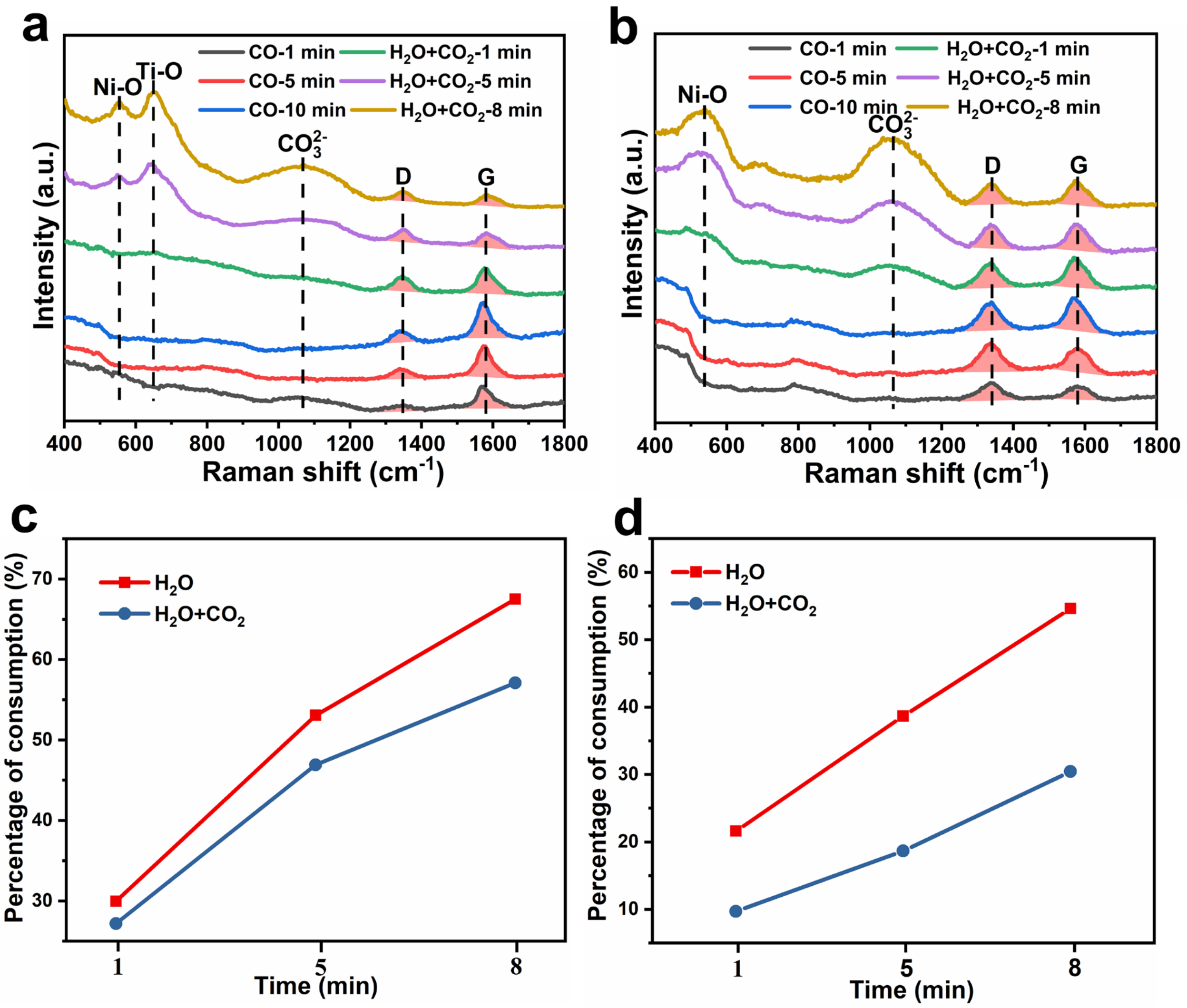

Supplementary Figure 43. *In situ* Raman spectra and corresponding statistical results of 0.5RhNi/TiO2 and Rh/Ni. *In situ* Raman spectra for a successive measurement including CO adsorption and subsequent H_2O+CO_2 adsorption over **a** 0.5RhNi/TiO_2 and **b** Rh/Ni catalyst at 400 °C. Consumption percentage of surface C species from D+G peak area for **c** 0.5RhNi/TiO_2 and **d** Rh/Ni catalyst, respectively.

Supplementary Note 15

In situ Raman spectrscopy was used to explore the influence of CO_2 on CO steam reforming reaction over 0.5RhNi/TiO_2 and Rh/Ni catalysts. As shown in Supplementary Fig. 43a and 43b, the CO_3^{2-} signal over Rh/Ni sample shows a stronger intensity relative to 0.5RhNi/TiO_2 after the

introduction of H_2O + CO_2; moreover, the consumption rate of surface C species declines more significantly in the former case (Supplementary Figs. 43c and 43d), which is consistent with the poor CO conversion over Rh/Ni sample. The strong inhibition effect of Rh/Ni catalyst *via* coordination with carbonate species was also proved by Quasi-*in situ* XPS (Supplementary Fig. 44). Therefore, benefiting from the SBMSI, a facile removal of CO_2 or carbonate from the surface of 0.5RhNi/TiO_2 catalyst facilitates the recovery of active sites for the subsequent catalytic cycling.

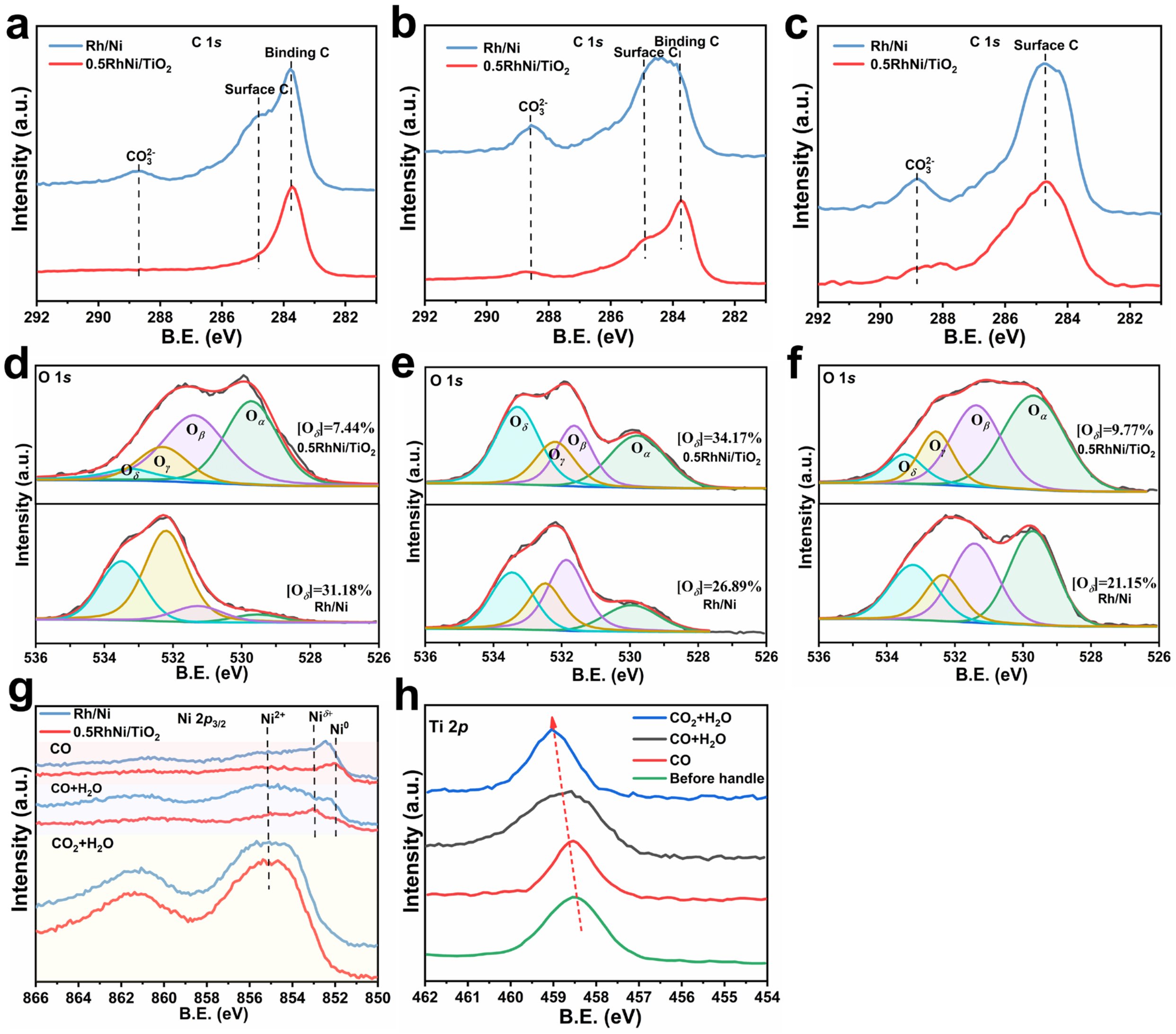

Supplementary Figure 44. Quasi-*in situ* XPS spectra of 0.5RhNi/TiO_2 and Rh/Ni. Quasi-*in situ* XPS spectra of C 1*s* and O 1*s* after **a**, **d** CO, **b**, **e** CO + H_2O, **c**, **f** CO_2 + H_2O treatment on 0.5RhNi/TiO_2 and Rh/Ni at 400 °C, respectively. Quasi-*in situ* XPS spectra of **g** Ni 2*p* and **h** Ti 2*p* after CO, CO+H_2O and CO_2+H_2O adsorption on 0.5RhNi/TiO_2 and Rh/Ni at 400 °C, respectively.

Supplementary Note 16

To further study the discrepancy of surface carbon species on the 0.5RhNi/TiO_2 and Rh/Ni catalysts during the steam reforming reaction, Quasi-*in situ* XPS spectra were carried out. Firstly, we explored the effect from CO adsorption at 400 °C on the catalyst surface. From the results from C 1*s* (Supplementary Fig. 44a), a remarkable binding of C species (283.7 eV) is found from CO

disproportionation on both 0.5RhNi/TiO_2 and Rh/Ni catalysts[25]. In addition, compared with 0.5RhNi/TiO_2, an obvious peak appears at 288−288.5 eV in the case of Rh/Ni catalyst, which is attributed to the surface carbonate specie[25,26]. This is further confirmed by O 1*s* spectra (Supplementary Fig. 44d). After the CO treatment, the O 1*s* curve shows binding energies at 529.4−529.7, 531.2−531.4, 532.2−532.3 and 533.2−533.4 eV, which are assigned to lattice oxygen (O_{α}), chemisorbed oxygen (O_{β}), absorbed water (O_{γ}) and O−C−O species (O_{δ}), respectively[16,17,25]. Obviously, the Rh/Ni catalyst displays a higher content of O_{δ} species compared with 0.5RhNi/TiO_2 catalyst, indicating a more abundant carbonate species from CO disproportionation in the former case. When both samples were treated by CO + H_2O at 400 °C, a more remarkable peak at 288−288.5 eV assigned to carbonate specie was observed from C 1*s* spectrum on Rh/Ni catalyst (Supplementary Fig. 44b). However, an opposite phenomenon was found on O 1*s* spectra, where the 0.5RhNi/TiO_2 showed a stronger signal of O_{δ} species than Rh/Ni catalyst (Supplementary Fig. 44e). Taking into account the associative mechanism on 0.5RhNi/TiO_2 catalyst, the higher O_{δ} species on 0.5RhNi/TiO_2 is mainly derived from formate intermediate. After the treatment of CO_2 + H_2O on 0.5RhNi/TiO_2 and Rh/Ni catalysts, both C 1*s* and O 1*s* spectra showed a lower carbonate and O_{δ} peak intensities (Supplementary Figs. 44c and 44f) on 0.5RhNi/TiO_2 relative to Rh/Ni one. Moreover, no binding C species was produced, indicating that the C species indeed arises from CO disproportionation reaction. The results verify a stronger carbonate binding ability on the Rh/Ni catalyst than that on 0.5RhNi/TiO_2, which is consistent with the results of CO_2-TPD and *in situ* Raman.

As shown in Supplementary Fig. 40g, a larger shift in Ni 2*p* towards higher energy is present on Rh/Ni sample after H_2O treatment, which indicates that Ni as the active site promotes H_2O activation, corresponding to the redox mechanism. As shown in Supplementary Fig. 44h, the Ti

2*p* peak of 0.5RhNi/TiO_2 moves towards higher binding energy after CO, CO + H_2O and CO_2 + H_2O treatment. This demonstrates that TiO_2 inhibits carbonate poisoning on 0.5RhNi/TiO_2 catalyst, which would be further explored in the following study.

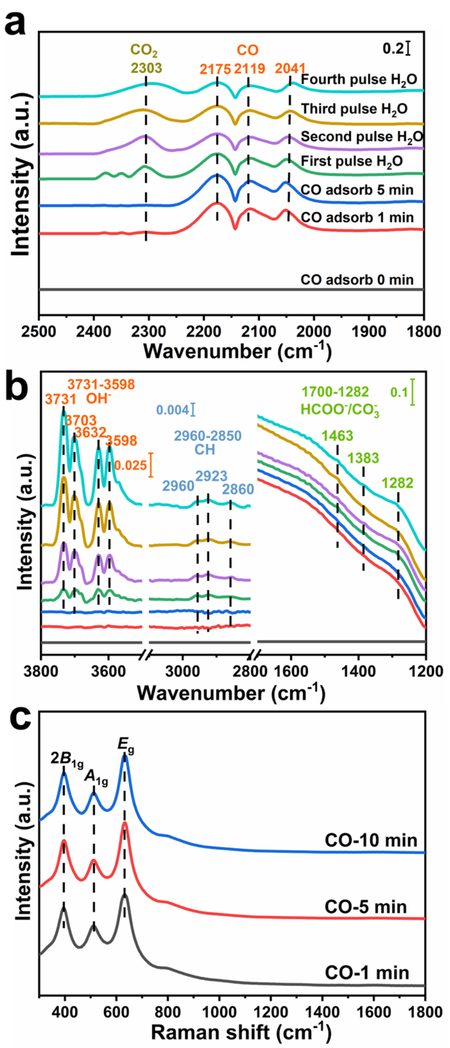

Supplementary Figure 45. *In situ* DRIFT and Raman spectra of Rh/TiO_2. a *In situ* DRIFT spectra for a successive measurement including CO adsorption, He flushing and subsequent H_2O pulse on Rh/TiO_2 catalyst at 400 °C. **b** Local magnification regions within 1200–3800 cm^{-1} in **a**. **c** *In situ* Raman spectra for a successive measurement including CO adsorption over Rh/TiO_2 catalyst at 400 °C.

Supplementary Note 17

In situ DRIFTS and *in situ* Raman measurements were carried out on Rh/TiO_2 catalyst (Supplementary Fig. 45), and no carbon species and other reaction intermediates were detected after CO and H_2O introduction, corresponding to its poor catalytic performance.

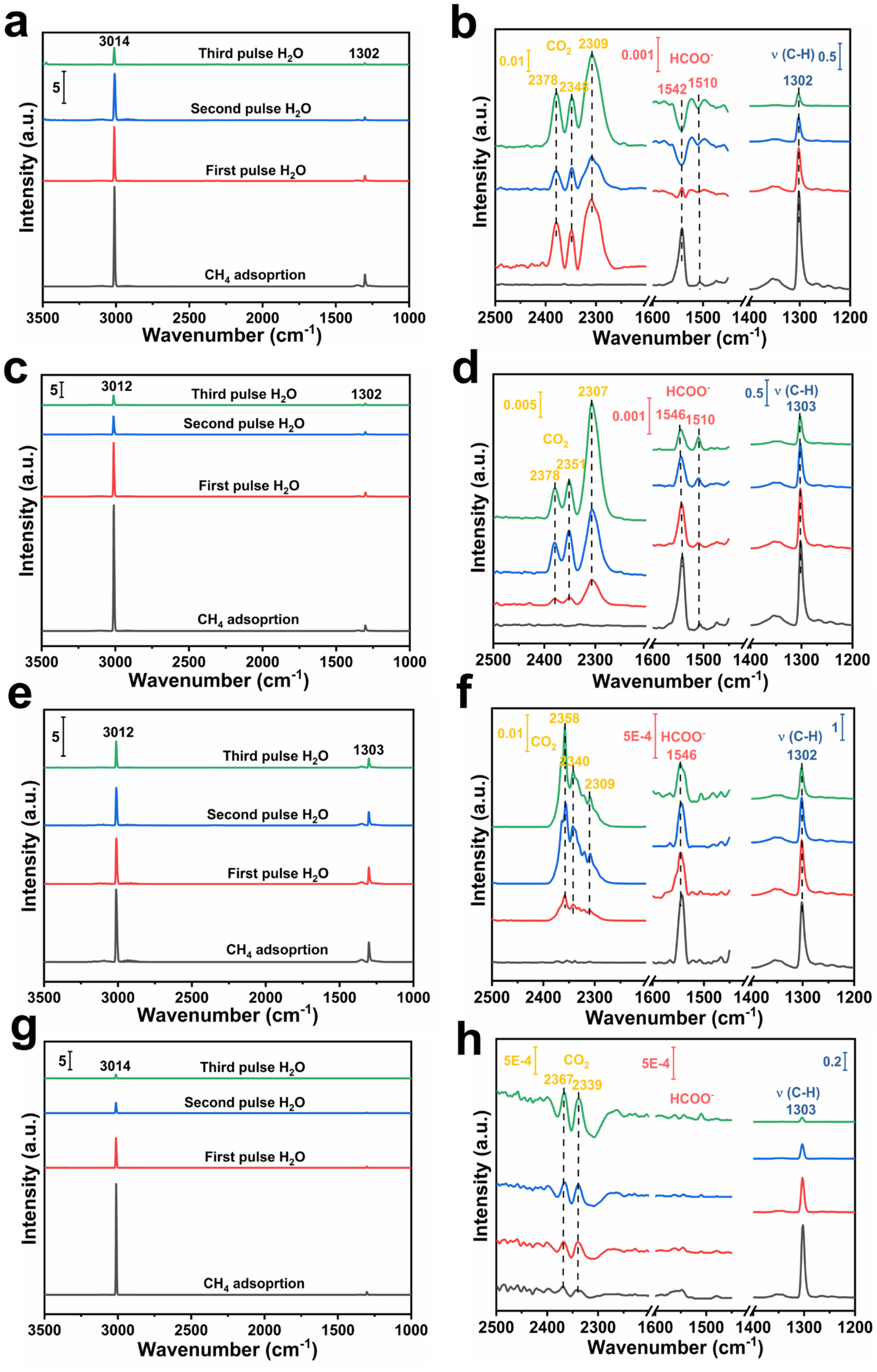

Supplementary Figure 46. ***In situ* DRIFT spectra of various catalysts.** *In situ* DRIFT spectra of CH_4 adsorption and subsequent H_2O pulse on **a**, **b** 0.5RhNi/TiO_2, **c**, **d** Ni/TiO_2, **e**, **f** Rh/Ni and **g**, **h** Rh/TiO_2 catalyst at 400 °C.

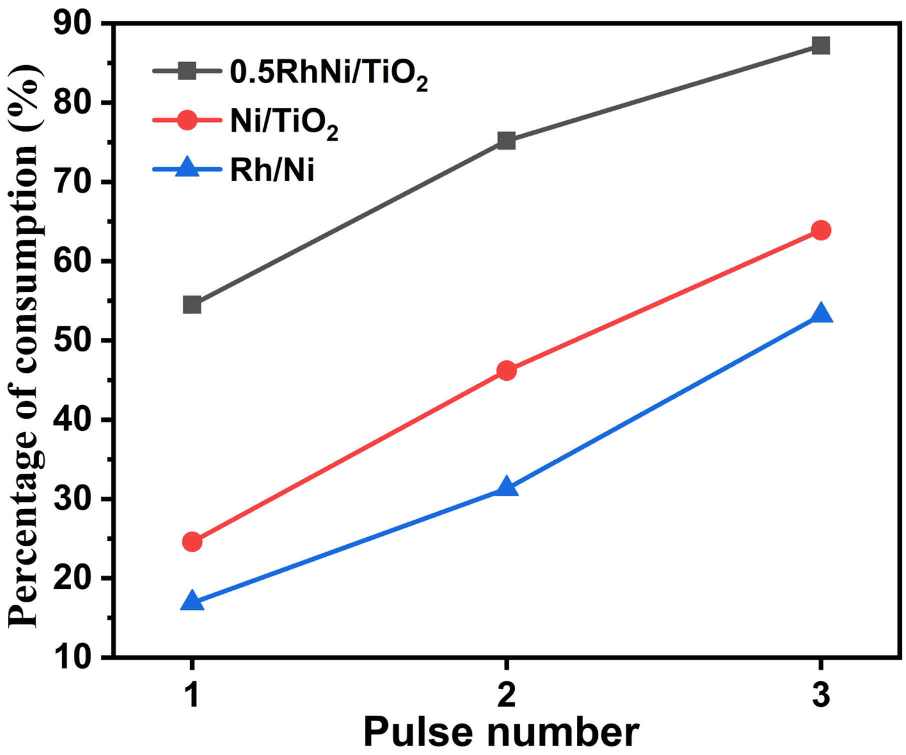

Supplementary Figure 47. Statistical result of *in situ* DRIFT spectra. Consumption percentage of CH_x species based on the descent rate of ν_{as}(CH) vibration peak at 1302 cm^{-1} from *in situ* DRIFT spectra (Supplementary Fig. 43).

Supplementary Note 18

In situ DRIFT spectroscopy was caried out to reveal the reaction mechanism of CH_x steam reforming process. As shown in Supplementary Figs. 46a and 46b, after the adsorption of CH_4, the bands at 3014, 1302, 1542 and 1510 cm^{-1} on the surface of 0.5RhNi/TiO_2 are attributed to the δ(CH), ν_{as}(CH) and formate vibration peaks, respectively[27]. After the subsequent H_2O pulse, a quick decline of formate vibration peaks is observed, accompanied with the appearance of CO_2 species at 2378-2309 cm^{-1}, indicating that formate is a crucial reaction intermediate during CH_4 reforming process. A similar measurement was carried out over Ni/TiO_2, Rh/Ni and Rh/TiO_2 catalysts, and the results were shown in Supplementary Figs. 46c-46h. Compared with the 0.5RhNi/TiO_2 catalyst, the formate vibration peaks display a moderate decrease in the cases of

Ni/TiO_2 and Rh/Ni catalysts. We further studied the reaction rate of CH_4 steam reforming according to the descent rate of ν_{as}(CH) vibration peak, and the results (Supplementary Fig. 47) demonstrated the highest catalytic activity of $0.5RhNi/TiO_2$ catalyst among the three samples. In addition, for the Rh/TiO_2 catalyst, no obvious intermediate was found, and only a weak CO_2 peak appeared after H_2O pulsing, owing to its poor reforming activity. A rapid decline of ν_{as}(CH) singal is related to the weak methane adsorption.

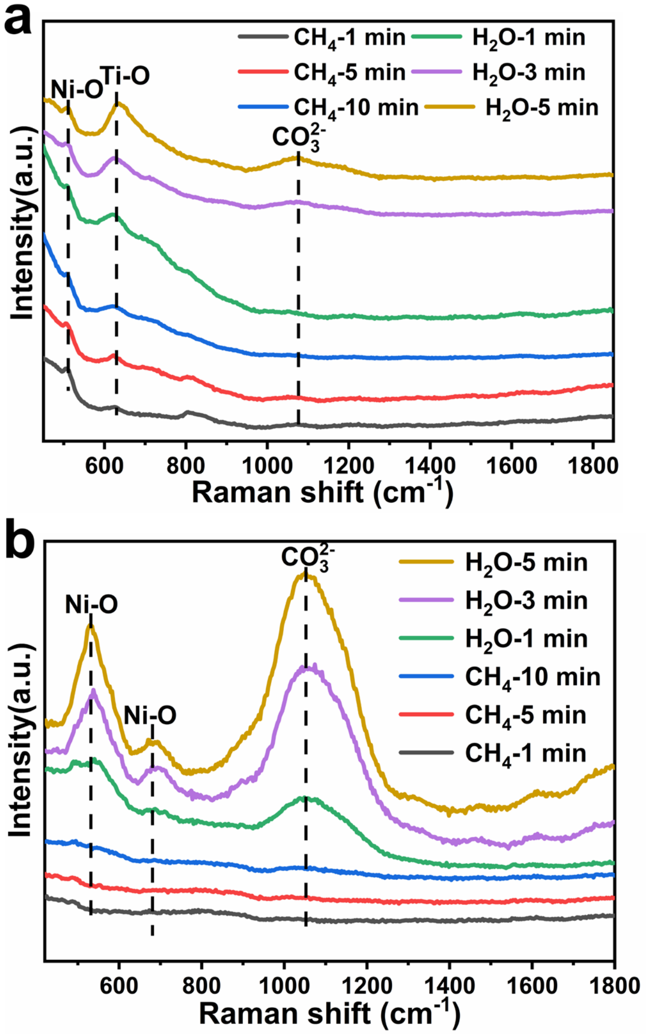

Supplementary Figure 48. *In situ* Raman spectra of 0.5RhNi/TiO_2 and Rh/Ni. *In situ* Raman spectra of CH_4 and H_2O sequential adsorption over **a** 0.5RhNi/TiO_2 and **b** Rh/Ni catalyst at 400 °C, respectively.

Supplementary Note 19

The *in situ* Raman spectroscopy was used to study CH_x steam reforming process over 0.5RhNi/TiO_2 and Rh/Ni catalysts (Supplementary Fig. 48). No obvious C species was observed after CH_4 adsorption at 400 °C on both catalysts, indicating that CH_4 does not undergo complete dehydrogenation. When switching to a saturated water vapor, CO_3^{2-} resulting from CO_2 showed a stronger binding to the Rh/Ni catalyst than the 0.5RhNi/TiO_2 one.

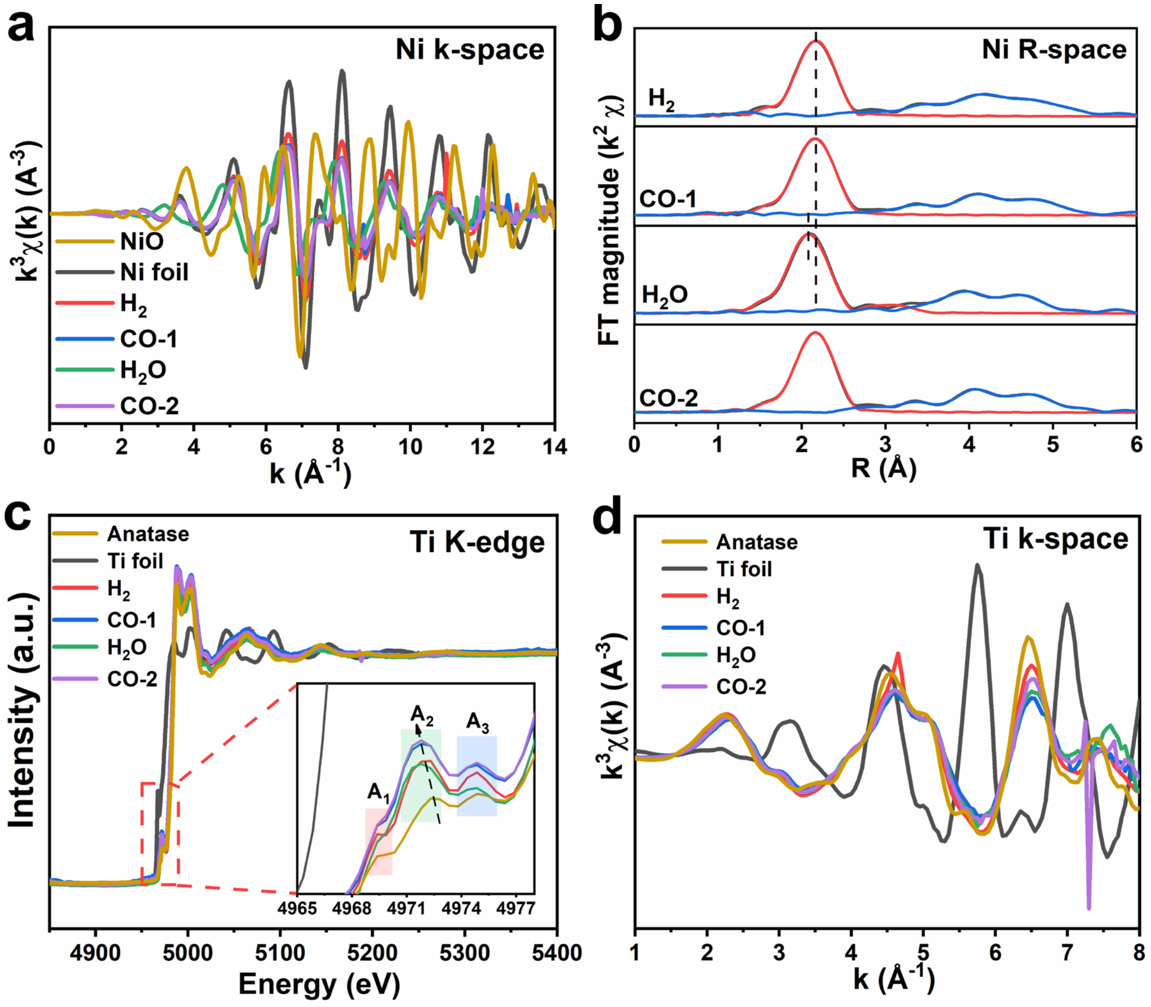

Supplementary Figure 49. *In situ* XAFS analysis of 0.5RhNi/TiO_2. **a** *In situ* Ni K-edge EXAFS spectra on k-space, **b** the fitting results of Ni K-edge Fourier-transform EXAFS spectra on R-space, **c** *in situ* normalized Ti K-edge XANES spectra and **d** normalized Ti K-edge EXAFS spectra in k space for CO and H_2O adsorption on 0.5RhNi/TiO_2 catalyst at 400 °C (H_2, CO-1, H_2O and CO-2 denote spectra after H_2 pretreatment, the first CO adsorption, H_2O adsorption and the second CO adsorption respectively for 20 min on 0.5RhNi/TiO_2 catalyst).

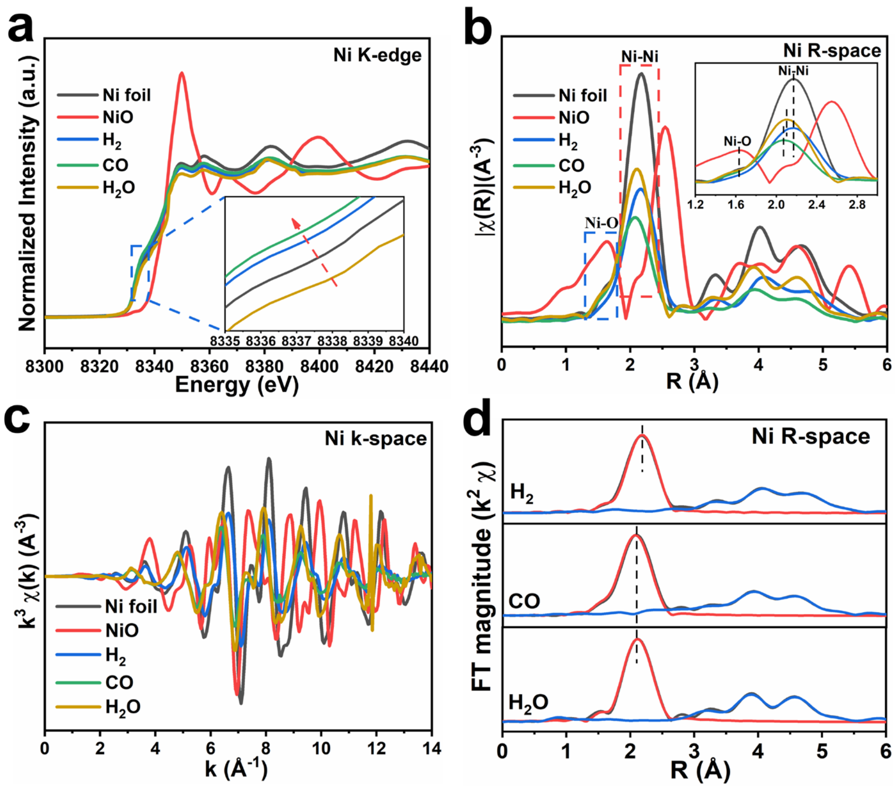

Supplementary Figure 50. *In situ* XAFS analysis of Rh/Ni. **a** *In situ* normalized Ni K-edge XANES spectra, **b** EXAFS spectra of Ni K-edge on R-space, **c** normalized Ni K-edge EXAFS spectra on k-space and **d** the fitting results of Ni K-edge Fourier-transform EXAFS spectra on R-space for CO and H_2O adsorption on Rh/Ni catalyst at 400 °C (H_2, CO and H_2O denote spectra after H_2 pretreatment, CO adsorption and H_2O adsorption respectively for 20 min on Rh/Ni catalyst).

Supplementary Note 20

In situ Ni K-edge XANES and EXAFS spectra were applied to reveal the dynamic change during the WGSR in the presence of Rh/Ni catalyst (Supplementary Fig. 50). As shown in Supplementary Fig. 50a, the absorption edge moves firstly to lower energy and then back to higher energy with the introduction of sequential CO and H_2O, respectively. The results correspond to the reduction and oxidation of Ni sites due to CO disproportionation and H_2O dissociation. The Ni–Ni bond length becomes shorter when exposing to CO (Supplementary Figs. 50b and 50d, and

Supplementary Table 7), indicating Ni species undergoes reconstitution resulting from a strong binding toward CO_2 or carbonate species. After switching to saturation water vapour, a subtle Ni–O bond derived from H_2O dissociation is found, indicating that the Ni species participates in water activation. This is consistent with the results of quasi-*in situ* XPS and *in situ* Raman (Supplementary Figs. 40c and 44g).

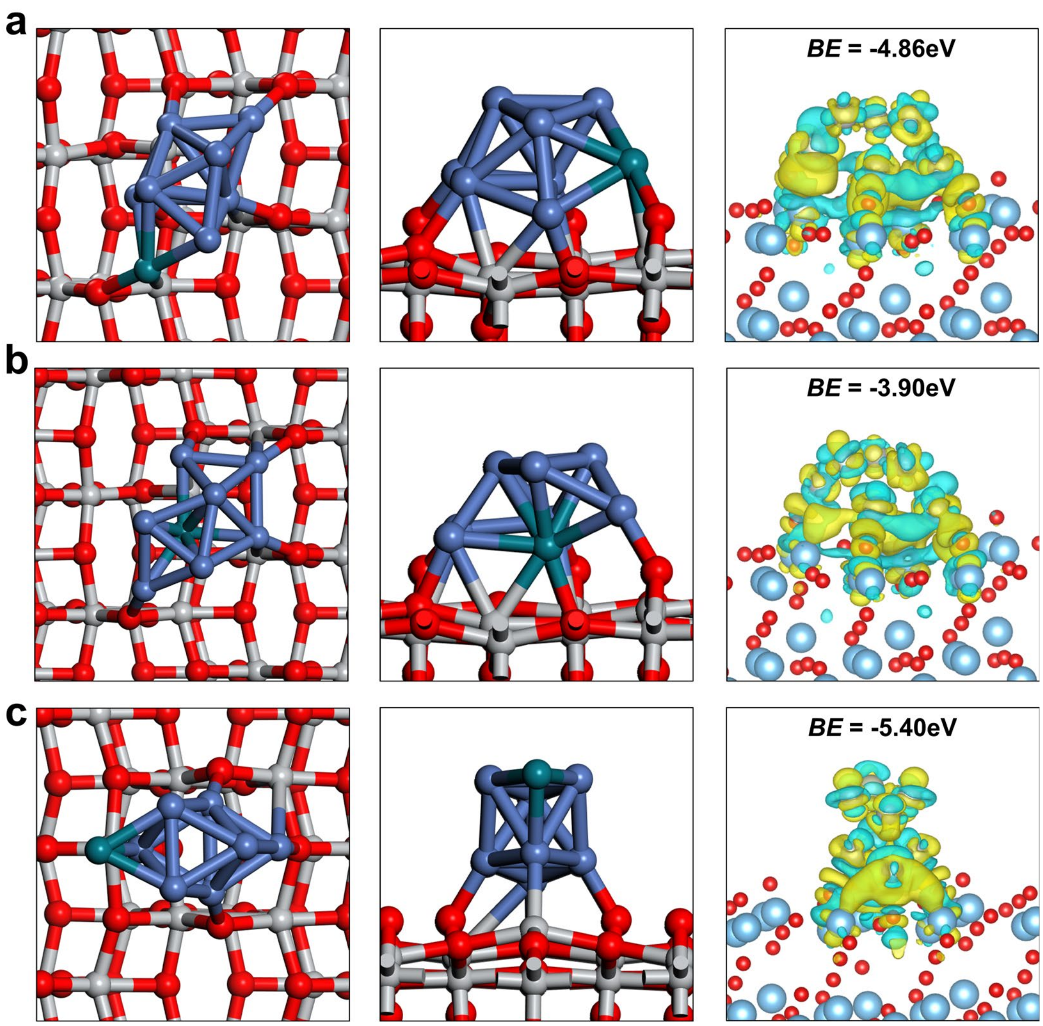

Supplementary Figure 51. DFT calculation models of Rh_1Ni_7-$TiO_{2–x}$. Structure model screening of Rh_1Ni_7-$TiO_{2–x}$: **a** Rh atom is located at the surface of Ni cluster; **b** Rh atom is located at the center of Ni cluster; **c** Rh atom is located at the top of Ni cluster (the left, middle and right figures show the top view, the front view, and the differential charge density analysis, respectively. *BE* is the binding energy).

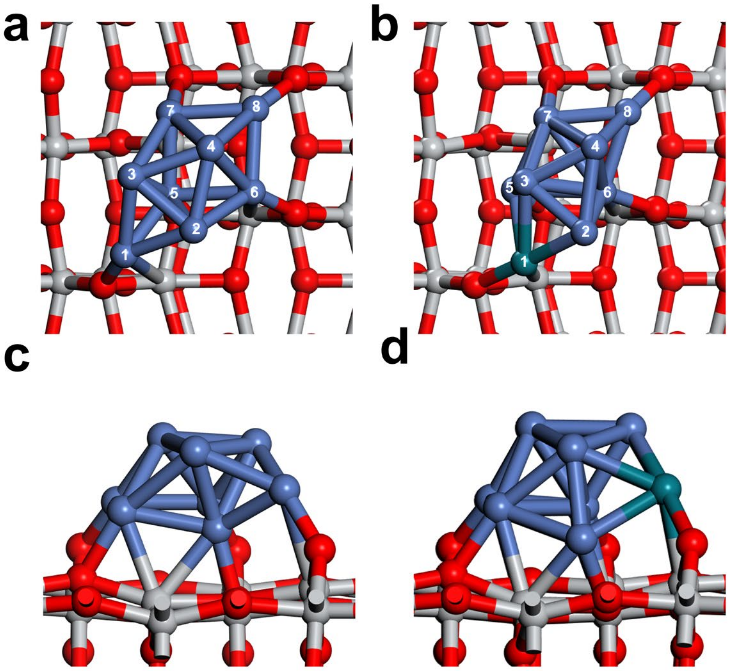

e

Atomic number	Ni_8/TiO_{2-x}	Rh_1Ni_7/TiO_{2-x}
1	-0.178	-0.214
2	+0.009	+0.019
3	-0.066	+0.023
4	+0.026	+0.038
5	-0.077	+0.002
6	-0.245	-0.336
7	-0.106	-0.170
8	-0.310	-0.205
Total Charge Density	-0.947	-0.843

Supplementary Figure 52. DFT calculation models and Bader charge analysis of Rh_1Ni_7-TiO_{2-x} and Ni_8/TiO_{2-x}. Catalysts model of **a**, **c** Ni_8/TiO_{2-x} and **b**, **d** Rh_1Ni_7/TiO_{2-x}. **e** Bader charge analysis.

Supplementary Note 21

According to the STEM results (Fig. 2 and Supplementary Fig. 19), three types of Rh_1Ni_7-TiO_{2-x} model with the interfacial structure between Rh_1Ni_7 bi-metal and TiO_2 support were built (Supplementary Fig. 51). Based on the differential charge density analysis, the binding energy (*BE*) declines in the following sequence: c > a > b. However, a large structural deformation exists in the structural model c. In particular, the oxygen vacancy at the interface is squeezed out, indicating a significant deformation of TiO_2 support. Thus, the model a is the most stable structure, which is used for the following calculations. The Bader charge analysis of metal atoms over Ni_8/TiO_{2-x} (-0.947 eV) and Rh_1Ni_7/TiO_{2-x} (-0.843 eV) catalysts models prove that the SBMSI weakens the charge transfer from the TiO_{2-x} support to the Rh_1Ni_7 bimetallic interface (Supplementary Fig. 52).

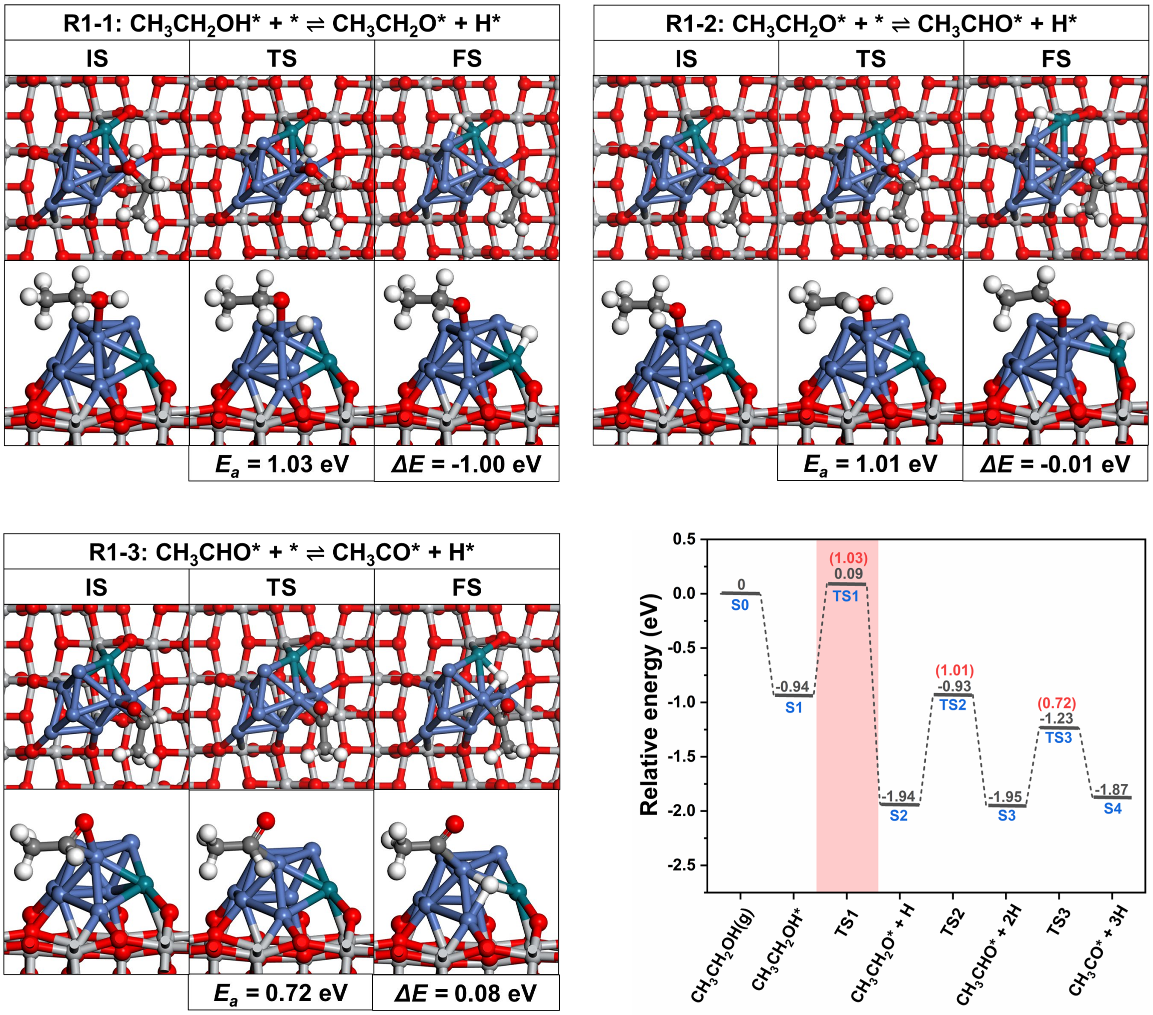

Supplementary Figure 53. DFT studies for CH_3CH_2OH dehydrogenation on Rh_1Ni_7/TiO_{2-x}. Calculated potential energy diagram and corresponding geometric structures for successive dehydrogenation of ethanol molecule *via* hydroxy, methylene and methine groups (Route 1) on Rh_1Ni_7/TiO_{2-x} (IS, TS and FS represent the initial state, transition state and final state, respectively; black and red numbers denote adsorption energy and reaction energy barrier, respectively).

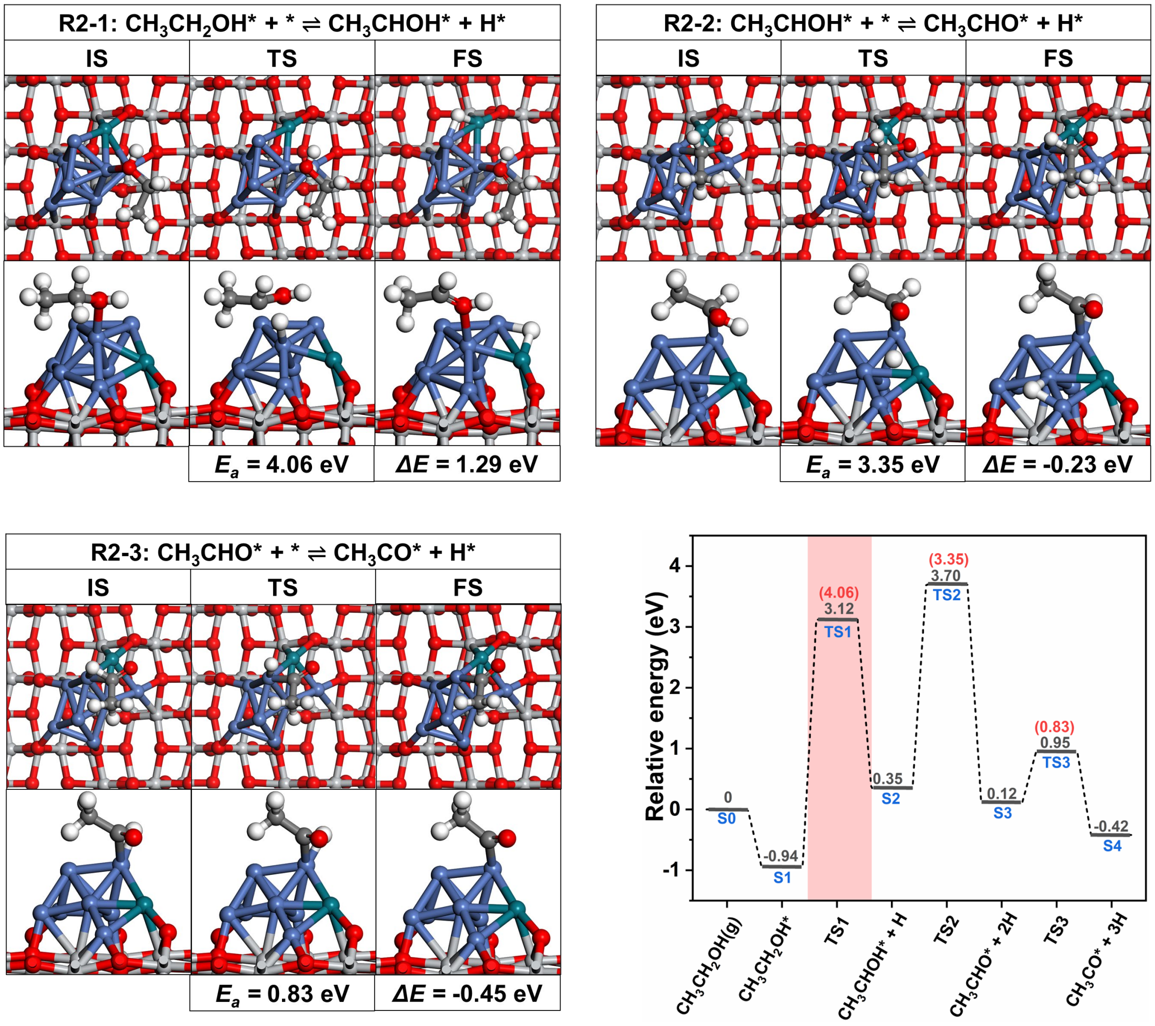

Supplementary Figure 54. DFT studies for CH_3CH_2OH dehydrogenation on Rh_1Ni_7/TiO_{2-x}. Calculated potential energy diagram and corresponding geometric structures for successive dehydrogenation of ethanol molecule *via* methylene, hydroxy and methine groups (Route 2) on Rh_1Ni_7/TiO_{2-x} (IS, TS and FS represent the initial state, transition state and final state, respectively; black and red numbers denote adsorption energy and reaction energy barrier, respectively).

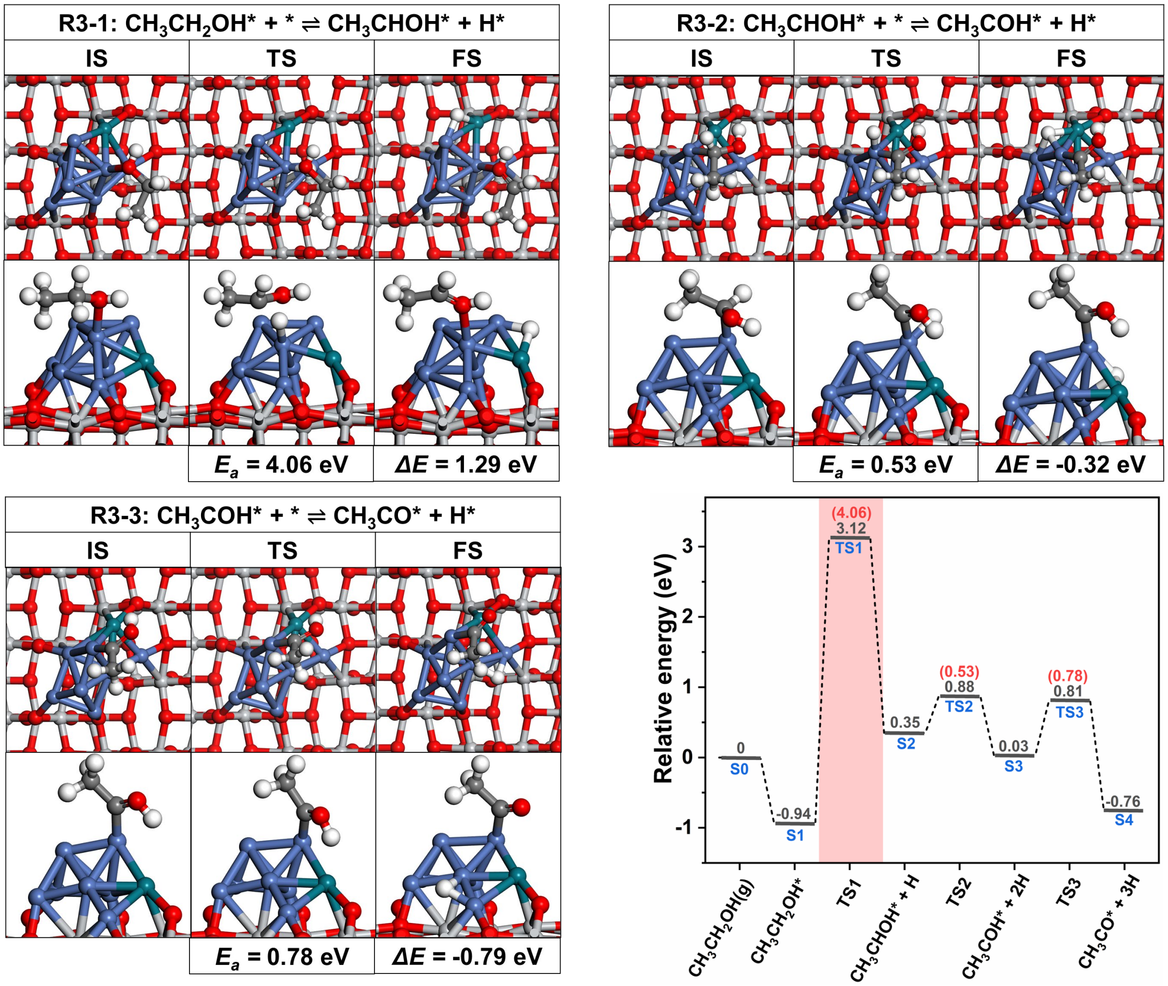

Supplementary Figure 55. DFT studies for CH_3CH_2OH dehydrogenation on Rh_1Ni_7/TiO_{2-x}. Calculated potential energy diagram and corresponding geometric structures for successive dehydrogenation of ethanol molecule *via* methylene, methine and hydroxy groups (Route 3) on Rh_1Ni_7/TiO_{2-x} (IS, TS and FS represent the initial state, transition state and final state, respectively; black and red numbers denote adsorption energy and reaction energy barrier, respectively).

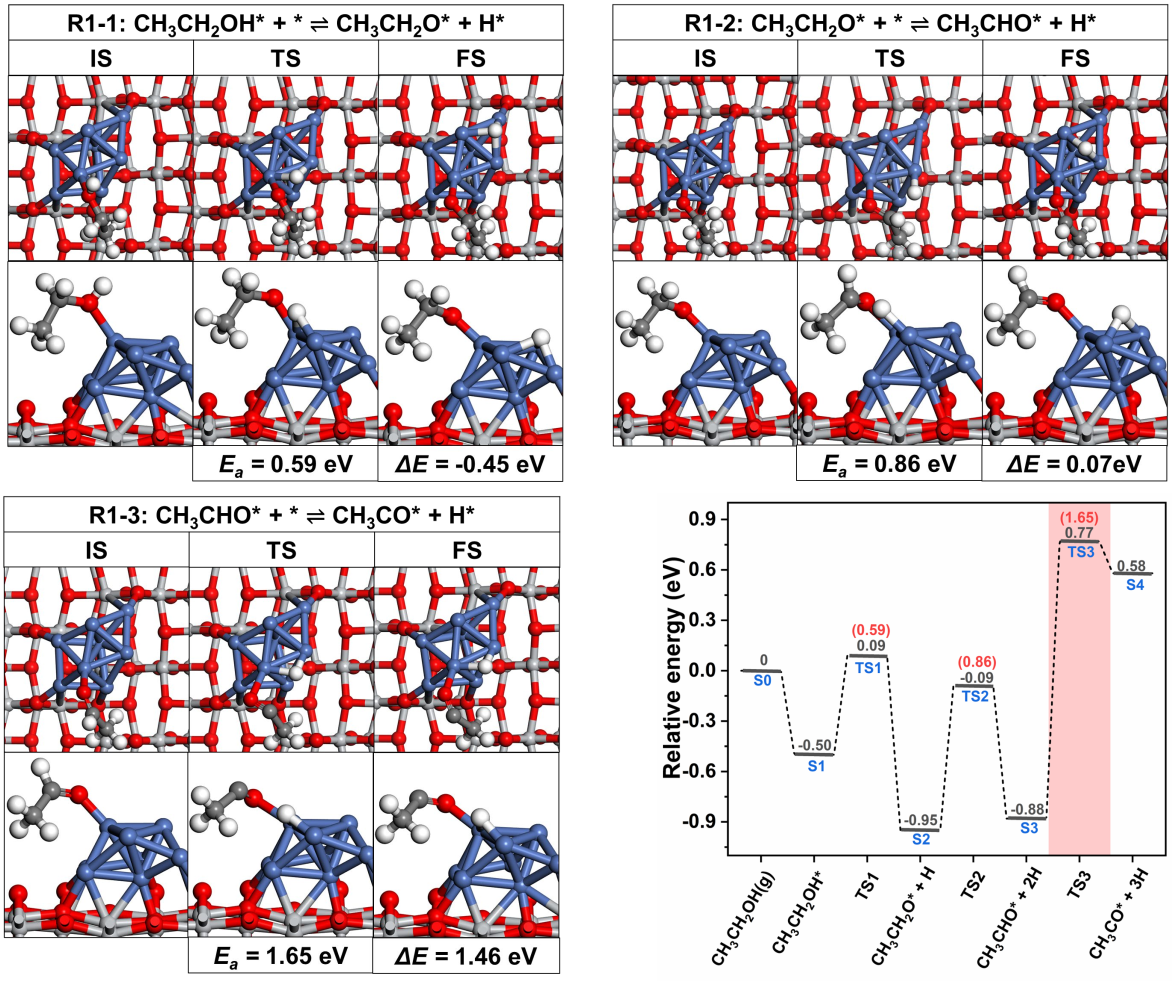

Supplementary Figure 56. DFT studies for CH_3CH_2OH dehydrogenation on Ni_8/TiO_{2-x}. Calculated potential energy diagram and corresponding geometric structures for successive dehydrogenation of ethanol molecule *via* hydroxy, methylene and methine groups (Route 1) on Ni_8/TiO_{2-x} (IS, TS and FS represent the initial state, transition state and final state, respectively; black and red numbers denote adsorption energy and reaction energy barrier, respectively).

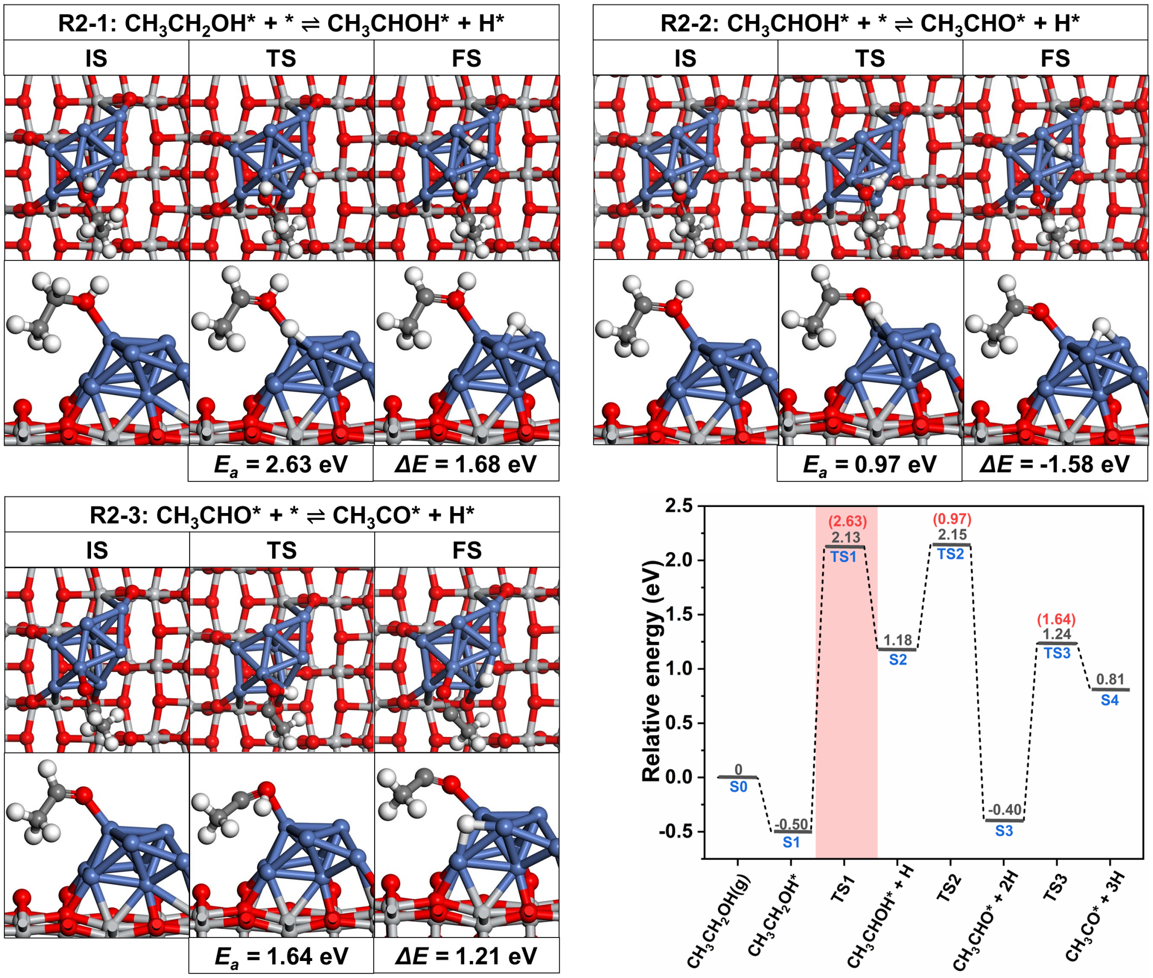

Supplementary Figure 57. DFT studies for CH_3CH_2OH dehydrogenation on Ni_8/TiO_{2-x}. Calculated potential energy diagram and corresponding geometric structures for successive dehydrogenation of ethanol molecule *via* methylene, hydroxy and methine groups (Route 2) on Ni_8/TiO_{2-x} (IS, TS and FS represent the initial state, transition state and final state, respectively; black and red numbers denote adsorption energy and reaction energy barrier, respectively).

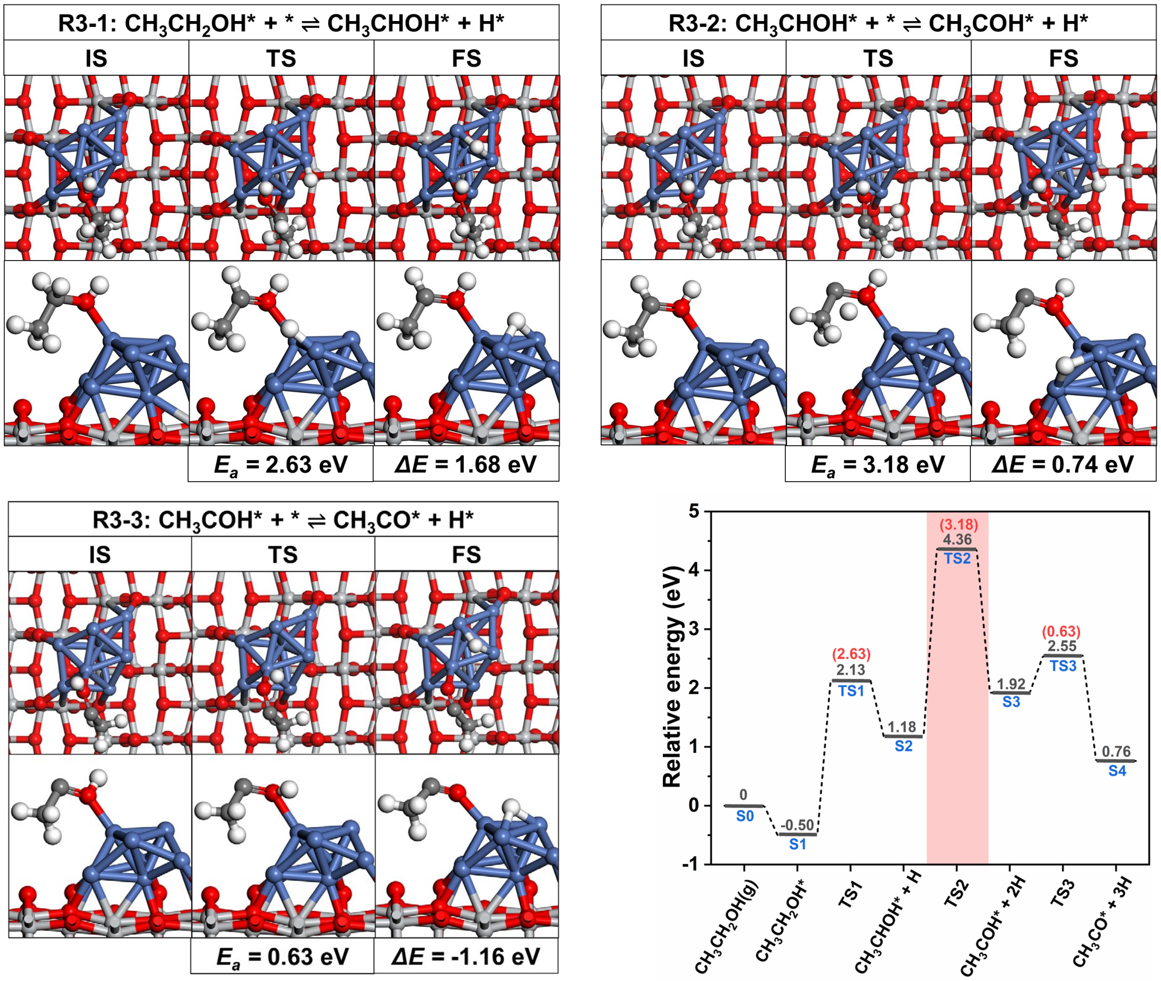

Supplementary Figure 58. DFT studies for CH_3CH_2OH dehydrogenation on Ni_8/TiO_{2-x}. Calculated potential energy diagram and corresponding geometric structures for successive dehydrogenation of ethanol molecule *via* methylene, methine and hydroxy groups (Route 3) on Ni_8/TiO_{2-x} (IS, TS and FS represent the initial state, transition state and final state, respectively; black and red numbers denote adsorption energy and reaction energy barrier, respectively).

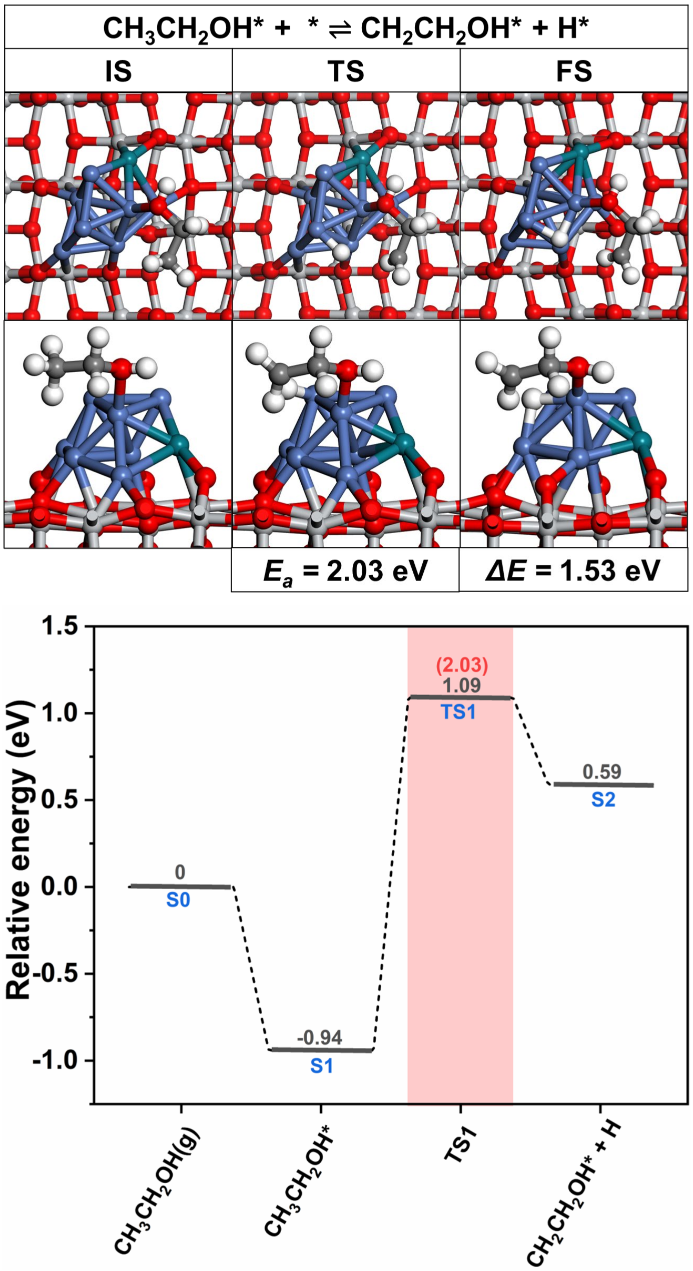

Supplementary Figure 59. DFT studies for methyl dehydrogenation in ethanol on Rh_1Ni_7/TiO_{2-x}. Calculated potential energy diagram and corresponding geometric structures for methyl dehydrogenation in ethanol on Rh_1Ni_7/TiO_{2-x} (IS, TS and FS represent the initial state, transition state and final state, respectively; black and red numbers denote adsorption energy and reaction energy barrier, respectively).

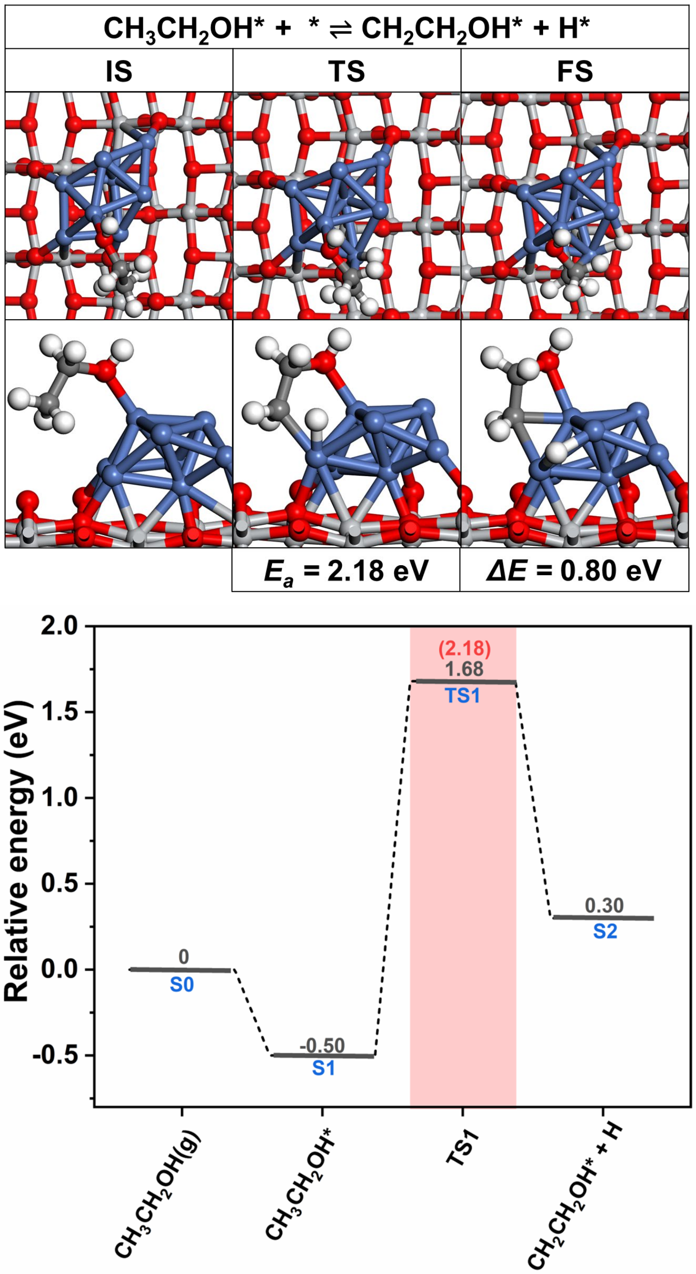

Supplementary Figure 60. DFT studies for methyl dehydrogenation in ethanol on Ni_8/TiO_{2-x}. Calculated potential energy diagram and corresponding geometric structures for methyl dehydrogenation in ethanol on Ni_8/TiO_{2-x} (IS, TS and FS represent the initial state, transition state and final state, respectively; black and red numbers denote adsorption energy and reaction energy barrier, respectively).

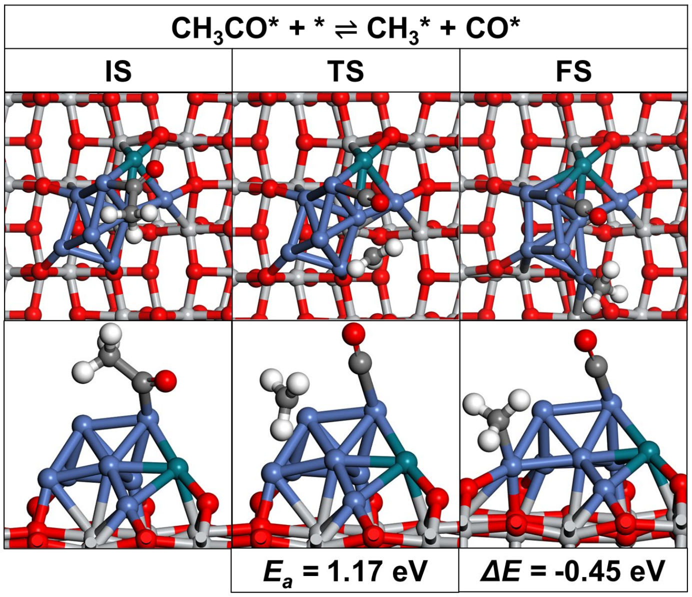

Supplementary Figure 61. DFT studies for C−C bond cleavage on Rh_1Ni_7/TiO_{2-x}. Calculated potential energy diagram and corresponding geometric structures for C−C bond cleavage in CH_3CO^* over Rh_1Ni_7/TiO_{2-x} (IS, TS and FS represent the initial state, transition state and final state, respectively; black and red numbers denote adsorption energy and reaction energy barrier, respectively).

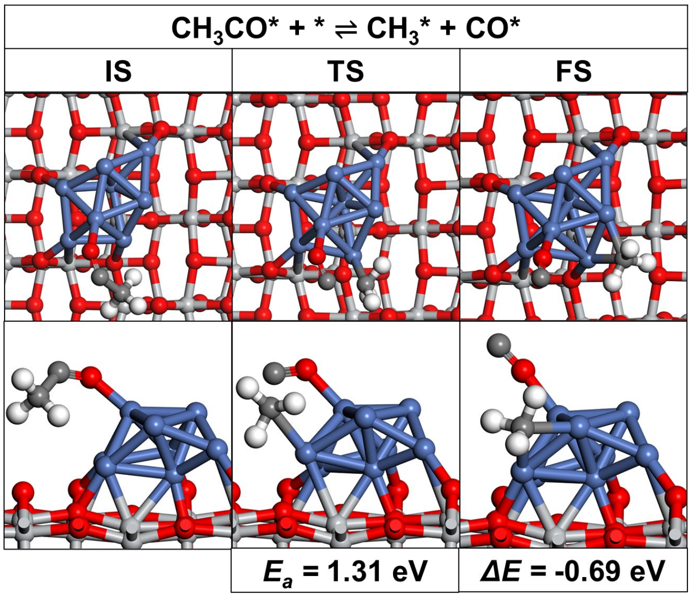

Supplementary Figure 62. DFT studies for C−C bond cleavage on Ni_8/TiO_{2-x}. Calculated potential energy diagram and corresponding geometric structures for C−C bond cleavage in CH_3CO^* over Ni_8/TiO_{2-x} (IS, TS and FS represent the initial state, transition state and final state, respectively; black and red numbers denote adsorption energy and reaction energy barrier, respectively).

Supplementary Note 22

As shown in Supplementary Figs. 53−58 and Supplementary Table 8, various ethanol dehydrogenation routes were calculated on Rh_1Ni_7/TiO_{2-x} and Ni_8/TiO_{2-x} samples, respectively, and the surface metal sites act as the active center in favor of ethanol activation adsorption. For Route 1 ($CH_3CH_2OH \rightarrow CH_3CH_2O^* \rightarrow CH_3CHO^* \rightarrow CH_3CO^*$), Route 2 ($CH_3CH_2OH \rightarrow CH_3CHOH^* \rightarrow CH_3CHO^* \rightarrow CH_3CO^*$) and Route 3 ($CH_3CH_2OH \rightarrow CH_3CHOH^* \rightarrow CH_3COH^* \rightarrow CH_3CO^*$), the reaction energy barriers over Rh_1Ni_7/TiO_{2-x} and Ni_8/TiO_{2-x} are 1.03, 4.06, 4.06 eV and 1.65, 2.63, 3.18 eV, respectively. For the methyl dehydrogenation in ethanol ($CH_3CH_2OH \rightarrow CH_2CH_2OH$), the reaction energy barriers are 2.03 and 2.18 eV over Rh_1Ni_7/TiO_{2-x} and Ni_8/TiO_{2-x} catalysts, respectively (Supplementary Figs. 59 and 60). Remarkably, the lowest reaction energy barrier for Route 1 indicates that the successive dehydrogenation of ethanol molecule (*via* hydroxy, methylene and methine groups) is the optimal path on both catalysts, which is consistent with the experimental results. Subsequently, the CH_3CO species undergoes C−C bond cleavage to generate CO and CH_3 with energy barriers of 1.17 and 1.31 eV on Rh_1Ni_7/TiO_{2-x} and Ni_8/TiO_{2-x} catalysts, respectively (Supplementary Figs. 61 and 62). Compared with Ni_8/TiO_{2-x}, the largely decreased reaction energy barriers for ethanol dehydrogenation and acetaldehyde decomposition on Rh_1Ni_7/TiO_{2-x} demonstrate the advantages of bimetallic synergistic effect towards ethanol conversion.

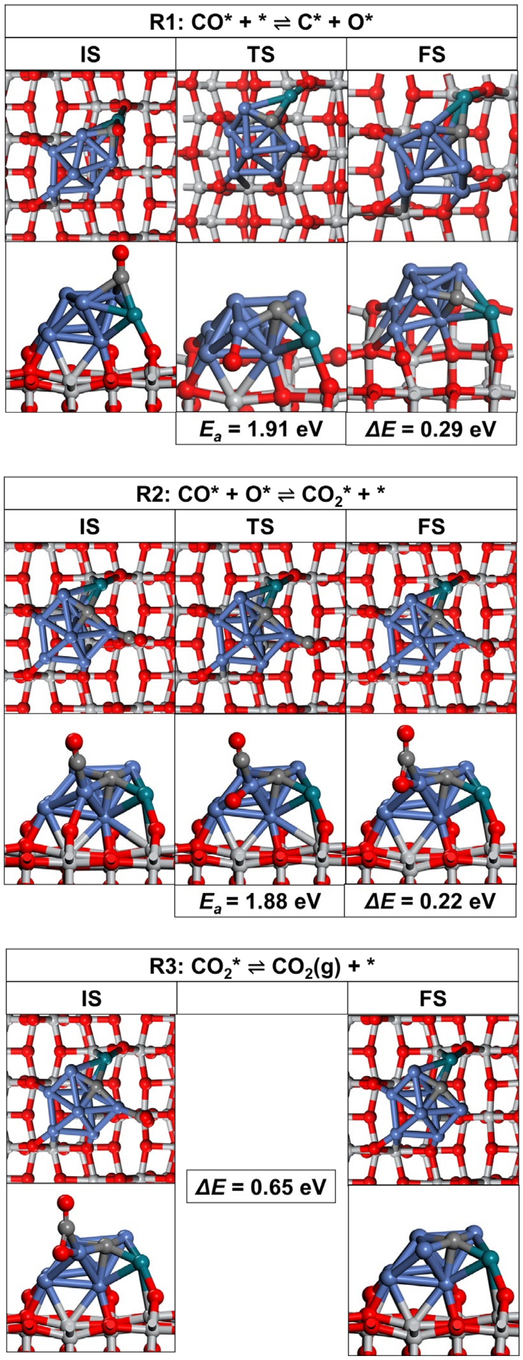

Supplementary Figure 63. DFT studies for CO direct dissociation on Rh_1Ni_7/TiO_{2-x}. Calculated potential energy diagram and corresponding. geometric structures for CO direct dissociation pathway over Rh_1Ni_7/TiO_{2-x} model (IS, TS and FS represent the initial state, transition state and final state, respectively).

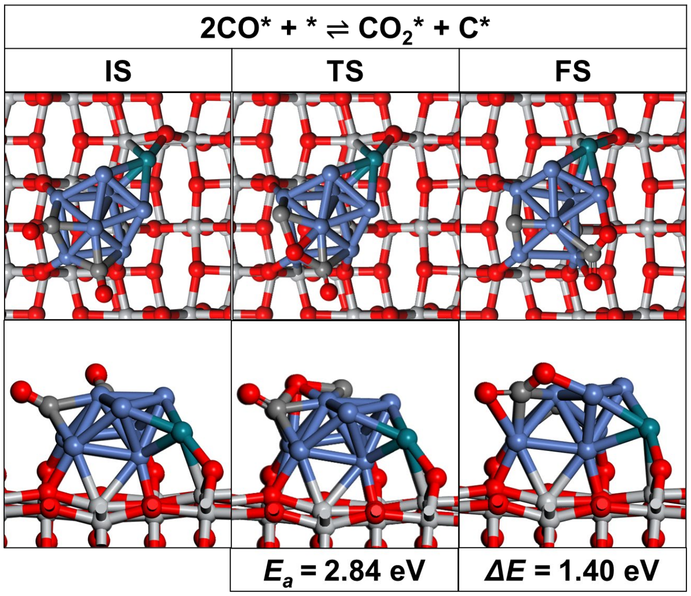

Supplementary Figure 64. DFT studies for CO association dissociation on Rh_1Ni_7/TiO_{2-x}. Calculated potential energy diagram and corresponding geometric structures for double CO association dissociation pathway over Rh_1Ni_7/TiO_{2-x} model (IS, TS and FS represent the initial state, transition state and final state, respectively).

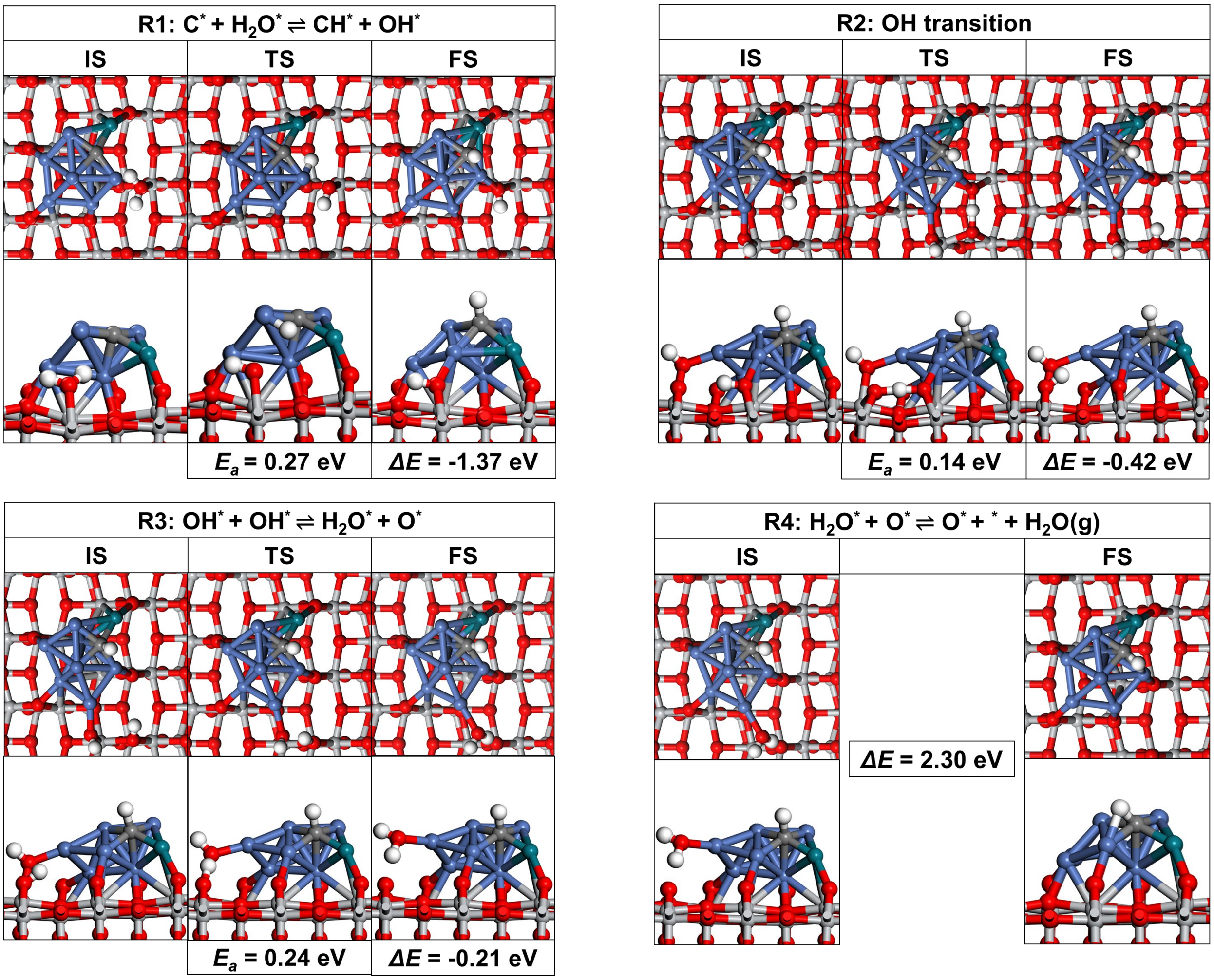

Supplementary Figure 65. DFT studies for H_2O dissociation on Rh_1Ni_7/TiO_{2-x}. Calculated potential energy diagram and corresponding geometric structures for H_2O dissociation to active oxygen followed by hydroxyl self-disproportionation pathway over Rh_1Ni_7/TiO_{2-x} model (IS, TS and FS represent the initial state, transition state and final state, respectively).

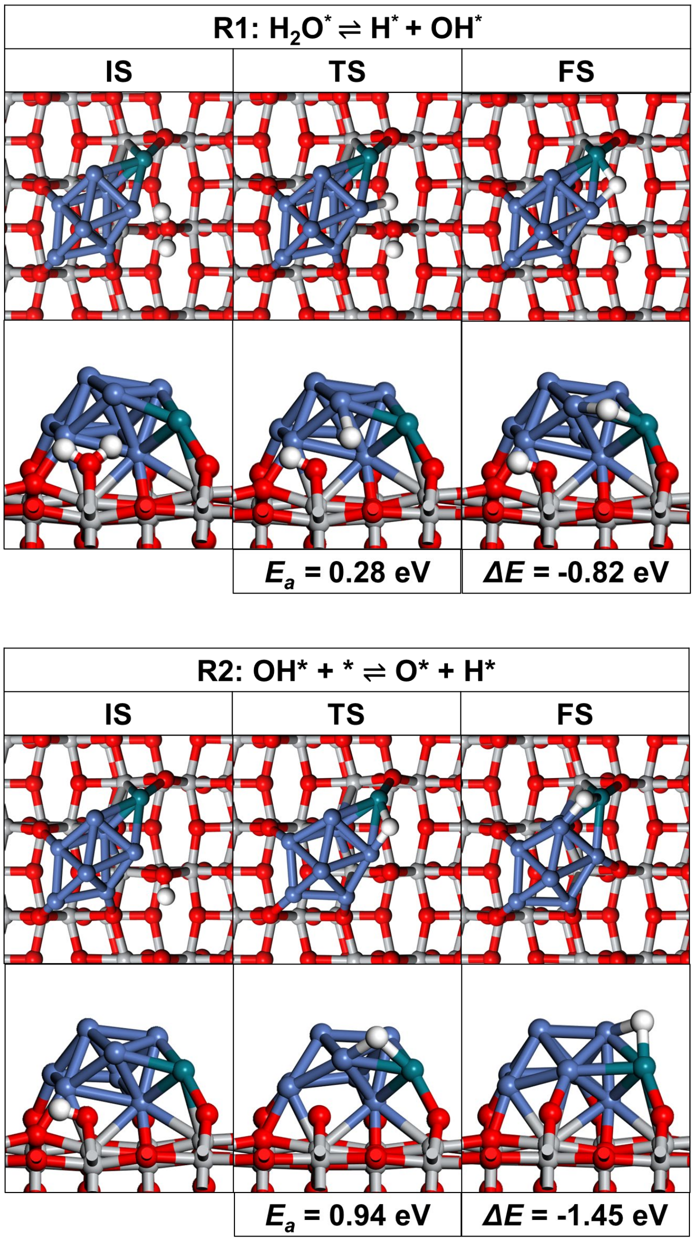

Supplementary Figure 66. DFT studies for H_2O continuous dissociation on Rh_1Ni_7/TiO_{2-x}. Calculated potential energy diagram and corresponding geometric structures for H_2O continuous dissociation pathway over Rh_1Ni_7/TiO_{2-x} model (IS, TS and FS represent the initial state, transition state and final state, respectively).

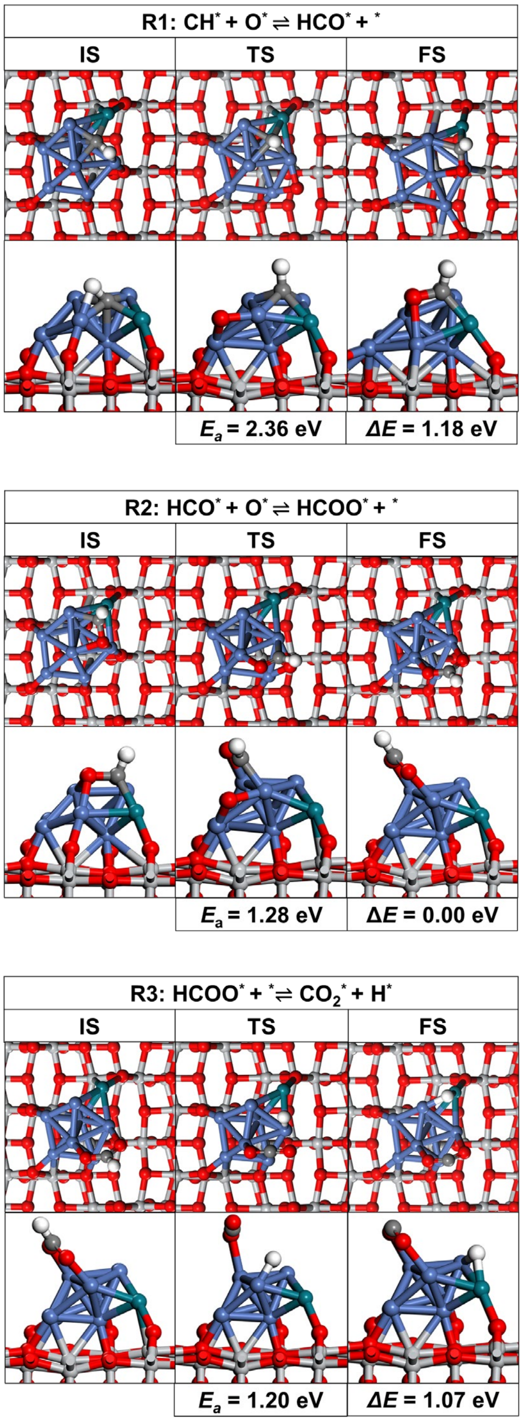

Supplementary Figure 67. DFT studies for formate generation and decomposition on Rh_1Ni_7/TiO_{2-x}. Calculated potential energy diagram and corresponding geometric structures for the generation of formate intermediate from HCO and active oxygen species, followed by a further decomposition over Rh_1Ni_7/TiO_{2-x} model (IS, TS and FS represent the initial state, transition state and final state, respectively).

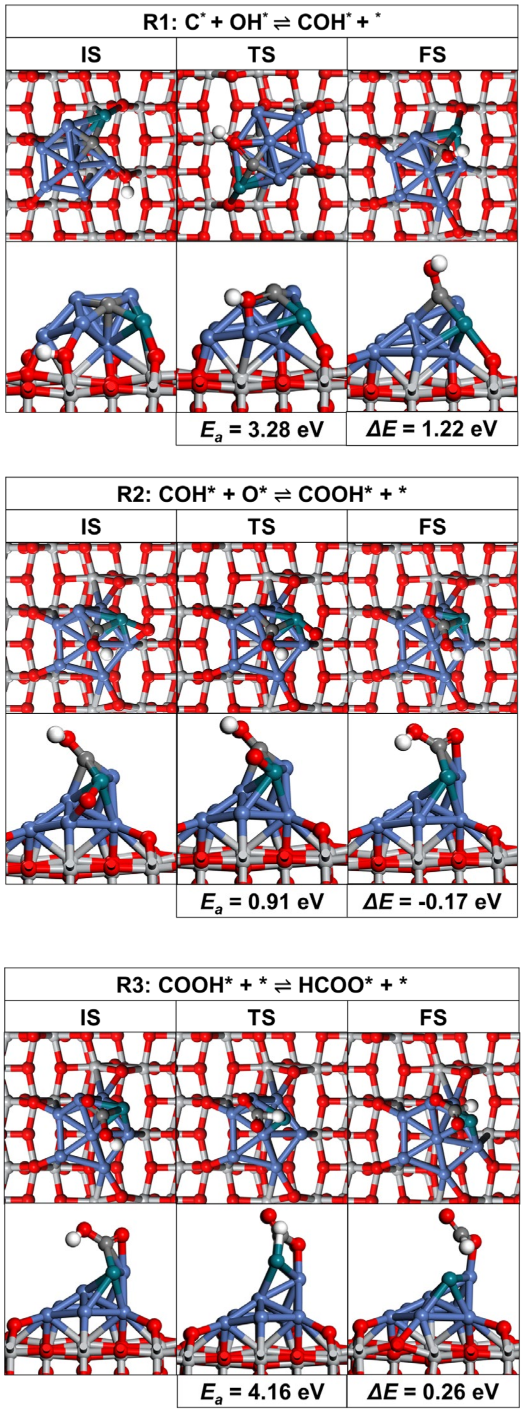

Supplementary Figure 68. DFT studies for formate generation on Rh_1Ni_7/TiO_{2-x}. Calculated potential energy diagram and corresponding geometric structures for the generation of carboxylate from COH and active oxygen species, followed by a subsequent transition to formate intermediate over Rh_1Ni_7/TiO_{2-x} model (IS, TS and FS represent the initial state, transition state and final state, respectively).

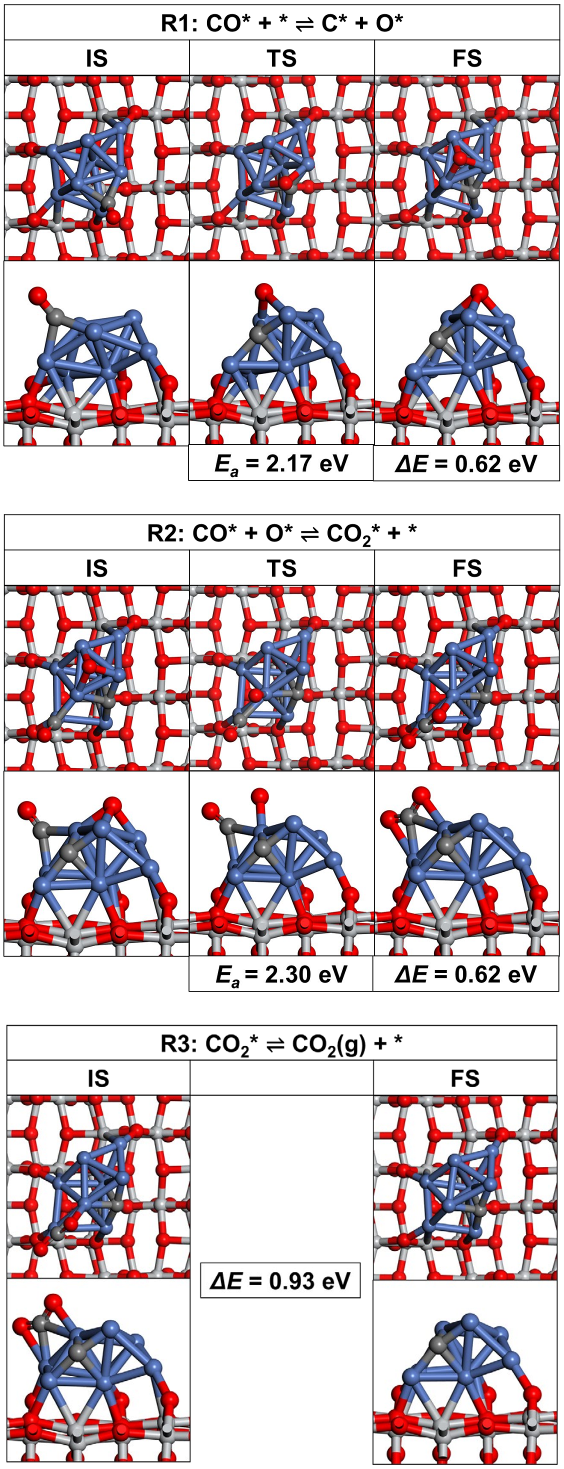

Supplementary Figure 69. DFT studies for CO direct dissociation on Ni_8/TiO_{2-x}. Calculated potential energy diagram and corresponding geometric structures for CO disproportionation reaction on Ni_8/TiO_{2-x} (IS, TS and FS represent the initial state, transition state and final state, respectively).

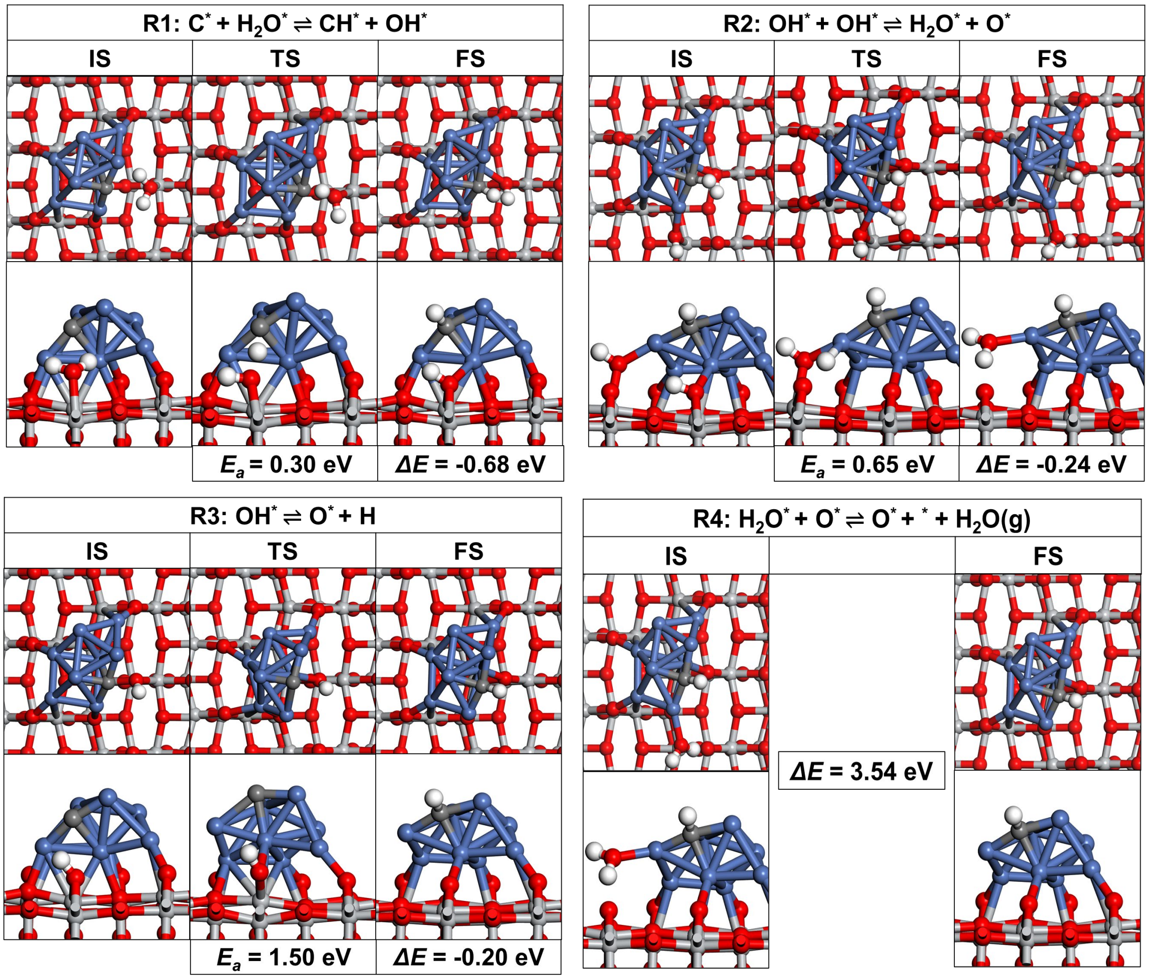

Supplementary Figure 70. DFT studies for H_2O dissociation on Ni_8/TiO_{2-x}. Calculated potential energy diagram and corresponding geometric structures for H_2O dissociation to active oxygen on Ni_8/TiO_{2-x} (IS, TS and FS represent the initial state, transition state and final state, respectively).

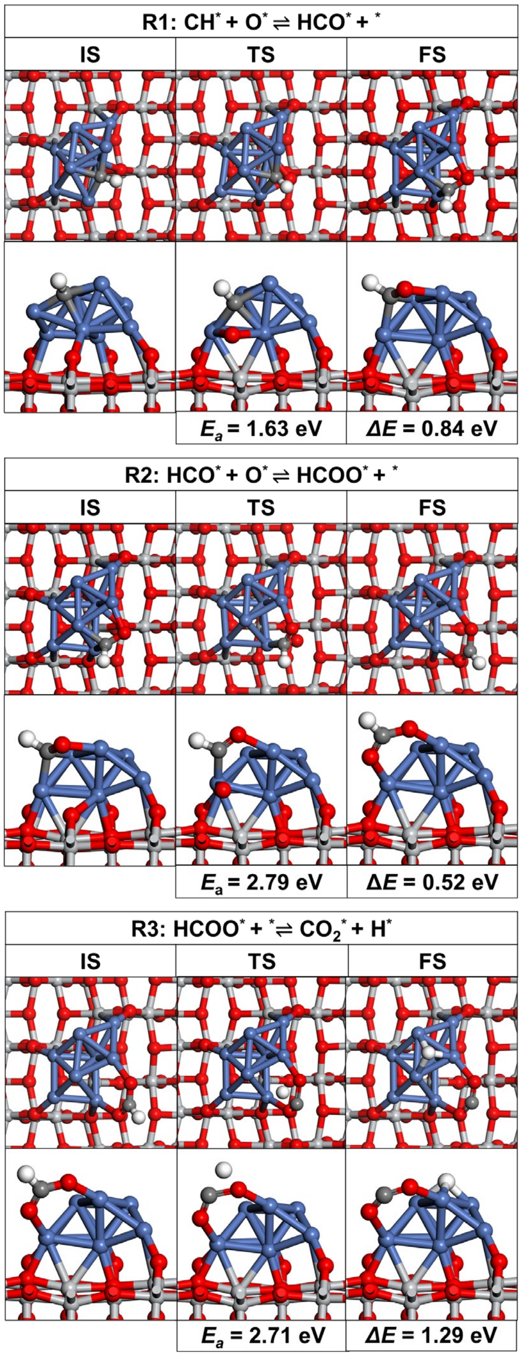

Supplementary Figure 71. DFT studies for formate generation and decomposition on Ni_8/TiO_{2-x}. Calculated potential energy diagram and corresponding geometric structures for the generation of formate intermediate from HCO and active oxygen species, followed by a further decomposition over Ni_8/TiO_{2-x} model (IS, TS and FS represent the initial state, transition state and final state, respectively).

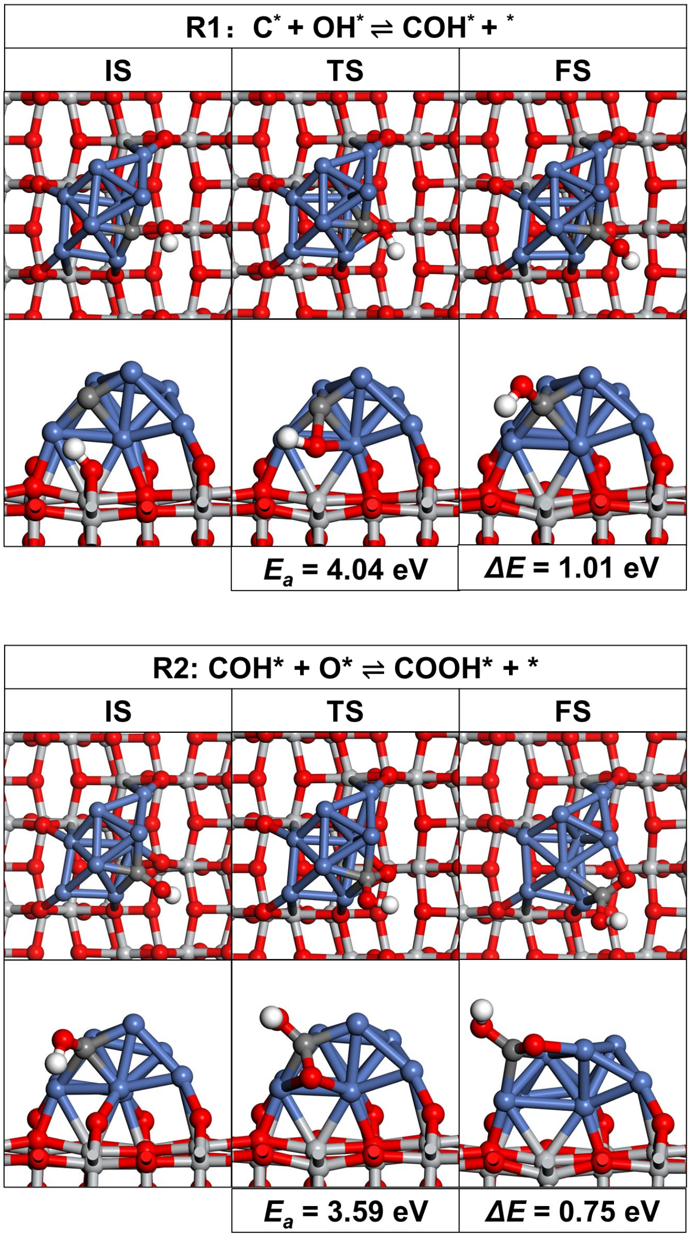

Supplementary Figure 72. DFT studies for the generation of carboxylate on Ni_8/TiO_{2-x}. Calculated potential energy diagram and corresponding geometric structures for the generation of carboxylate from COH* and active oxygen species, followed by a subsequent transition to formate intermediate on Ni_8/TiO_{2-x} (IS, TS and FS represent the initial state, transition state and final state, respectively).

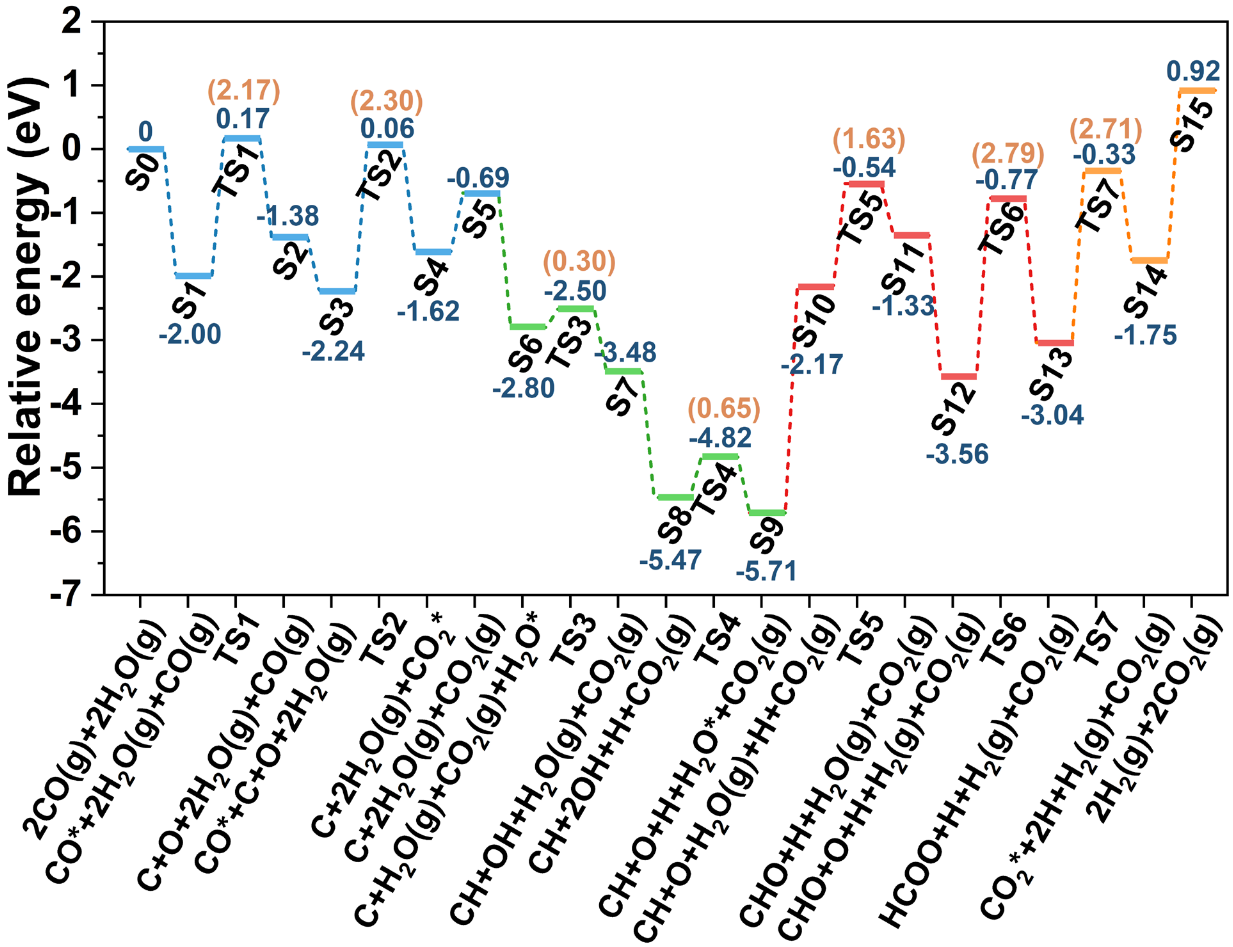

Supplementary Figure 73. Energy curve from DFT calculation on Ni_8/TiO_{2-x}. Reaction mechanism of stream reforming of CO on the surface of Ni_8/TiO_{2-x} (S, TS and ISS represent a stable sorption state, a transition state and an intermediate stable state, respectively). Blue, green, red and orange dotted lines denote CO disproportionation, H_2O dissociation, formate generation and CO_2 desorption, respectively; blue and orange numbers represent adsorption energy and reaction energy barrier, respectively).

Supplementary Note 23

For the CO disproportionation process, two reaction pathways have been reported including CO direct dissociation pathway (CO → C + O; CO + O → CO_2) and double CO association dissociation pathway (2CO → C + CO_2). In the first case (Supplementary Fig. 63: R1), one CO molecule undergoes adsorption at the hollow site near two Ni atoms and one Rh atom on Rh_1Ni_7/TiO_{2-x} with a dissociation energy barrier of 1.91 eV; and the resulting active oxygen is embedded in the oxygen vacancy of TiO_{2-x}. Then, another one CO molecule reacts with interfacial O species to generate CO_2 with an energy barrier of 1.88 eV (Supplementary Fig. 63: R2), followed by CO_2 desorption from the catalyst surface to complete CO disproportionation reaction (Supplementary Fig. 63: R3). In contrast, for the double CO association dissociation, two CO molecules experience activation adsorption at adjacent Ni sites to form CO-CO structure, followed by dissociation to produce CO_2 and C species with a relatively high energy barrier of 2.84 eV (Supplementary Fig. 64). Therefore, CO direct dissociation is the thermodynamically favorable pathway over Rh_1Ni_7/TiO_{2-x} with energy barrier of 1.91 eV. In addition, the similar CO disproportionation path was also calculated on Ni_8/TiO_{2-x} (Supplementary Fig. 69), but displays a higher reaction energy barrier (2.30 eV).

The water dissociation at the interface oxygen vacancy over Rh_1Ni_7/TiO_{2-x} was further calculated. Two dissociation paths have been reported including hydroxyl self-disproportionation and water continuous dissociation path. In the former case (Supplementary Fig. 65), H_2O molecule firstly undergoes adsorption dissociation at the interface oxygen vacancy to generate hydrogen atom and hydroxyl species (H_2O → H + OH). Regardless of the presence of C species on the catalyst surface (Supplementary Fig. 65: R1 and Supplementary Fig. 66: R1), the water dissociation displays similar energy barriers (0.27 and 0.28 eV), indicating that the surface C species has no effect on water activation dissociation due to the different adsorption sites for C and

H_2O. Afterwards, two hydroxyls experience disproportionation to produce reactive oxygen species ($2OH \rightarrow H_2O + O$) with an energy barrier of 0.24 eV (Supplementary Fig. 65: R3). This process shows a much lower energy barrier compared with the hydroxyl continuous dissociation path ($OH \rightarrow O + H$) with an energy barrier of 0.94 eV (Supplementary Fig. 66: R2). Thus, the former is the dominant pathway for the generation of reactive oxygen species over Rh_1Ni_7/TiO_{2-x}. In addition, we also compared the two reaction paths for the generation of reactive oxygen species over Ni_8/TiO_{2-x} (Supplementary Fig. 70), and hydroxyl self-disproportionation pathway (Supplementary Fig. 70: R2) (0.65 eV) still possesses a lower energy barrier than hydroxyl continuous dissociation (Supplementary Fig. 70: R3) (1.50 eV).

Subsequently, the generation of formate intermediate from reaction between CH or C species and active oxygen was further calculated over Rh_1Ni_7/TiO_{2-x}. In the first path (shown in Supplementary Fig. 67), the active oxygen species derived from hydroxyl self-disproportionation migrates to the Ni site and combines with CH to produce HCO intermediate with an energy barrier of 2.36 eV (Supplementary Fig. 67: R1). Afterwards, the generated HCO continues to react with active oxygen species to produce formate intermediate with an energy barrier of 1.28 eV (Supplementary Fig. 67: R2). Finally, the generated formate intermediate undergoes dissociation to produce CO_2 and H species with an energy barrier of 1.20 eV (Supplementary Fig. 67: R3). Another path for the generation of formate intermediate based on C species reacts with hydroxy or active oxygen species was also calculated (Supplementary Fig. 68), and a relatively higher energy barrier (4.16 eV) ruled out this reaction path. In addition, we also compared the reaction paths of HCO and COH for the generation of formate intermediate over Ni_8/TiO_{2-x} (Supplementary Figs. 71 and 72), and the former still is a favorable reaction pathway. In contrast, the Rh_1Ni_7/TiO_{2-x} (2.36 eV) with SBMSI exhibits the lower reaction energy barrier for formate formation than

Ni_8/TiO_{2-x} (2.77 eV). The potential energy profiles over Ni_8/TiO_{2-x} catalyst was shown in the Supplementary Fig. 73 and Supplementary Table 80.

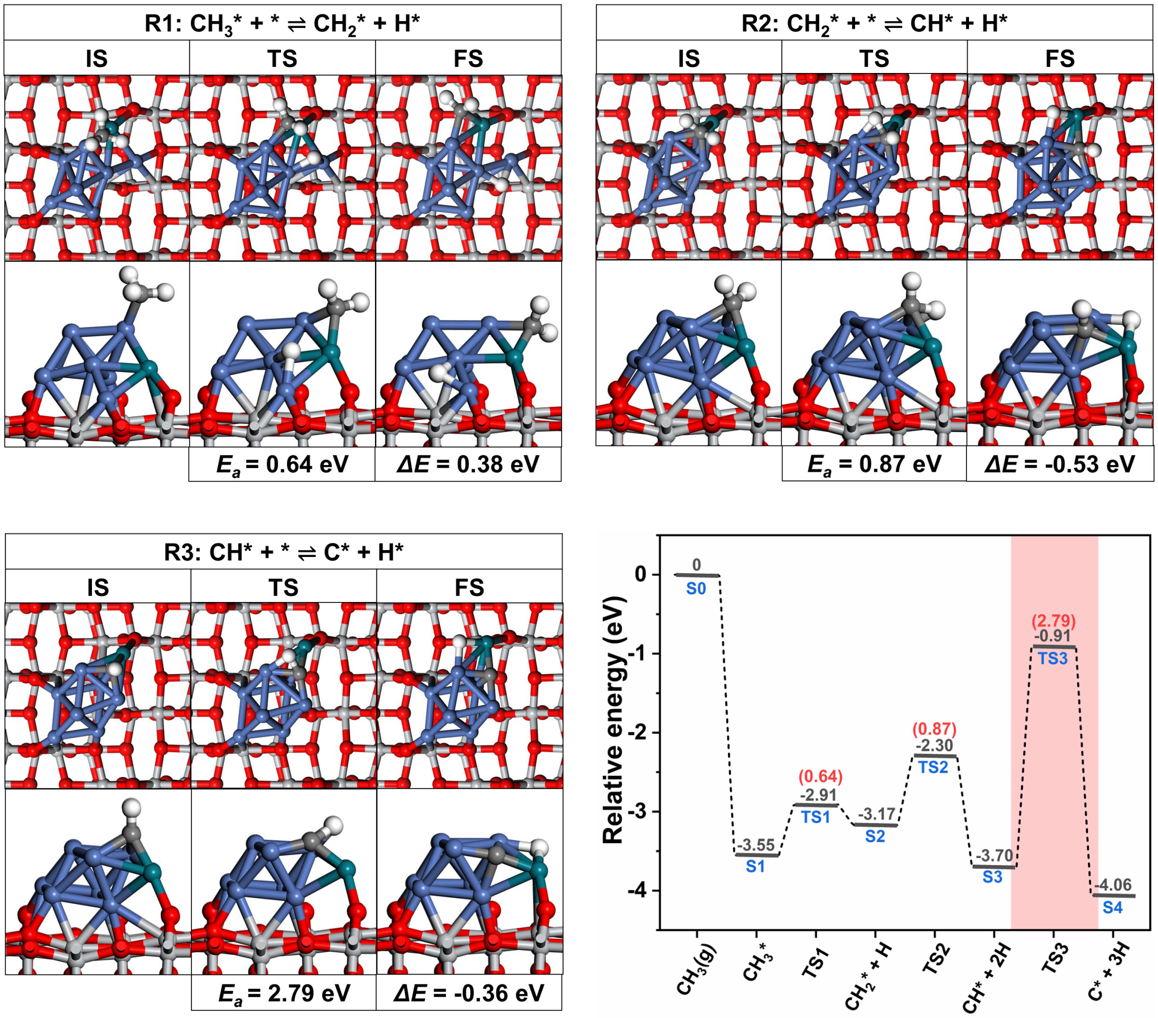

Supplementary Figure 74. DFT studies for methyl dehydrogenation on Rh_1Ni_7/TiO_{2-x}. Calculated potential energy diagram and corresponding geometric structures for methyl successive dehydrogenation processes over Rh_1Ni_7/TiO_{2-x} model (IS, TS and FS represent the initial state, transition state and final state, respectively; black and red numbers represent adsorption energy and reaction energy barrier, respectively).

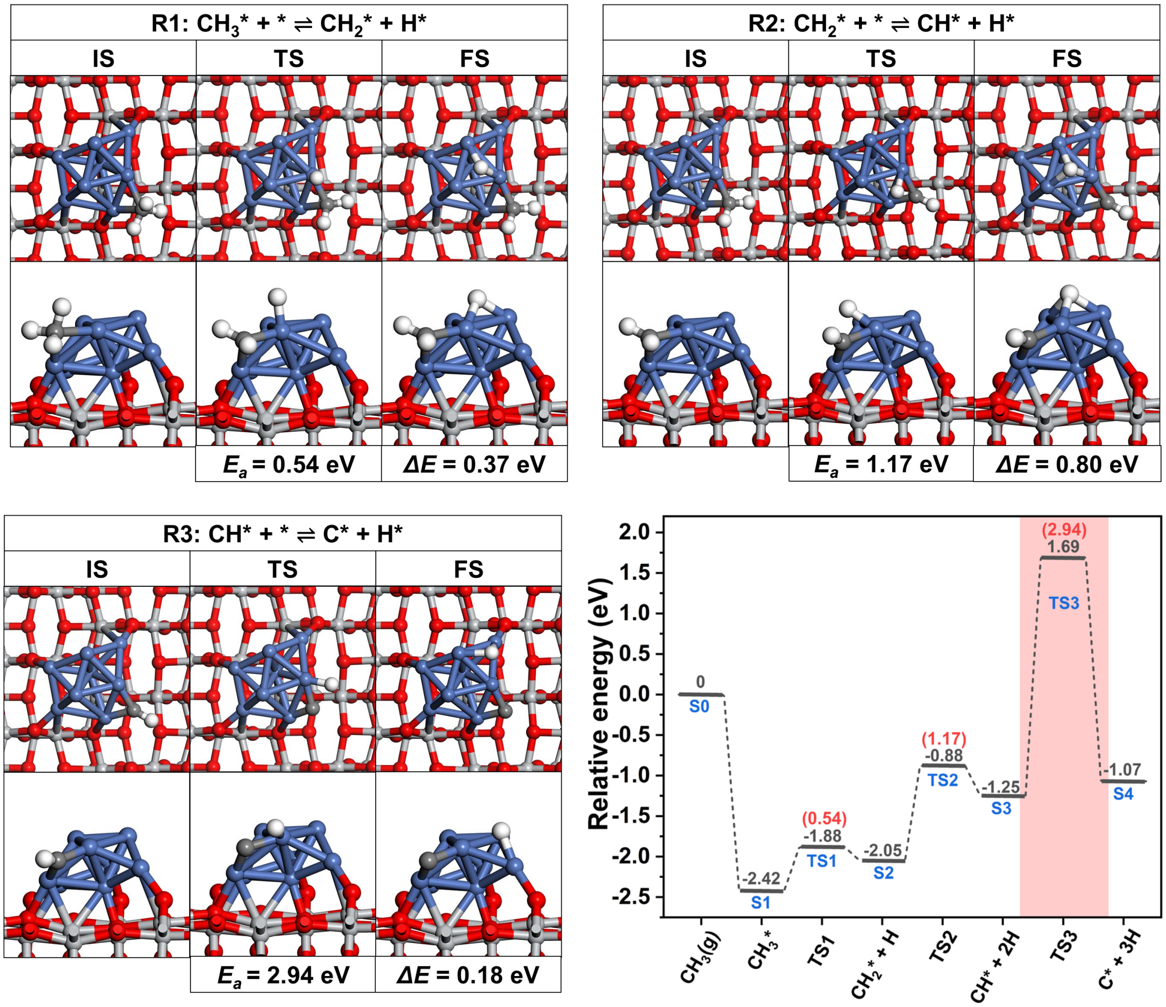

Supplementary Figure 75. DFT studies for methyl dehydrogenation on Ni_8/TiO_{2-x}. Calculated potential energy diagram and corresponding geometric structures for methyl successive dehydrogenation processes over Ni_8/TiO_{2-x} model (IS, TS and FS represent the initial state, transition state and final state, respectively; black and red numbers represent adsorption energy and reaction energy barrier, respectively).

Supplementary Note 24

Based on the experimental results of CH_x reforming, the successive dissociation of methyl group was calculated. The C−H bond breakage derived from CH_3 gives an energy barrier of 0.64 eV and 0.54 eV on Rh_1Ni_7/TiO_{2-x} and Ni_8/TiO_{2-x}, respectively (Supplementary Fig. 74,75: R1 and Supplementary Table 8). For the dehydrogenation from CH_2 to CH, the energy barriers are 0.87 and 1.17 eV for Rh_1Ni_7/TiO_{2-x} and Ni_8/TiO_{2-x}, respectively (Supplementary Fig. 74,75: R2 and Supplementary Table 8). In contrast, the fracture of C−H bond in CH gives a much higher energy barrier of 2.79 and 2.94 Furthermore, as proved by *in situ* DRIFT and DFT calculation results (Supplementary Figs. 46, 67 and 71), the CH species could react with active oxygen to generate formate intermediate. The lower energy requirement for oxidation of CH than C−H bond cleavage indicates that its complete dehydrogenation to produce surface C species is hard, which is consistent with the *in situ* Raman results (Supplementary Fig. 48). Finally, the formate experiences decomposition to produce CO_2 and H_2.

Supplementary Table 1. Metal loadings and physicochemical properties for various samples

Sample	Concentration of element/wt.%[a]			Physicochemical properties[b]		
	Rh	Ni	Ti	$S_{BET}/m^2\ g^{-1}$	$V_{Pore}/cm^3\ g^{-1}$	D_{Pore}/nm
**Ni/TiO_2**	-	61.8	18.4	83.98	0.14	5.36
**$0.1RhNi/TiO_2$**	0.093	63.7	17.3	68.40	0.13	6.60
**$0.3RhNi/TiO_2$**	0.271	63.4	17.8	54.99	0.08	5.13
**$0.5RhNi/TiO_2$**	0.414	63.5	17.3	60.48	0.10	5.50
**$0.8RhNi/TiO_2$**	0.694	64.2	17.6	58.66	0.11	7.05
**$1.0RhNi/TiO_2$**	0.826	63.7	17.6	51.31	0.09	5.71
**Rh/Ni**	0.383	91.3	-	62.74	0.14	7.64
**Rh/TiO_2**	0.372	-	52.2	10.57	0.03	8.29

[a] Results from ICP-AES analysis.
[b] Results from N_2 adsorption-desorption experiment.

Supplementary Table 2. Catalytic performance for ESR reaction over various Ni based catalysts

Catalysts	Reaction temp (°C)	S/C	X_{EtOH} (%)	S_{H_2} (%)[a]	Y_{H_2} (%)	Ref.
**0.5RhNi/TiO_2**	400	3	100	77	62	This work
**Pt-Ni/CeO_2-SiO_2**	400	2	100	-	29	32
**10Ni/CNTs-SF**	500	2.25	100	-	50	33
**Ni-Cu-Cs/$LiAlO_2$**	350	4.5	100	62	13	34
**$Ni_{0.25}$Sn/CeO_2**	400	2.5	50	27	-	35
**K/NiCu-HTlc**	387	5	90	-	40	36
**Ni/CeO_2**	420	6	100	72	-	37
**NiRh/CeO_2**	375	2	100	-	38	38
**30Ni@CeO_2**	400	1.5	65	-	-	39
**Ni-$CeLa_{0.2}$**	550	2	87.6	-	-	40
**Cu_1Ni_9/YSZ**	450	1.5	92	48	27	41
**NiPt/CeO_2**	400	1.5	100	-	22	42
**$Ni_{0.95}Mo_{0.05}$/SBA-15**	450	1.5	90	22	27	43
**Ni-Co/SBA-15**	500	1.5	85.6	62	-	44
**Ni/MMT-TiO_2**	500	5	89	62	55	45
**10Ni-$CePr_{0.20}$**	500	2	70.8	-	-	46
**10ss-5NiO**	400	3	31	82	-	12
**Ni-0.1Ce/CeO_2**	500	2	100	71	-	47

[a] The selectivity of H_2 was calculated based on the detected products.

Supplementary Table 3. XPS results of various samples

Sample	Surface atomic ratio		Relative concentration/%	
	Ti/Ni	Rh/Ni	$Ti^{3+}/(Ti^{3+}+Ti^{4+})$	$O_\beta/(O_\alpha+O_\beta+O_\gamma)$
**Ni/TiO_2**	0.35	-	19.2	32.7
**$0.1RhNi/TiO_2$**	0.78	0.01	14.6	38.7
**$0.3RhNi/TiO_2$**	0.54	0.01	12.2	42.0
**$0.5RhNi/TiO_2$**	0.42	0.02	9.2	45.1
**$0.8RhNi/TiO_2$**	0.40	0.03	8.1	40.2
**$1.0RhNi/TiO_2$**	0.47	0.04	6.7	34.4
**Rh/Ni**	-	0.05	-	30.4
**Rh/TiO_2**	-	-	3.1	17.9

Supplementary Table 4. EXAFS fitting parameters at the Ni K-edge for various samples

Sample	Shell	R (Å)[a]	CN[b]	σ^2 (10^{-3}Å^2)[c]	ΔE_0 (eV)[d]	R factor (%)[e]
**Ni-foil**	Ni–Ni	2.49	12.0	6.0	6.53	0.1
**Rh/Ni**	Ni–Ni	2.49	8.4	5.0	6.80	0.3
**Ni/TiO_2**	Ni–Ni	2.46	7.2	5.1	1.60	1.5
**0.5RhNi/TiO_2**	Ni–Ni	2.43	7.0	5.0	3.38	1.3
**1.0RhNi/TiO_2**	Ni–Ni	2.43	6.7	5.2	4.10	1.6
**NiO**	Ni–O	2.07	5.7	6.6	−3.20	0.9
	Ni–Ni	2.95	11.8	4.1		

[a] bond distance;
[b] coordination number;
[c] Debye-Waller factor;
[d] the inner potential correction;
[e] goodness of fit.

Supplementary Table 5. EXAFS fitting parameters at the Ti K-edge for various samples

Sample	Shell	R (Å)[a]	CN[b]	σ^2 (10^{-3} Å^2)[c]	ΔE_0 (eV)[d]	R factor (%)[e]
**Ti-foil**	Ti–Ti	2.90	6.0	7.4	3.90	1.2
**Rh/TiO_2**	Ti–O	1.95	5.5	4.7	−0.59	1.0
	Ti–Ti	3.05	4.9	2.5		
**Ni/TiO_2**	Ti–O	1.95	4.5	7.1	−1.44	1.3
	Ti–Ti	3.07	2.8	7.8		
**0.5RhNi/TiO_2**	Ti–O	1.95	3.8	9.3	−1.71	1.0
	Ti–Ti	3.06	2.6	8.3		
**1.0RhNi/TiO_2**	Ti–O	1.94	4.1	8.7	−1.17	0.6
	Ti–Ti	3.05	3.2	7.9		
**Anatase**	Ti–O	1.95	6.0	6.4	−2.96	0.6
	Ti–Ti	3.06	5.1	6.2		

[a] bond distance;
[b] coordination number;
[c] Debye-Waller factor;
[d] the inner potential correction;
[e] goodness of fit.

Supplementary Table 6. EXAFS fitting parameters at the Ni K-edge for 0.5RhNi/TiO_2 catalyst in various atmosphere

Sample	Shell	R (Å)[a]	CN[b]	σ^2 (10^{-3}Å^2)[c]	ΔE_0 (eV)[d]	R factor (%)[e]
**Ni-foil**	Ni–Ni	2.49	12.0	6.0	6.53	0.1
**0.5RhNi/TiO_2-H_2**	Ni–Ni	2.45	8.3	9.0	5.87	1.6
**0.5RhNi/TiO_2-CO-1**	Ni–Ni	2.44	8.0	9.5	5.25	0.4
**0.5RhNi/TiO_2-H_2O**	Ni–Ni	2.40	7.7	10.0	−7.75	1.1
**0.5RhNi/TiO_2-CO-2**	Ni–Ni	2.45	7.8	10.2	5.53	0.5
**NiO**	Ni–O	2.07	5.7	6.6	−3.20	0.9
	Ni–Ni	2.95	11.8	4.1		

[a] bond distance;
[b] coordination number;
[c] Debye-Waller factor;
[d] the inner potential correction;
[e] goodness of fit.

Supplementary Table 7. EXAFS fitting parameters at the Ni K-edge for Rh/Ni catalyst in various atmosphere

Sample	Shell	R (Å)[a]	CN[b]	σ^2 (10^{-3}Å^2)[c]	ΔE_0 (eV)[d]	R factor (%)[e]
**Ni-foil**	Ni–Ni	2.49	12.0	6.0	6.53	0.1
**Rh/Ni-H_2**	Ni–Ni	2.49	8.0	9.0	7.07	0.6
**Rh/Ni -CO**	Ni–Ni	2.45	7.2	9.4	−8.27	1.4
**Rh/Ni-H_2O**	Ni–O	2.07	0.4	6.6	−6.00	0.8
	Ni–Ni	2.46	7.5	6.6		
**NiO**	Ni–O	2.07	5.7	6.6	−3.20	0.9
	Ni–Ni	2.95	11.8	4.1		

[a] bond distance;
[b] coordination number;
[c] Debye-Waller factor;
[d] the inner potential correction;
[e] goodness of fit.

Supplementary Table 8. Calculated activation energy (E_a) and reaction energy (ΔE) of various elementary reactions in ESR over Rh_1Ni_7/TiO_{2-x} and Ni_8/TiO_{2-x} systems

**Elementary reaction**		**Rh_1Ni_7/TiO_{2-x}**		**Ni_8/TiO_{2-x}**	
		**E_a (eV)**	**ΔE (eV)**	**E_a (eV)**	**ΔE (eV)**
**CH_3CH_2OH dehydrogenation *via* Route 1**	$CH_3CH_2OH^* \rightarrow CH_3CH_2O^*$	1.03	-1.00	0.59	-0.45
	$CH_3CH_2O^* \rightarrow CH_3CHO^*$	1.01	-0.01	0.86	0.07
	$CH_3CHO^* \rightarrow CH_3CO^*$	0.72	0.08	1.65	1.46
**CH_3CH_2OH dehydrogenation *via* Route 2**	$CH_3CH_2OH^* \rightarrow CH_3CHOH^*$	4.06	1.29	2.63	1.68
	$CH_3CHOH^* \rightarrow CH_3CHO^*$	3.35	-0.23	0.97	-1.58
	$CH_3CHO^* \rightarrow CH_3CO^*$	0.83	-0.45	1.64	1.21
**CH_3CH_2OH dehydrogenation *via* Route 3**	$CH_3CH_2OH^* \rightarrow CH_3CHOH^*$	4.06	1.29	2.63	1.68
	$CH_3CHOH^* \rightarrow CH_3COH^*$	0.53	-0.32	3.18	0.74
	$CH_3COH^* \rightarrow CH_3CO^*$	0.78	-0.79	0.63	-1.16
**CH_3 dehydrogenation in CH_3CH_2OH**	$CH_3CH_2OH^* \rightarrow CH_2CH_2OH^*$	2.03	1.53	2.18	0.80
**C-C bond cleavage**	$CH_3CO^* \rightarrow CH_3^* + CO^*$	1.17	-0.45	1.31	-0.69
**CH_3 successive dehydrogenation**	$CH_3^* \rightarrow CH_2^* + H$	0.64	0.38	0.54	0.37
	$CH_2^* \rightarrow CH^* + H$	0.87	-0.53	1.17	0.80
	$CH^* \rightarrow C^* + H$	2.79	-0.36	2.94	0.18
**CO disproportionation**	$CO^* \rightarrow C^* + O^*$	1.91	0.29	2.17	0.62
	$C^* + O^* + CO^* \rightarrow C^* + CO_2^*$	1.88	0.22	2.30	0.62
**CO_2 desorption**	$CO_2^* \rightarrow CO_2(g)$	-	0.65	-	0.93
**H_2O dissociation to active oxygen**	$C^* + H_2O^* \rightarrow CH^* + OH^*$	0.27	-1.37	0.30	-0.68
	$OH^* \rightarrow O^* + H$	0.94	-1.45	1.50	-0.20
	$CH^* + 2OH^* + H \rightarrow CH^* + O^* + H + H_2O^*$	0.24	-0.21	0.65	-0.24
**H_2O desorption**	$H_2O^* \rightarrow H_2O(g)$	-	2.30	-	3.54
**Formate generation *via* HCO***	$CH^* + O^* \rightarrow HCO^*$	2.36	1.18	1.63	0.84
	$HCO^* + O^* \rightarrow HCOO^*$	1.28	0	2.79	0.52
	$HCOO^* \rightarrow CO_2^* + H$	1.20	1.07	2.71	1.29
**Formate generation *via* COH***	$C^* + OH^* \rightarrow COH^*$	3.28	1.22	4.04	1.01
	$COH^* + O \rightarrow COOH^*$	0.91	-0.17	3.59	0.75
	$COOH^* \rightarrow HCOO^*$	4.16	0.26	-	-
**CO_2 desorption**	$CO_2^* \rightarrow CO_2(g)$	-	2.58	-	2.67

Supplementary References

1. Fogler H. S., Elements of Chemical Reaction Engineering, Fourth ed., New Jersey, 2006, 815-841.

2. James G. Speight, Lange's handbook of chemistry, Sixteenth Edition., Laramie, Wyoming, 2005, 1453.

3. Zhao, Y. et al. Highly dispersed TiO_6 units in a layered double hydroxide for water splitting. *Chemistry* **18**, 11949-11958 (2012).

4. Zhao, Y. et al. NiTi-Layered double hydroxides nanosheets as efficient photocatalysts for oxygen evolution from water using visible light. *Chem. Sci.* **5**, 951-958 (2014).

5. Chen, S., Pei, C. & Gong, J. Insights into interface engineering in steam reforming reactions for hydrogen production. *Energ. Environ. Sci.* **12**, 3473-3495 (2019).

6. Zanchet, D., Santos, J. B. O., Damyanova, S. Gallo, J. M. R. Bueno, J. M. C. Toward understanding metal-catalyzed ethanol reforming. *ACS Catal.* **5**, 3841-3863 (2015).

7. Crowley, S. & Castaldi, M. J. Mechanistic insights into catalytic ethanol steam reforming using isotope-labeled reactants. *Angew. Chem. Int. Ed.* **55**, 10650-10655 (2016).

8. Greluk, M., Gac, W. Rotko, M., Słowik, G. & Turczyniak-Surdacka, S. Co/CeO_2 and Ni/CeO_2 catalysts for ethanol steam reforming: Effect of the cobalt/nickel dispersion on catalysts properties. *J. Catal.* **393**, 159-178 (2021).

9. Xu, W. Steam reforming of ethanol on Ni/CeO_2: Reaction pathway and interaction between Ni and the CeO_2 Support. *ACS Catal.* **3**, 975-984 (2013).

10. Ferencz, Z. Effects of support and Rh additive on Co-based catalysts in the ethanol steam reforming reaction. *ACS Catal.* **4**, 1205-1218 (2014).

11. Gu, J. Synergizing metal–support interactions and spatial confinement boosts dynamics of atomic nickel for hydrogenations. *Nat. Nanotechnol.* **16**, 1141-1149 (2021).

12. Tian, H. et al. Tunable metal-oxide interaction with balanced Ni^0/Ni^{2+} sites of $Ni_xMg_{1-x}O$ for ethanol steam reforming. *Appl. Catal. B-Environ.* **293**, 120178 (2021).

13. Galhardo, T. S. et al. Optimizing active sites for high CO selectivity during CO_2 hydrogenation over supported nickel catalysts. *J. Am. Chem. Soc.* **143**, 4268-4280 (2021).

14. Chen, Y. et al. Engineering the atomic interface with single platinum atoms for enhanced photocatalytic hydrogen production. *Angew. Chem. Int. Ed.* **59**, 1295-1301 (2020).

15. Berkó, A., Balázs, N., Kassab, G. &Óvári, L. Segregation of K and its effects on the growth, decoration, and adsorption properties of Rh nanoparticles on TiO_2(110). *J. Catal.* **289**, 179-189 (2012).

16. Gao, X. et al. Synergistic introducing of oxygen vacancies and hybrid of organic semiconductor: Realizing deep structure modulation on Bi_5O_7I for high-efficiency photocatalytic pollutant oxidation. *Appl. Catal. B-Environ.* **265**, 118562 (2020).

17. Jia, R. et al. Boosting selective nitrate electroreduction to ammonium by constructing oxygen vacancies in TiO_2. *ACS Catal.* **10**, 3533-3540 (2020).

18. Li, K. Enhanced electrocatalytic performance for the hydrogen evolution reaction through surface enrichment of platinum nanoclusters alloying with ruthenium in situ embedded in carbon. *Energ. Environ. Sci.* **11**, 1232-1239 (2018).

19. Li, K. et al. One-nanometer-thick PtNiRh trimetallic nanowires with enhanced oxygen reduction electrocatalysis in acid media: Integrating multiple advantages into one catalyst. *J. Am. Chem. Soc.* **140**, 16159-16167 (2018).

20. Li, M. M.-J. et al. Methanol synthesis at a wide range of H_2/CO_2 ratios over a Rh-In bimetallic catalyst. *Angew. Chem. Int. Ed.* **59**, 16039-16046 (2020).

21. Lu, J. et al. Weakening the metal-support strong interaction to enhance catalytic performances

of alumina supported Ni-based catalysts for producing hydrogen. *Appl. Catal. B-Environ.* **263**, 118177 (2020).

22. Zhang, J. et al $Ni^0/Ni^{\delta+}$ synergistic catalysis on a nanosized Ni surface for simultaneous formation of C–C and C–N bonds. *ACS Catal.* **9**, 11438-11446 (2019).

23. Yin, H. et al. Nanometre-scale spectroscopic visualization of catalytic sites during a hydrogenation reaction on a Pd/Au bimetallic catalyst. *Nat. Catal.* **3**, 834-842 (2020).

24. Schwartz, V. et al. Structural investigation of Au catalysts on TiO_2-SiO_2 supports: Nature of the local structure of Ti and Au atoms by EXAFS and XANES. *J. Phys. Chem. C.* **111**, 17322-17332 (2007).

25. Liu, Z. Ambient pressure XPS and IRRAS investigation of ethanol steam reforming on Ni–CeO_2(111) catalysts: An in situ study of C–C and O–H bond scission. *Phys. Chem. Chem. Phys.* **18**, 16621-16628 (2016).

26. Yang, X. Low pressure CO_2 hydrogenation to methanol over gold nanoparticles activated on a CeO_x/TiO_2 interface. *J. Am. Chem. Soc.* **137**, 10104-10107 (2015).

27. Li,X. et al. Controlling CO_2 hydrogenation selectivity by metal-supported electron transfer. *Angew. Chem. Int. Ed.* **59**, 19983-19989 (2020).

28. Pramhaas, V. et al. Interplay between CO disproportionation and oxidation: On the origin of the CO reaction onset on atomic layer deposition-grown Pt/ZrO_2 model catalysts. *ACS Catal.* **11**, 208-214 (2021).

29. Olmos-Asar, J. A. et al. CO on supported Cu nanoclusters: Coverage and finite size contributions to the formation of carbide via the boudouard process. *ACS Catal.* **5**, 2719-2726 (2015).

30. Yao, S. et al. Rodriguez José, D. Ma, Atomic-layered Au clusters on α-MoC as catalysts for the low-temperature water-gas shift reaction. *Science* **357**, 389-393 (2017).

31. Luo, W., Chen, Y., Du, Z. & Chen, C. Theoretical study on PdCu/CeO_2-catalyzed water–gas shift reaction: Crucial role of the metal/ceria interface and O_2 enhancement effects. *J. Phys. Chem. C.* **122**, 28868-28883 (2018).

32. Palma V., Ruocco, C., Meloni, E. & Ricca, A. Highly active and stable Pt-Ni/CeO_2-SiO_2 catalysts for ethanol reforming. *J. Clean. Prod.* **166**, 263-272 (2017).

33. Prasongthum, N. et al. Highly active and stable Ni supported on CNTs-SiO_2 fiber catalysts for steam reforming of ethanol. *Fuel. Process. Technol.* **160**, 185-195 (2017).

34. Özkan, G. Şahbudak, B. Özkan, G. Effect of molar ratio of water/ethanol on hydrogen selectivity in catalytic production of hydrogen using steam reforming of ethanol. *Int. J. Hydrogen. Energ.* **44**, 9823-9829 (2019).

35. Tian, H., Li, X., Chen, S., Zeng,L. & Gong, J. Role of Sn in Ni-Sn/CeO_2 catalysts for ethanol steam reforming. *Chinese. J. Chem.* **35**, 651-658 (2017).

36. Cunha, A. F., Wu, Y.-J., Li, P., Yu, J.-G. & Rodrigues, A. E. Sorption-enhanced steam reforming of ethanol on a novel K–Ni–Cu–hydrotalcite hybrid material. *Ind. Eng. Chem. Res.* **53**, 3842-3853 (2014).

37. Greluk, M., Gac, W., Rotko, M., Słowik, G. & Turczyniak-Surdacka, S. Co/CeO_2 and Ni/CeO_2 catalysts for ethanol steam reforming: Effect of the cobalt/nickel dispersion on catalysts properties. *J. Catal.* **393**, 159-178 (2021).

38. Kugai, J., Velu, S. & Song, C. Low-temperature reforming of ethanol over CeO_2-supported Ni-Rh bimetallic catalysts for hydrogen production. *Catal. Lett.* **101**, 255-264 (2005).

39. Wang F., et al. Embedded Ni catalysts in Ni-O-Ce solid solution for stable hydrogen production

from ethanol steam reforming reaction. *Fuel. Process. Technol.* **193**, 94-101 (2019).

40. Xiao, Z. et al. Boosting hydrogen production from steam reforming of ethanol on nickel by lanthanum doped ceria. *Appl. Catal. B-Environ.* **286**, 119884 (2021).

41. Chen, F. et al. Ni-Cu bimetallic catalysts on Yttria-stabilized zirconia for hydrogen production from ethanol steam reforming. *Fuel* **280**, 118612 (2020).

42. Palma, V., Castaldo, F., Ciambelli, P. & Iaquaniello, G. CeO_2-supported Pt/Ni catalyst for the renewable and clean H_2 production via ethanol steam reforming. *Appl. Catal. B-Environ.* **145**, 73-84 (2014).

43. Kim, D., Kwak, B. S., Park, N. -K., Han, G. B. & Kang, M. Dynamic hydrogen production from ethanol steam-reforming reaction on Ni_xMo_y/SBA-15 catalytic system. *Int. J. Energ. Res.* **39**, 279-292 (2015).

44. Rodriguez-Gomez, A. & Caballero, A. Bimetallic Ni-Co/SBA-15 catalysts for reforming of ethanol: How cobalt modifies the nickel metal phase and product distribution. *Mol. Catal.* **449**, 122-130 (2018).

45. Mulewa, W., Tahir, M. & Amin, N. A. S. MMT-supported Ni/TiO_2 nanocomposite for low temperature ethanol steam reforming toward hydrogen production. *Chem. Eng. J.* **326**, 956-969 (2017).

46. Xiao, Z. Engineering oxygen vacancies and nickel dispersion on CeO_2 by Pr doping for highly stable ethanol steam reforming. *Appl. Catal. B-Environ.* **258**, 117940 (2019).

47. Greluk, M. Rotko, M. Turczyniak-Surdacka, S. Comparison of catalytic performance and coking resistant behaviors of cobalt- and nickel based catalyst with different Co/Ce and Ni/Ce molar ratio under SRE conditions. *Appl. Catal. A-Gen.* **590**, 117334 (2020).
